# Supplementary material for: Macrophages induce malignant traits in mammary epithelium via IKKε/TBK1 kinases and the serine biosynthesis pathway
Source: EMBO Mol Med. 2020 Jan 13;12(2):e10491. doi: 10.15252/emmm.201910491 (PMC7005540; doi:10.15252/emmm.201910491)
Supplement: Supplementary file 1 — Expanded View Figures PDF [file EMMM-12-e10491-s001.pdf]

Expanded View Figures

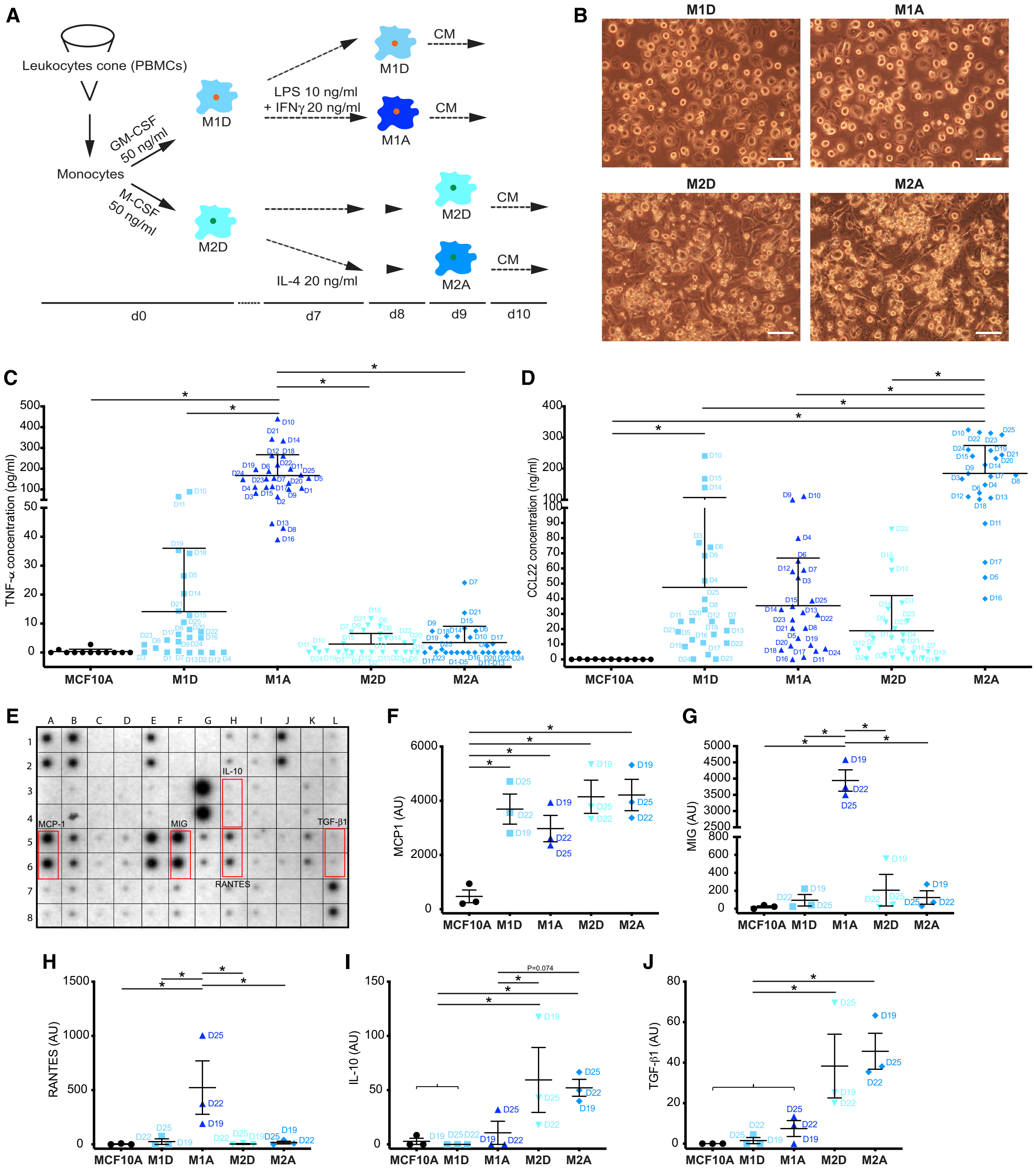

Figure EV1.

◀

Figure EV1. Characterization of M1 and M2 macrophages derived from human healthy donors.

A Schematic representing the protocol used to differentiate human primary monocytes from healthy human donors into macrophages.

B Representative images showing differentiated macrophages in culture. M1D stands for M1-differentiated macrophages, M1A for M1-activated macrophages, M2D for M2-differentiated macrophages and M2A for M2-activated macrophages. Scale bar: 100 μm.

C, D TNF-α levels (C) and CCL-22 levels (D) in macrophage-conditioned media across different macrophage donors compared to control medium as determined with ELISA. Macrophage donors are indicated as D1-D25. Lines and error bars represent mean and SD.

E–J Human cytokine array of macrophage-conditioned or control media. (E) Example of cytokine array dot blot (M1A) with selected cytokines highlighted in red brackets. (F–J) Different expression profile of selected cytokines across macrophage populations. Lines and error bars represent mean ± SEM from three independent experiments.

Data information: $^{*}P < 0.05$ as measured by one-way ANOVA with uncorrected Fisher's LSD *post hoc* test (exact *P* values are shown in Table EV3).
Source data are available online for this figure.

 © 2020 The Authors

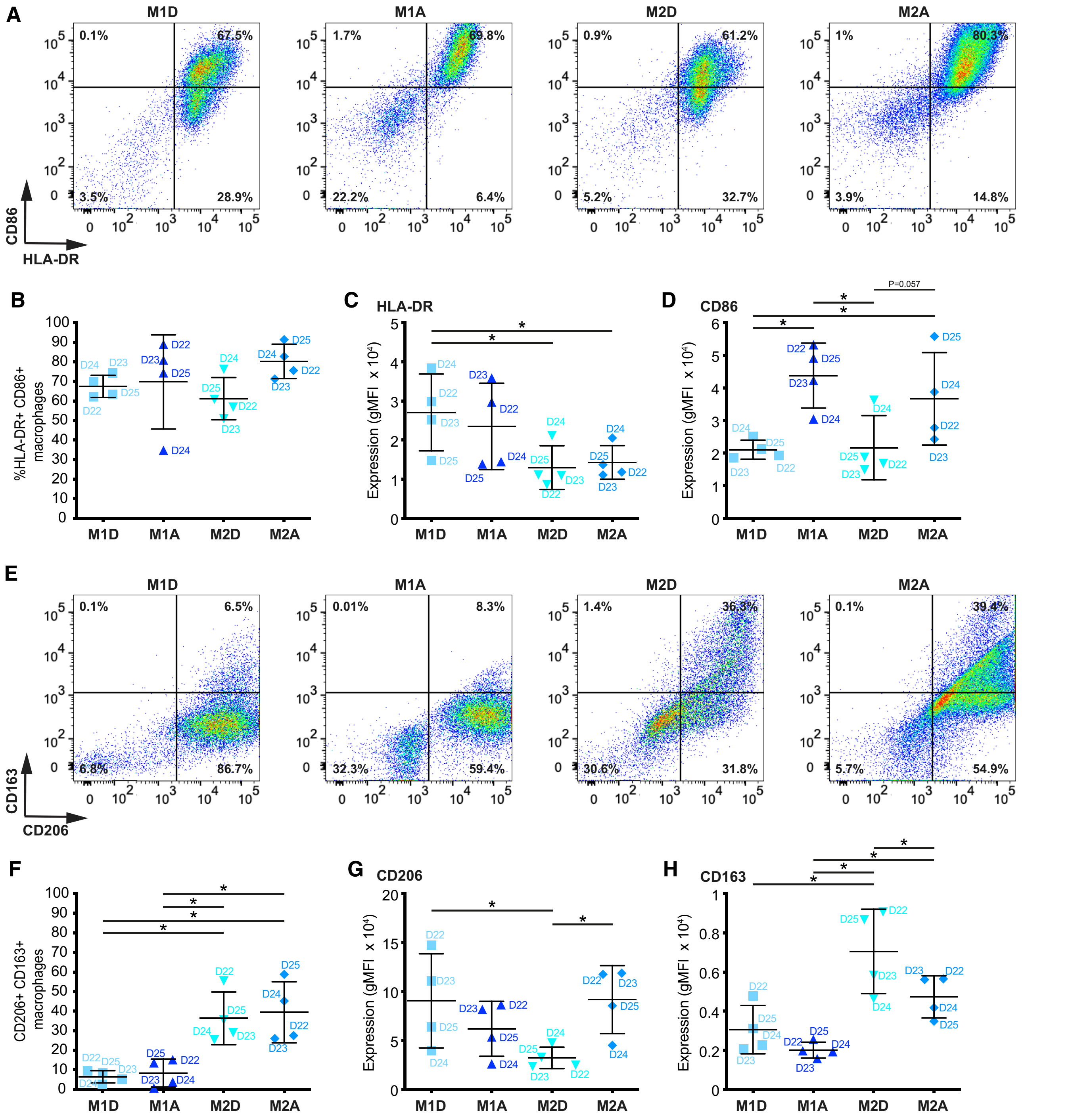

Figure EV2.

◀ **Figure EV2. Flow cytometry analysis of macrophage populations.**

A Representative images showing FACS dot plots for HLA-DR^{+}/CD86^{+} macrophages. Average frequencies of 4 donors are shown.

B Quantification of HLA-DR^{+}/CD86^{+} macrophages across macrophage populations. Lines and error bars represent mean ± SD from two independent experiments (2 macrophage donors each).

C, D Expression levels (geometric mean of fluorescent intensity, gMFI) of (C) HLA-DR and (D) CD86 in HLA-DR^{+}/CD86^{+} macrophages across macrophage populations. Lines and error bars represent mean ± SD from two independent experiments (2 macrophage donors each).

E Representative images showing FACS dot plots for CD206^{+}/CD163^{+} macrophages. Average frequencies of 4 donors are shown.

F Quantification of CD206^{+}/CD163^{+} macrophages across macrophage populations. Lines and error bars represent mean ± SD from two independent experiments (2 macrophage donors each).

G, H Expression levels (geometric mean of fluorescent intensity, gMFI) of (G) CD206 and (H) CD163 in CD206^{+}/CD163^{+} macrophages across macrophage populations. Lines and error bars represent mean ± SD from two independent experiments (2 macrophage donors each).

Data information: Macrophage donors are indicated as D22-D25. M2D—M2-differentiated, M1A—M1-activated, M2D—M2-differentiated, M2A—M2-activated. *$P < 0.05$ as measured by one-way ANOVA with uncorrected Fisher's LSD *post hoc* test (exact *P* values are shown in Table EV3).
Source data are available online for this figure.

© 2020 The Authors

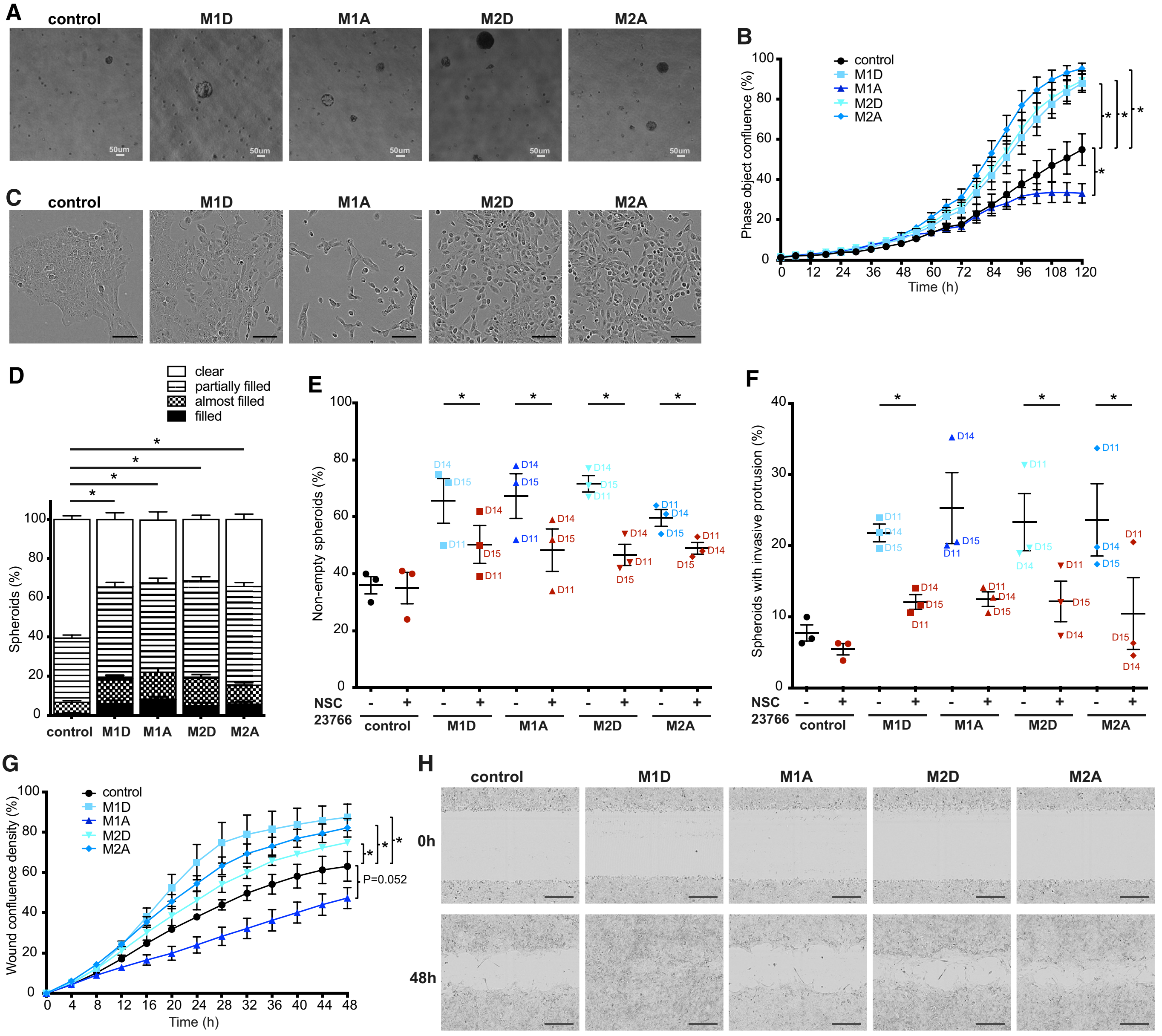
A
control
M1D
M1A
M2D
M2A
B
Phase object confluence (%)
Time (h)
control
M1D
M1A
M2D
M2A
C
D
clear
partially filled
almost filled
filled
Spheroids (%)
E
Non-empty spheroids (%)
NSC 23766
F
Spheroids with invasive protrusion (%)
G
Wound confluence density (%)
P=0.052
H
0h
48h

Figure EV3.

Figure EV3. Transforming effects of macrophage-conditioned medium on MCF10A cells.

M1D—M1-differentiated, M1A—M1-activated, M2D—M2-differentiated, M2A—M2-activated macrophages.

A Representative images of colonies formed by MCF10A cells grown for 5 weeks in macrophage-conditioned medium in soft agar as compared to control. Scale bar: 50 μm.

B, C The effects of macrophage-conditioned medium on MCF10A cell proliferation. (B) M1D, M2D and M2A macrophage-conditioned media enhance proliferation, whereas M1A-conditioned medium reduces proliferation rate. Lines and error bars represent mean ± SEM from five independent experiments where the medium conditioned by D1, D2, D4, D8, D12 and D13 was used ($n \geq 3$ per condition). (C) Representative images of MCF10A cells at 120 h in macrophage-conditioned medium as compared to control. Scale bar: 100 μm.

D–F Sixteen-day-old MCF10A spheroids grown in Matrigel/collagen mix were stimulated for 24 h with either macrophage-conditioned or control medium. (D) Macrophage-conditioned medium induced the filling of the spheroid lumen with cell nuclei compared to control. Filling of the spheroid lumen with cell nuclei categorized into four groups (clear, partially filled, almost filled and filled). Lines and error bars represent mean ± SEM from 11 independent experiments ($n = 2$ per condition; 50 spheroids each). Partially filled, almost filled and filled spheroids were combined together (non-empty spheroids) for statistical analysis. NSC23766 (Rac1 inhibitor; 50 μM) reduces (E) the filling of the lumen with cell nuclei (partially filled, almost filled and filled spheroids were combined together as non-empty spheroids) (F) invasive protrusions of spheroids stimulated with macrophage-conditioned media compared to control. Lines and error bars represent mean ± SEM from three independent experiments. (E) $n = 2$ per condition; 50 spheroids each. (F) $n = 2$ per condition; at least 15 spheroids per condition from at least 2 fields of view. All the data shown without the use of NSC23766 are also included in Fig 1C or Fig 1I, respectively. Macrophage donors are indicated as D11, D14 and D15.

G, H The effect of macrophage-conditioned medium on MCF10A cell migration compared to control medium using wound scratch assay. (G) M1D, M2D and M2A macrophage-conditioned media enhance migration, whereas M1A-conditioned medium reduces migration rate. Lines and error bars represent mean ± SEM from 4 independent experiments ($n = 3$ per condition), where medium conditioned by D1, D2, D10, D11 and D13 was used. (H) Representative images of wounds of MCF10A cells, just after adding conditioned medium (0 h) and at 48 h in macrophage-conditioned medium compared to control. Scale bar: 300 μm.

Data information: Data were analysed by one-way ANOVA with uncorrected Fisher's LSD *post hoc* test (B, D, G) or by two-tailed Student's *t*-test (E, F). $^*P < 0.05$. Exact *P* values are shown in Table EV3.
Source data are available online for this figure.

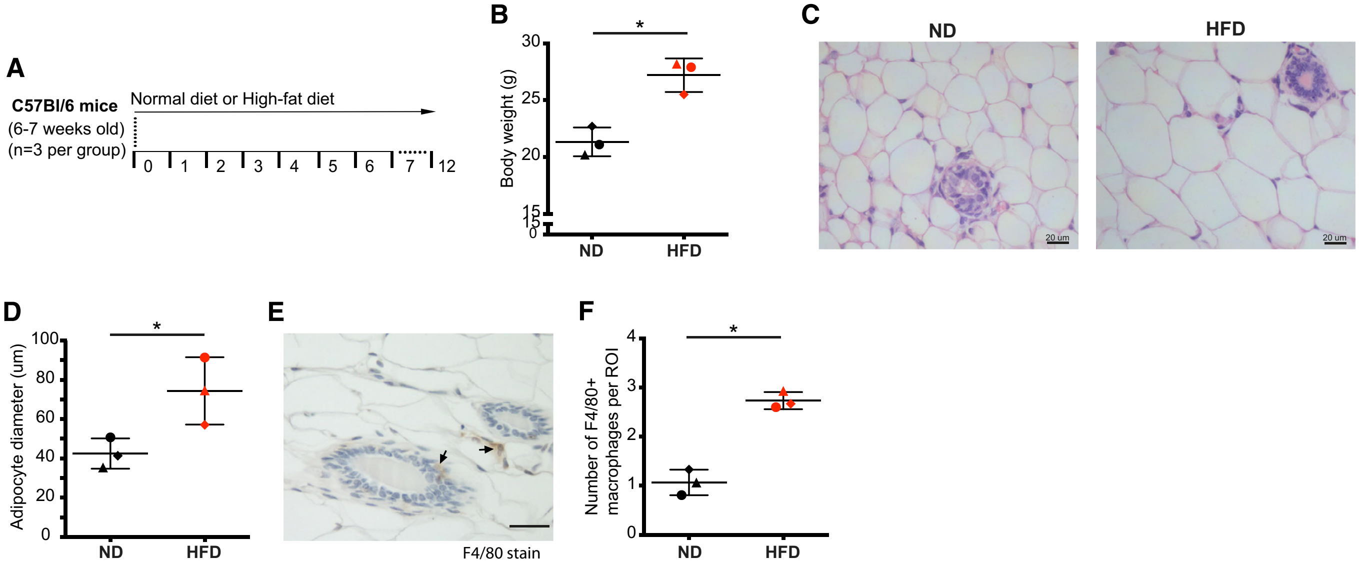

Figure EV4. Characterization of mammary fat pads for organoids study.

A Mammary organoids were isolated from 19- to 20-week-old C57Bl/6 mice that were either on normal (ND) or high-fat diet (HFD) ($n = 3$).

B–D The efficacy of the diet was confirmed by (B) increased body weight on the day of organoids isolation and (C, D) larger adipocyte diameter in mammary fat pads in H&E-stained sections of HFD mice. (C) Representative images of H&E-stained mammary fat pads isolated from either ND or HFD mice. (D) At least 100 adipocytes per mice were scored, and the average major diameter is shown. Each mouse is labelled with a different symbol shape. Lines and error bars represent mean ± SD.

E Representative image showing F4/80 + macrophages (marked with a black arrow) in mice mammary fat pads.

F The number of F4/80 + macrophages in mammary fat pads is higher in HFD mice compared to ND mice. The average of F4/80 + macrophages in at least 10 fields of view per mouse is shown. Each mouse is labelled with a different symbol shape. Lines and error bars represent mean ± SD.

Data information: Scale bar: 20 μm. $^*P < 0.05$ by two-tailed Student's *t*-test (exact *P* values are shown in Table EV3).
Source data are available online for this figure.

© 2020 The Authors

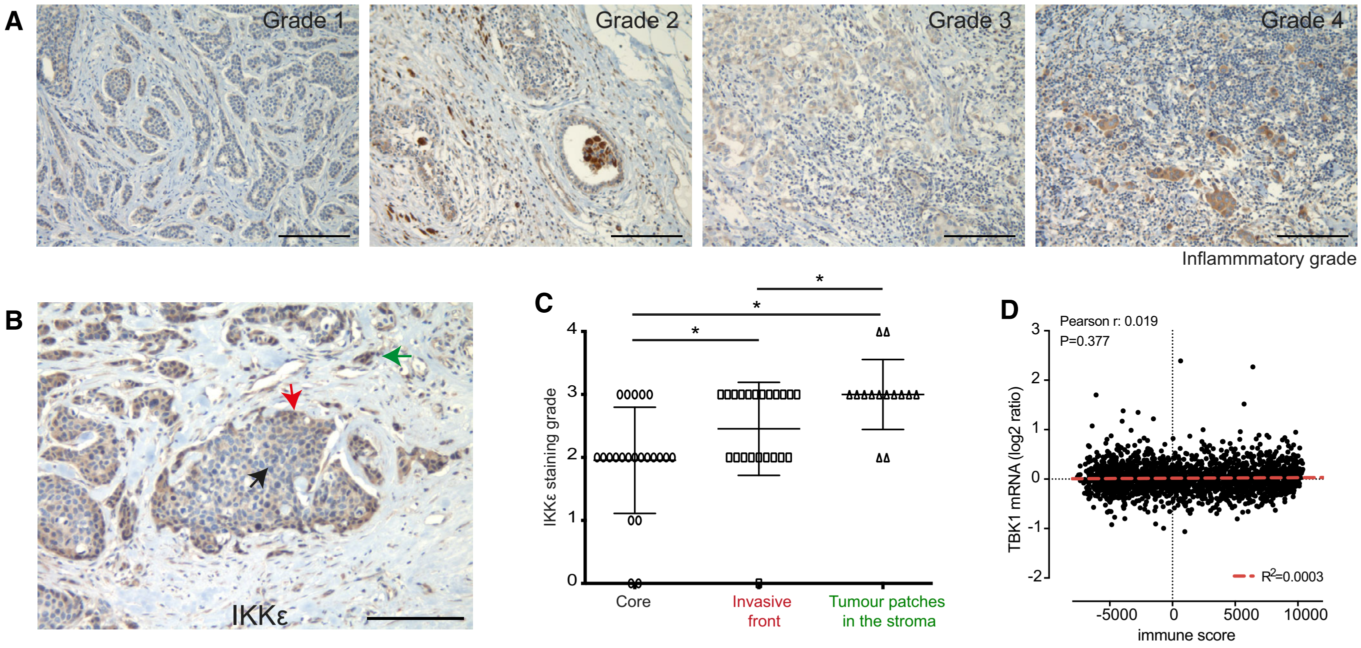

Figure EV5. Semi-quantitative analysis of immune infiltration in a cohort of breast cancer cases and intratumoural analysis of IKKε expression.

A Immune cell infiltration was assessed semi-quantitatively using 0–4 grade scale in tissue sections previously scored for IKKε expression ("0": no inflammatory cells; "1": weak; "2": moderate; "3": strong; "4": very strong). Representative images showing inflammatory grade in human breast carcinomas stained for IKKε.

B, C Intratumoural heterogeneity of IKKε expression in human breast carcinomas. (B) Representative image showing heterogeneity of IKKε expression within tumour. The tumour core was marked with the black arrow, the tumour invasive front with a red arrow and the invasive stromal patches with a green arrow. (C) The expression of IKKε is increased both at the invasive front of tumour and invasive stromal patches compared to the core of tumour ($n = 22$). Lines and error bars represent mean ± SD. *$P < 0.05$ as measured by Kruskal–Wallis test with uncorrected Dunn's *post hoc* test (exact *P* values are shown in Table EV3).

D Correlation of mRNA levels of TBK1 with the immune signature (Yoshihara *et al*, 2013) in the METABRIC transcriptomic dataset from 1981 breast cancer patients (Curtis *et al*, 2012). Pearson's correlation rho coefficient and significance of difference from slope = 0 (*P*) are shown.

Data information: Scale bar: 100 μm.
Source data are available online for this figure.

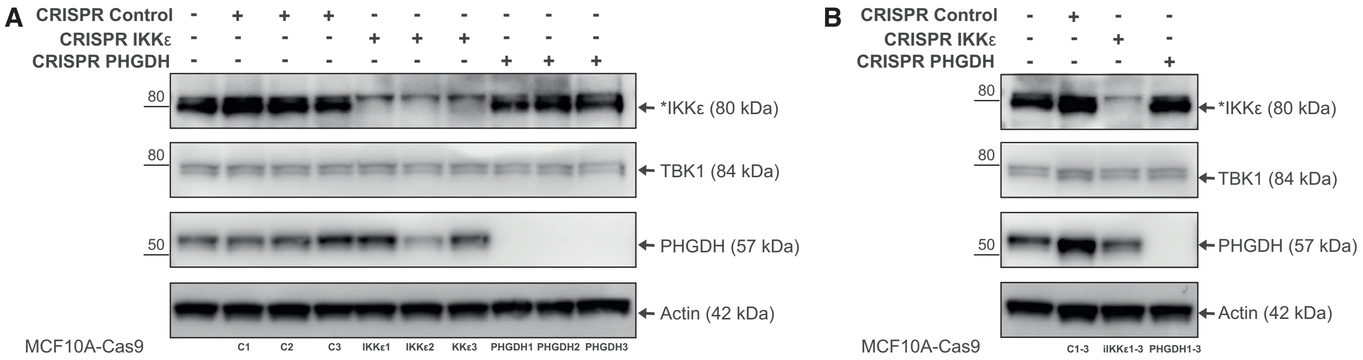

Figure EV6. Deletion of *IKBKE* and *PHDGH* genes via CRISPR-Cas9 technology in MCF10A cells.

CRISPR-Cas9 technology was used to delete *IKBKE* gene encoding for IKKε (CRISPR IKKε) or *PHGDH* (CRISPR PHGDH) in MCF10A cells. In parallel, MCF10A-Cas9 cells treated with control non-targeting crRNA were used to control for potential responses to CRISPR-Cas9 components (CRISPR control).

A Western blot analyses of individual clones (n = 3 per cell line) show knockout of IKKε or PHGDH in MCF10A cells. C1, C2, C3—individual clones of CRISPR-Cas9 MCF10A cells treated with control non-targeting crRNA (CRISPR control). IKKε1, IKKε2, IKKε3—individual clones of IKKε CRISPR-Cas9 knockout MCF10A cells (CRISPR IKKε). PHGDH1, PHGDH2, PHGDH3—individual clones of PHGDH CRISPR-Cas9 knockout MCF10A cells (CRISPR PHGDH).

B Western blot analyses of combined clones show knockout of IKKε or PHGDH in MCF10A cells. Individual clones that were combined are shown in panel (A).

Data information: The levels of IKKε homologous protein TBK1 were not changed in IKKε CRISPR-Cas9 MCF10A cells. β-actin was used as a loading control. * indicates unspecific band in IKKε blot.
Source data are available online for this figure.

 © 2020 The Authors
