# Supplementary figures and images for: Macrophages induce malignant traits in mammary epithelium via IKKε/TBK1 kinases and the serine biosynthesis pathway (part 1 of 2)
Source: EMBO Mol Med. 2020 Jan 13;12(2):e10491. doi: 10.15252/emmm.201910491 (PMC7005540; doi:10.15252/emmm.201910491)

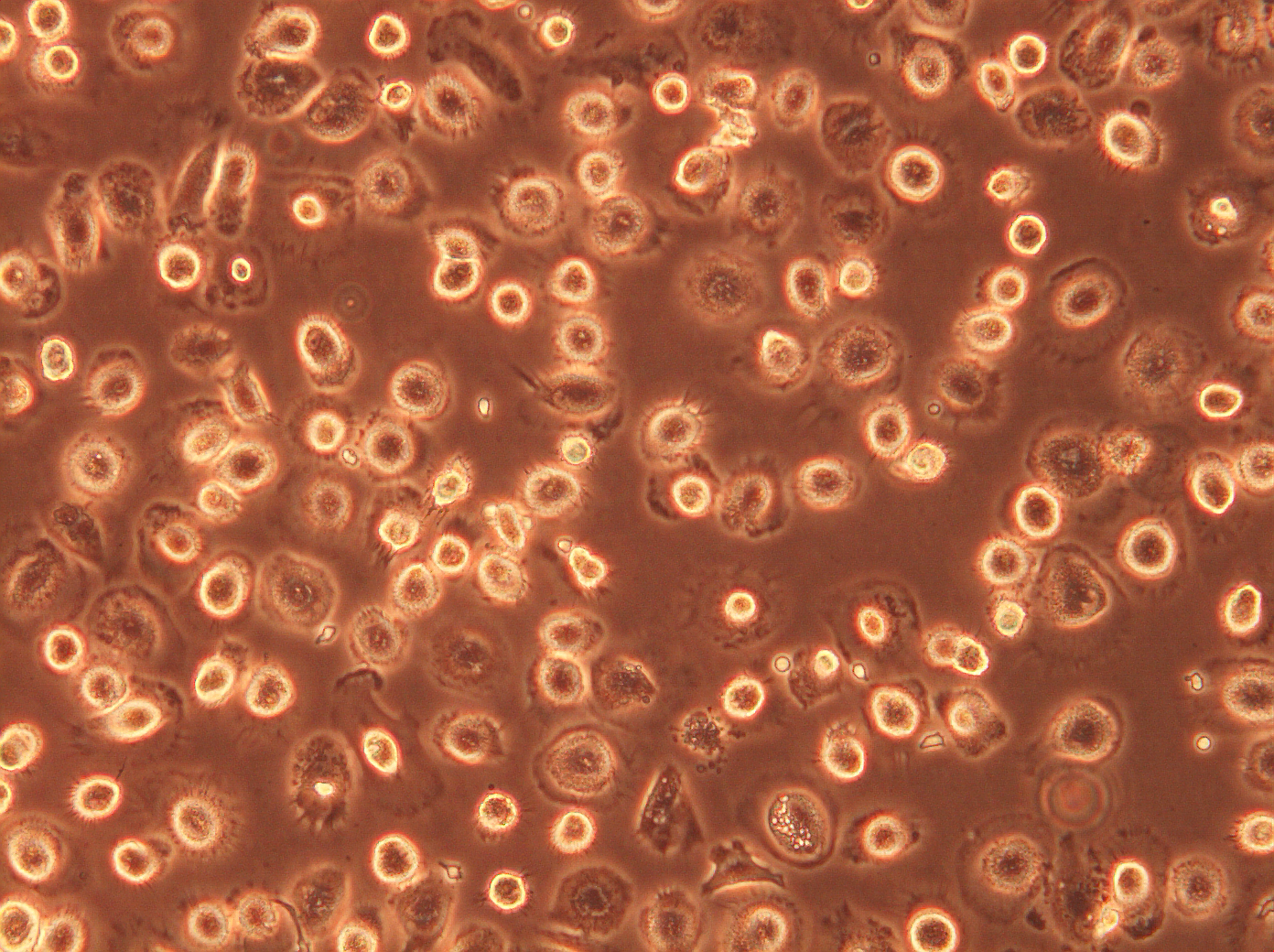

Supplement: Supplementary file 5 — Source Data for Expanded View [file EMMM-12-e10491-s012.zip › EV_source_data/Fig_EV1/Fig_EV1B_M1A.tif]

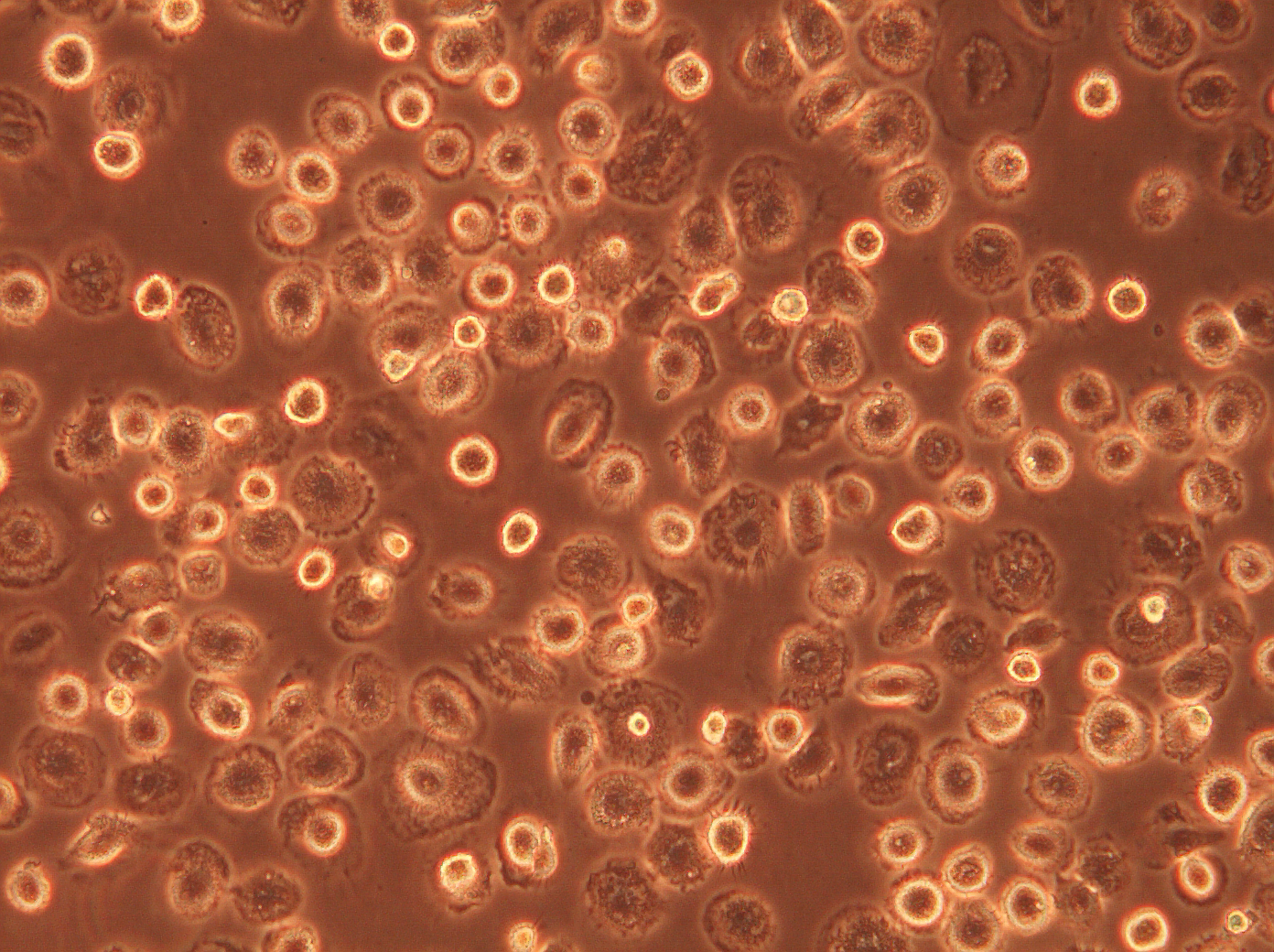

Supplement: Supplementary file 5 — Source Data for Expanded View [file EMMM-12-e10491-s012.zip › EV_source_data/Fig_EV1/Fig_EV1B_M1D.tif]

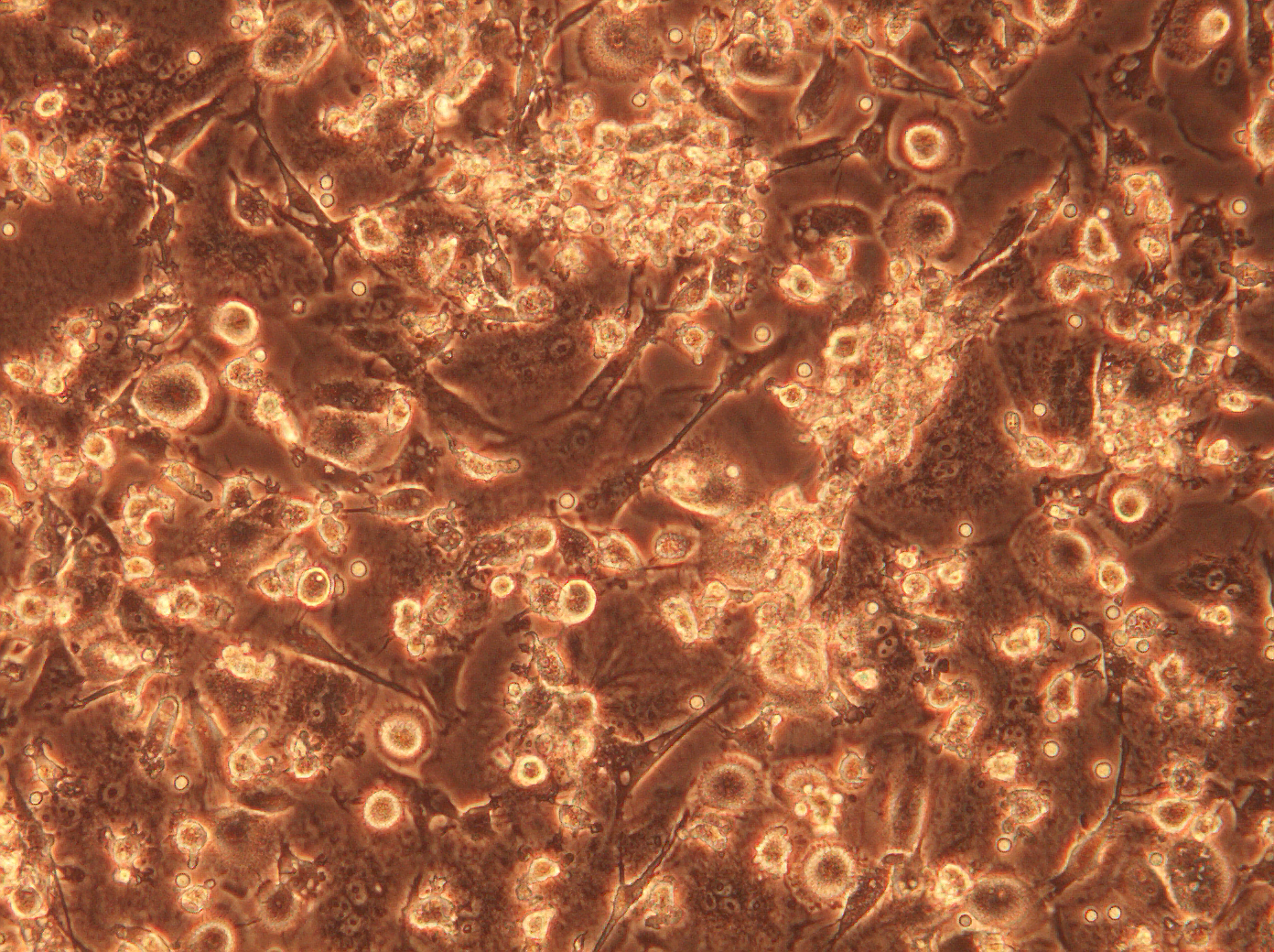

Supplement: Supplementary file 5 — Source Data for Expanded View [file EMMM-12-e10491-s012.zip › EV_source_data/Fig_EV1/Fig_EV1B_M2A.tif]

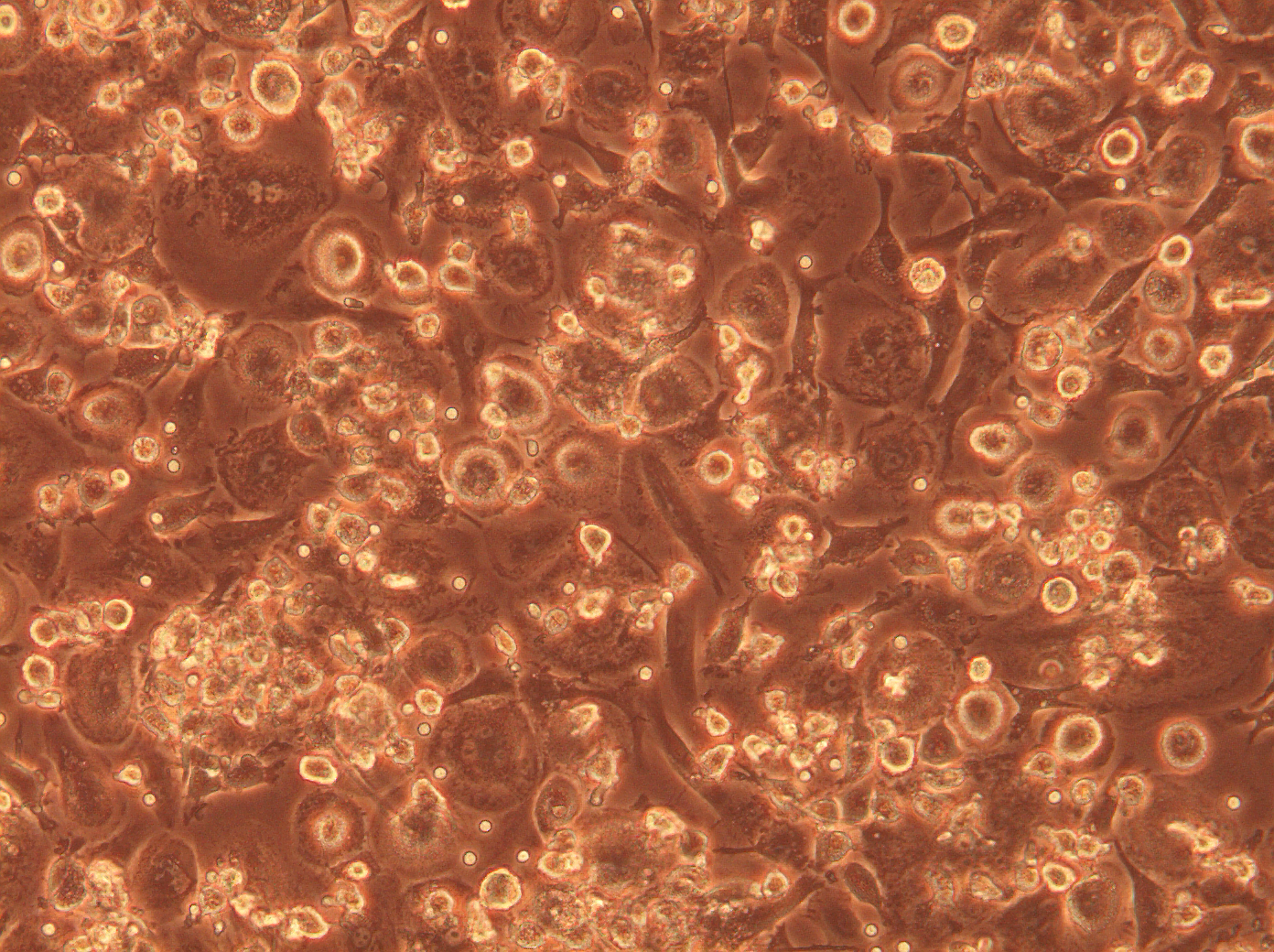

Supplement: Supplementary file 5 — Source Data for Expanded View [file EMMM-12-e10491-s012.zip › EV_source_data/Fig_EV1/Fig_EV1B_M2D.tif]

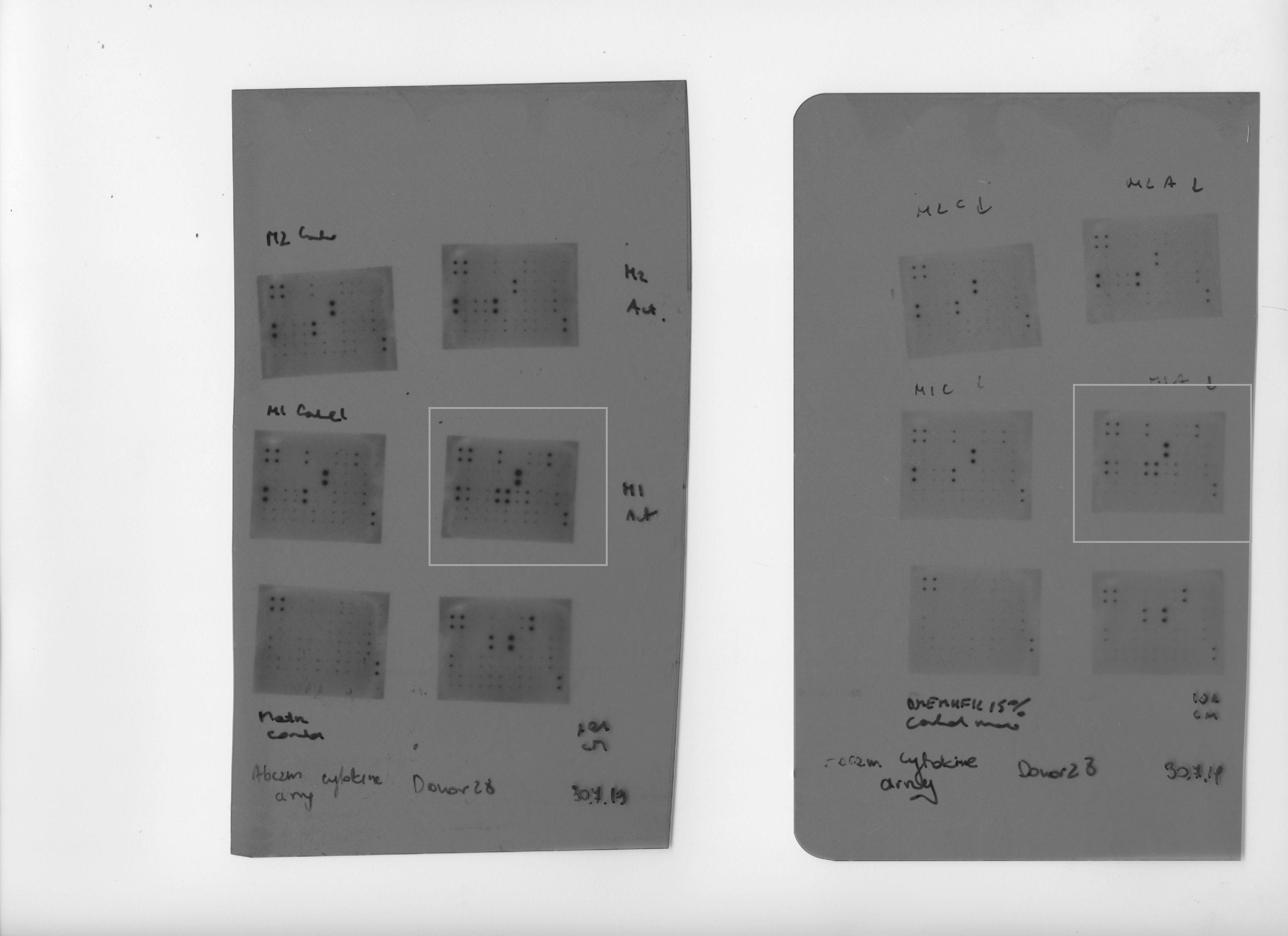

Supplement: Supplementary file 5 — Source Data for Expanded View [file EMMM-12-e10491-s012.zip › EV_source_data/Fig_EV1/Fig_EV1E.tif]

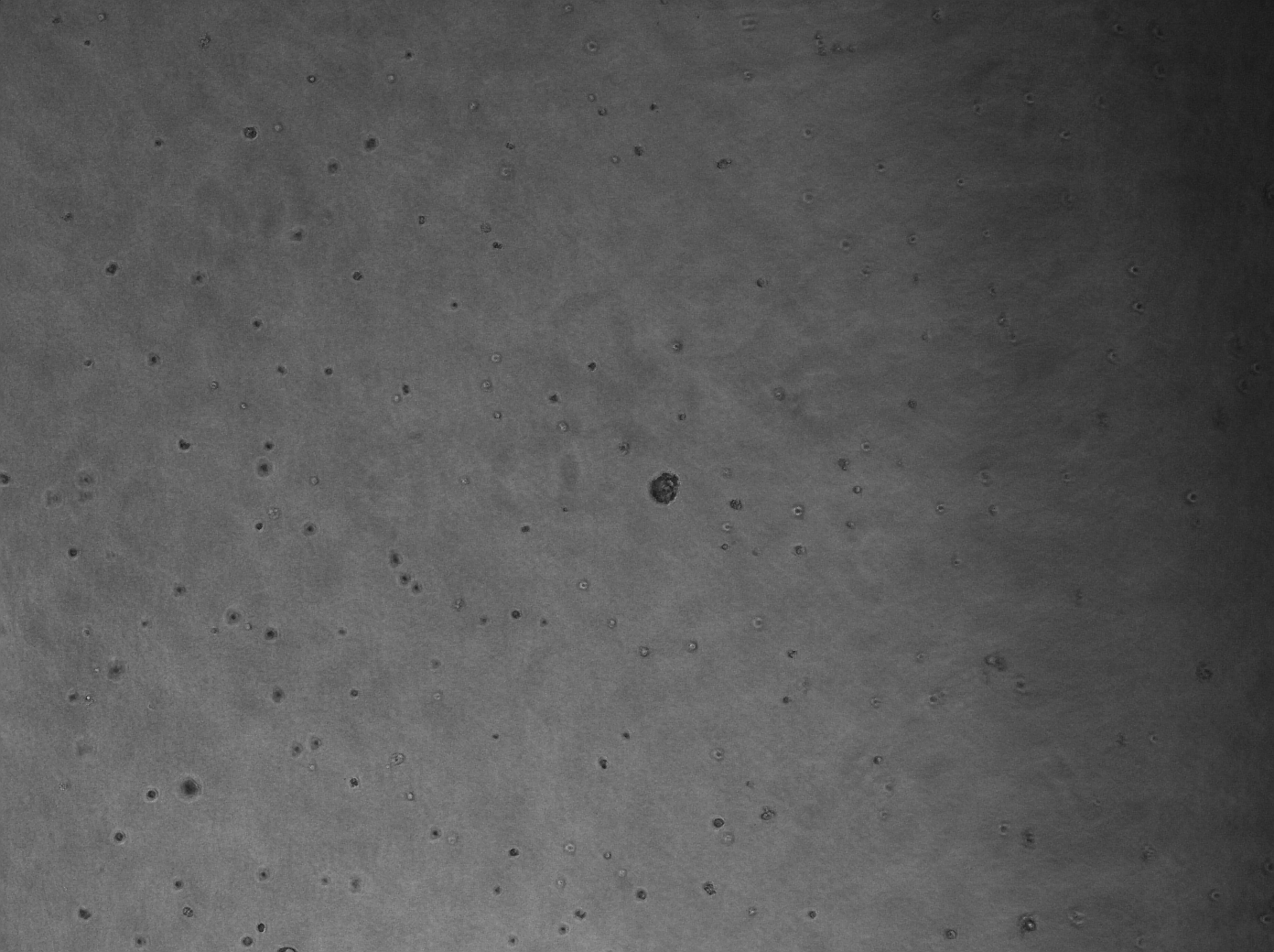

Supplement: Supplementary file 5 — Source Data for Expanded View [file EMMM-12-e10491-s012.zip › EV_source_data/Fig_EV3/Fig_EV3A_control.tif]

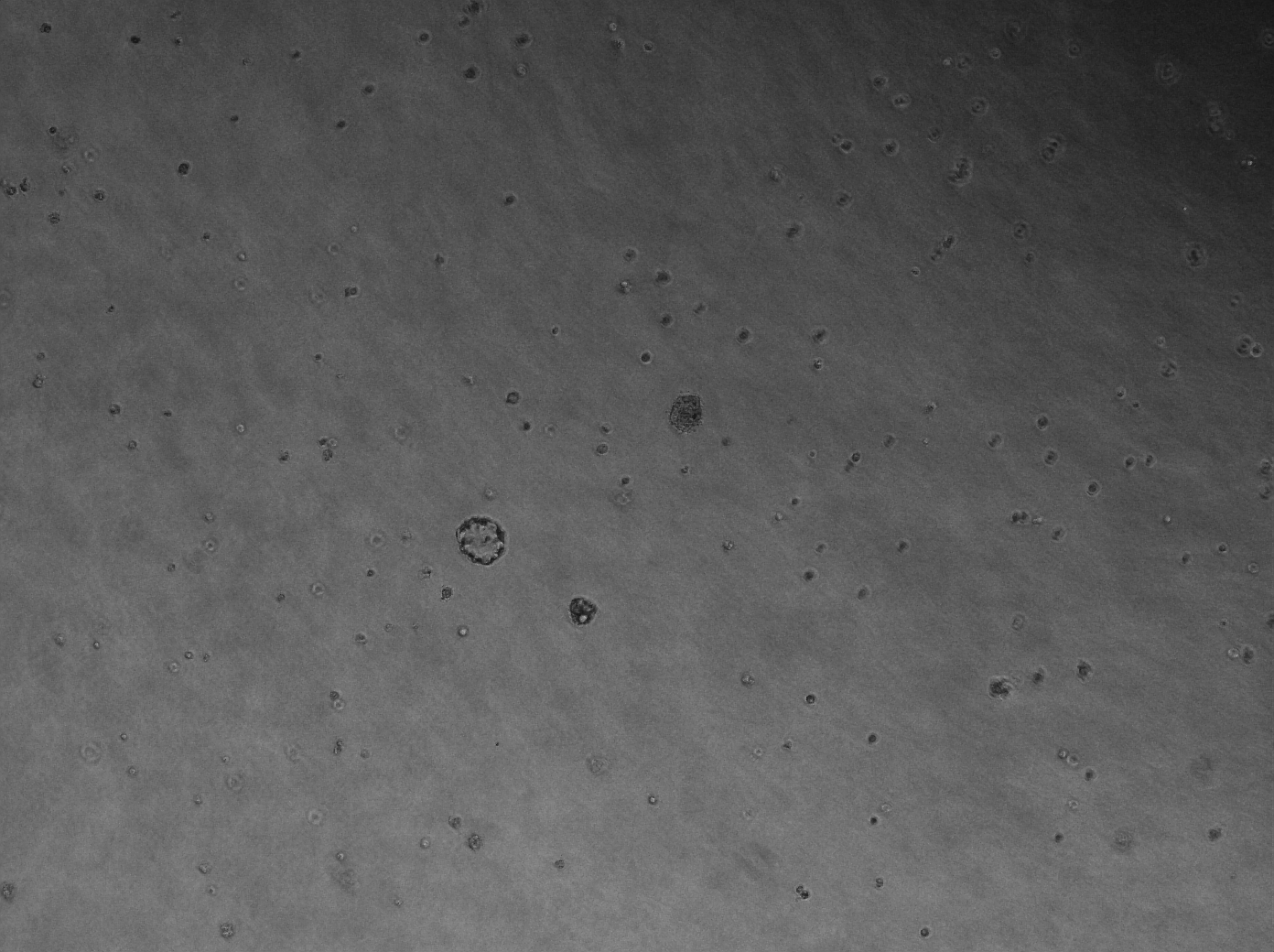

Supplement: Supplementary file 5 — Source Data for Expanded View [file EMMM-12-e10491-s012.zip › EV_source_data/Fig_EV3/Fig_EV3A_M1A.tif]

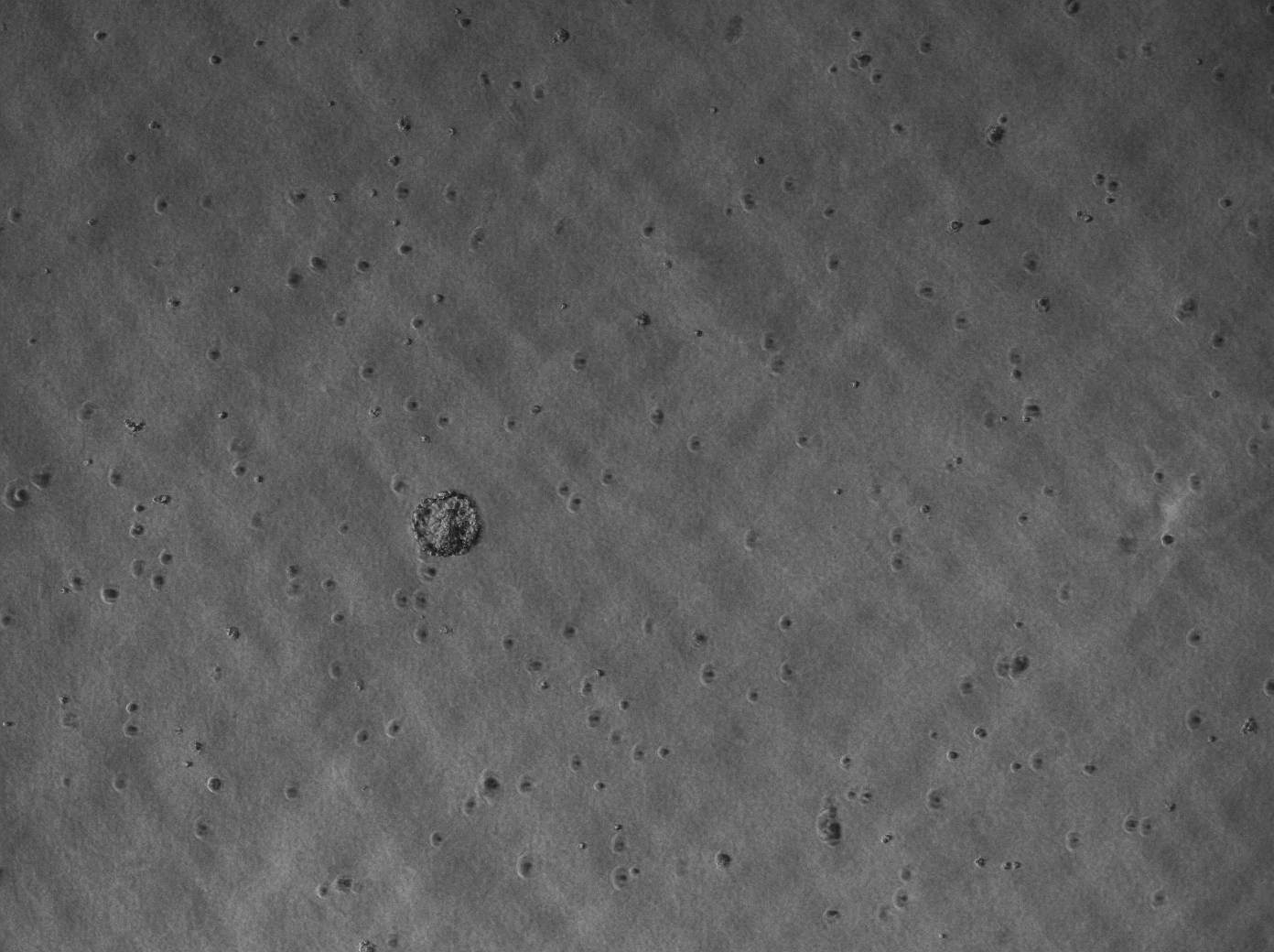

Supplement: Supplementary file 5 — Source Data for Expanded View [file EMMM-12-e10491-s012.zip › EV_source_data/Fig_EV3/Fig_EV3A_M1D.tif]

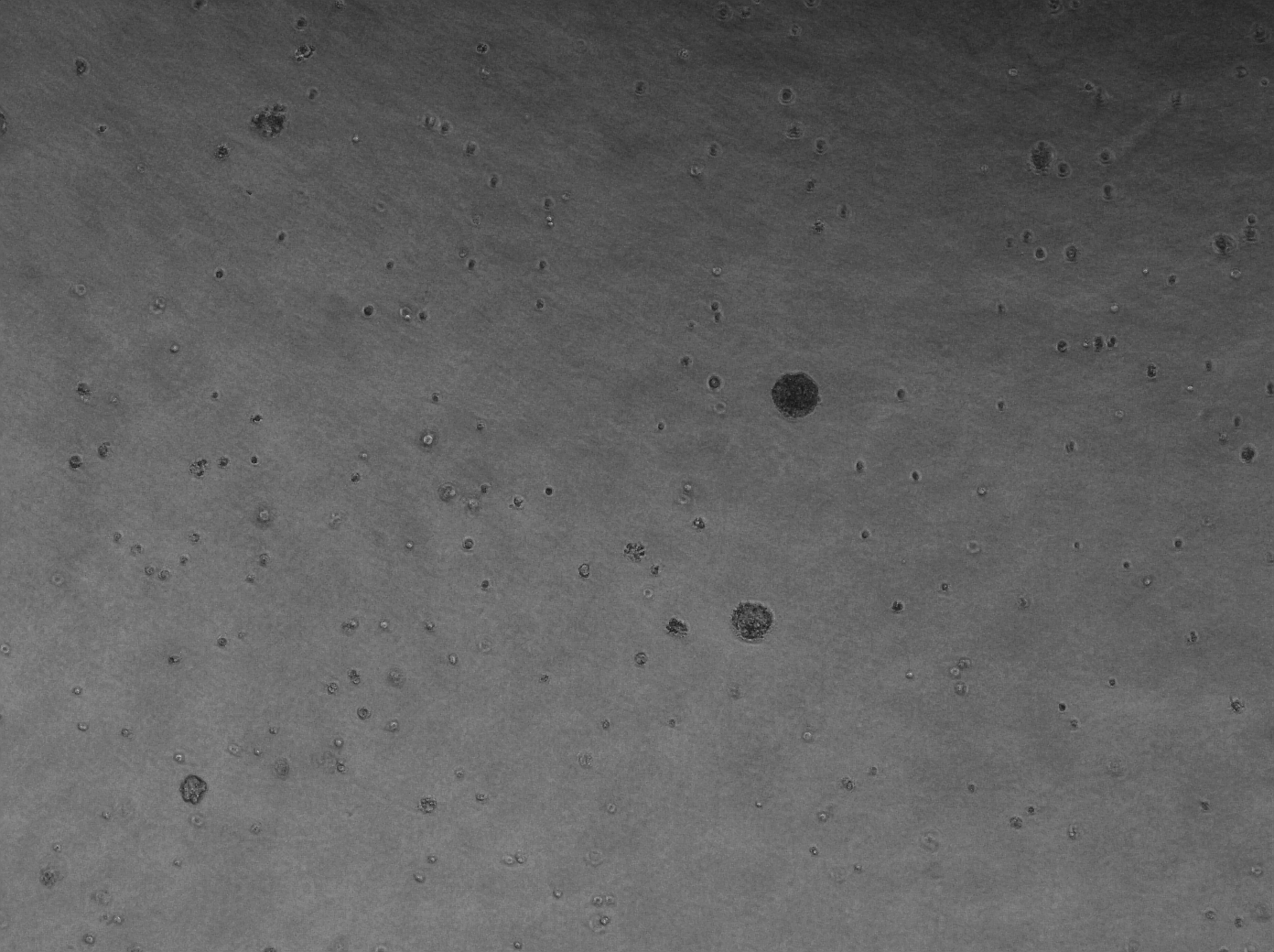

Supplement: Supplementary file 5 — Source Data for Expanded View [file EMMM-12-e10491-s012.zip › EV_source_data/Fig_EV3/Fig_EV3A_M2A.tif]

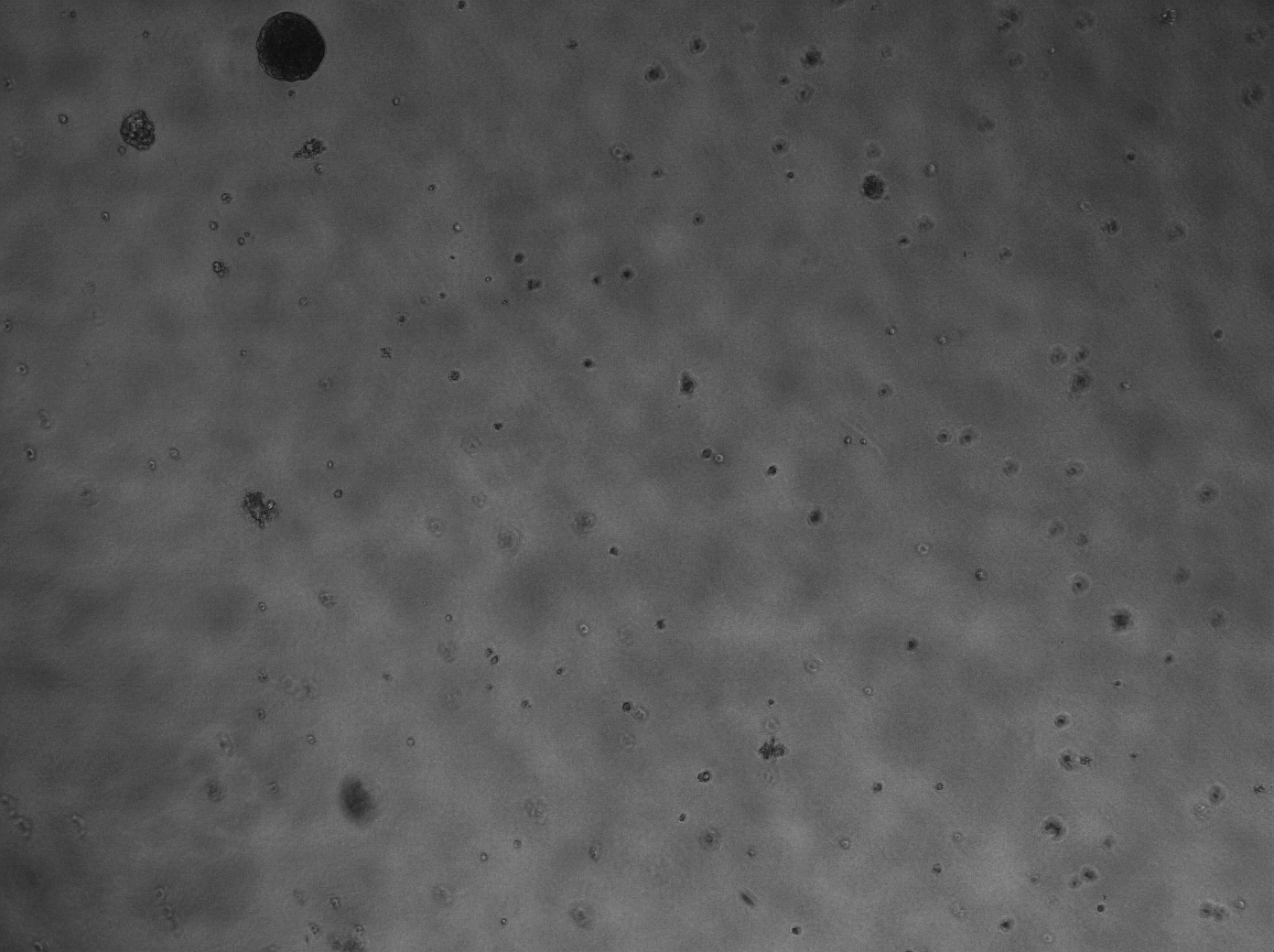

Supplement: Supplementary file 5 — Source Data for Expanded View [file EMMM-12-e10491-s012.zip › EV_source_data/Fig_EV3/Fig_EV3A_M2D.tif]

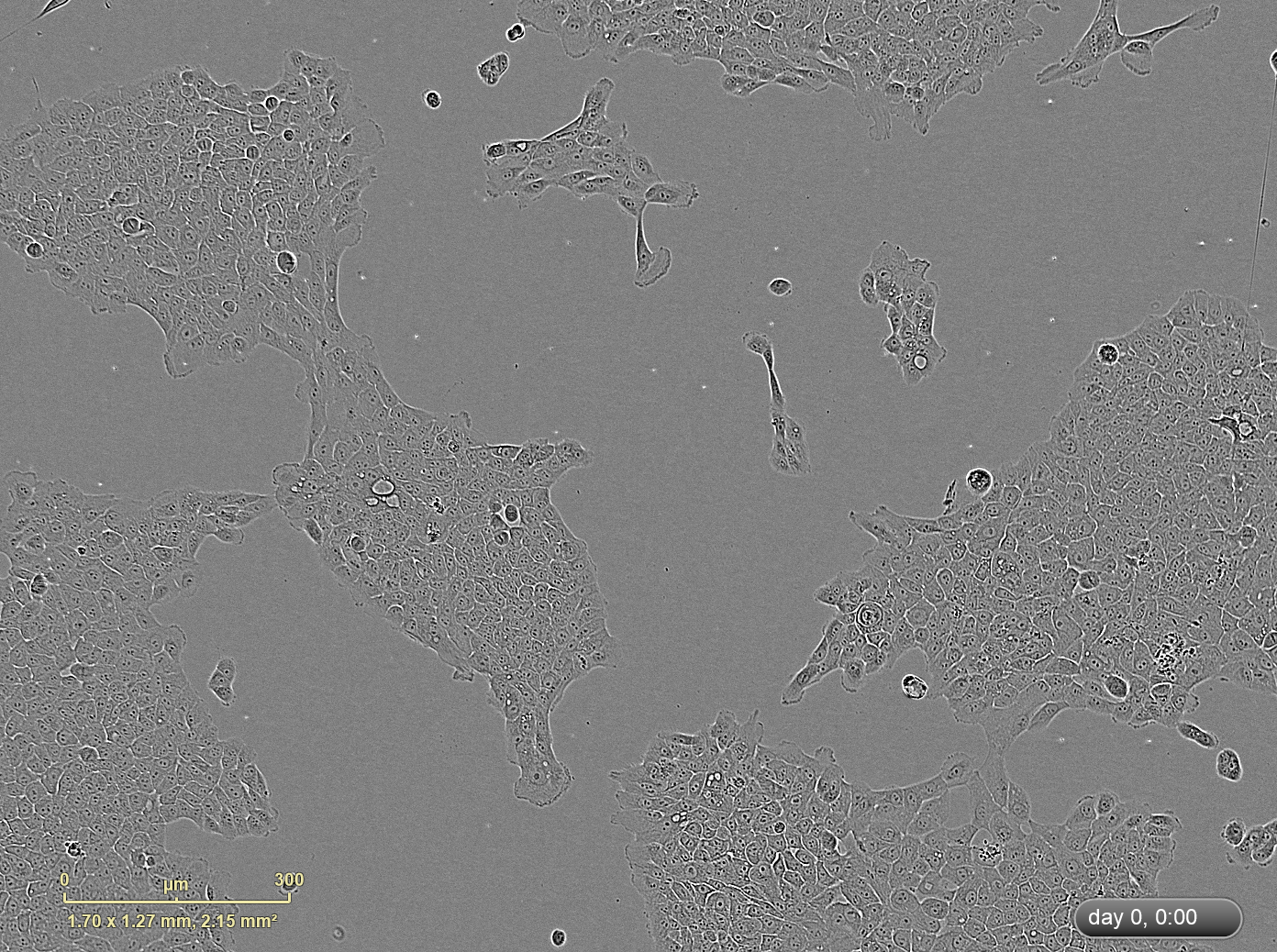

Supplement: Supplementary file 5 — Source Data for Expanded View [file EMMM-12-e10491-s012.zip › EV_source_data/Fig_EV3/Fig_EV3C_control.tif]

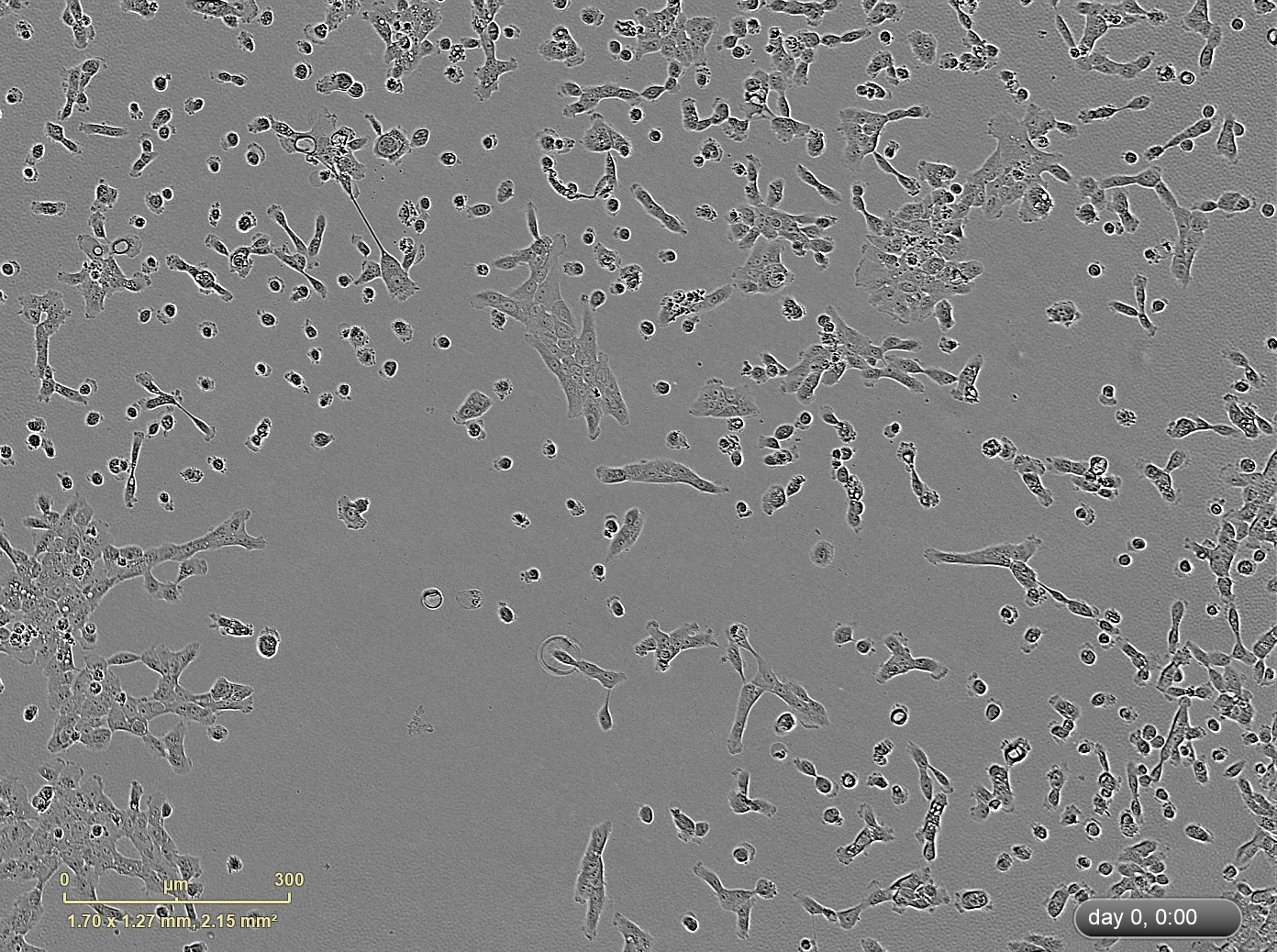

Supplement: Supplementary file 5 — Source Data for Expanded View [file EMMM-12-e10491-s012.zip › EV_source_data/Fig_EV3/Fig_EV3C_M1A.tif]

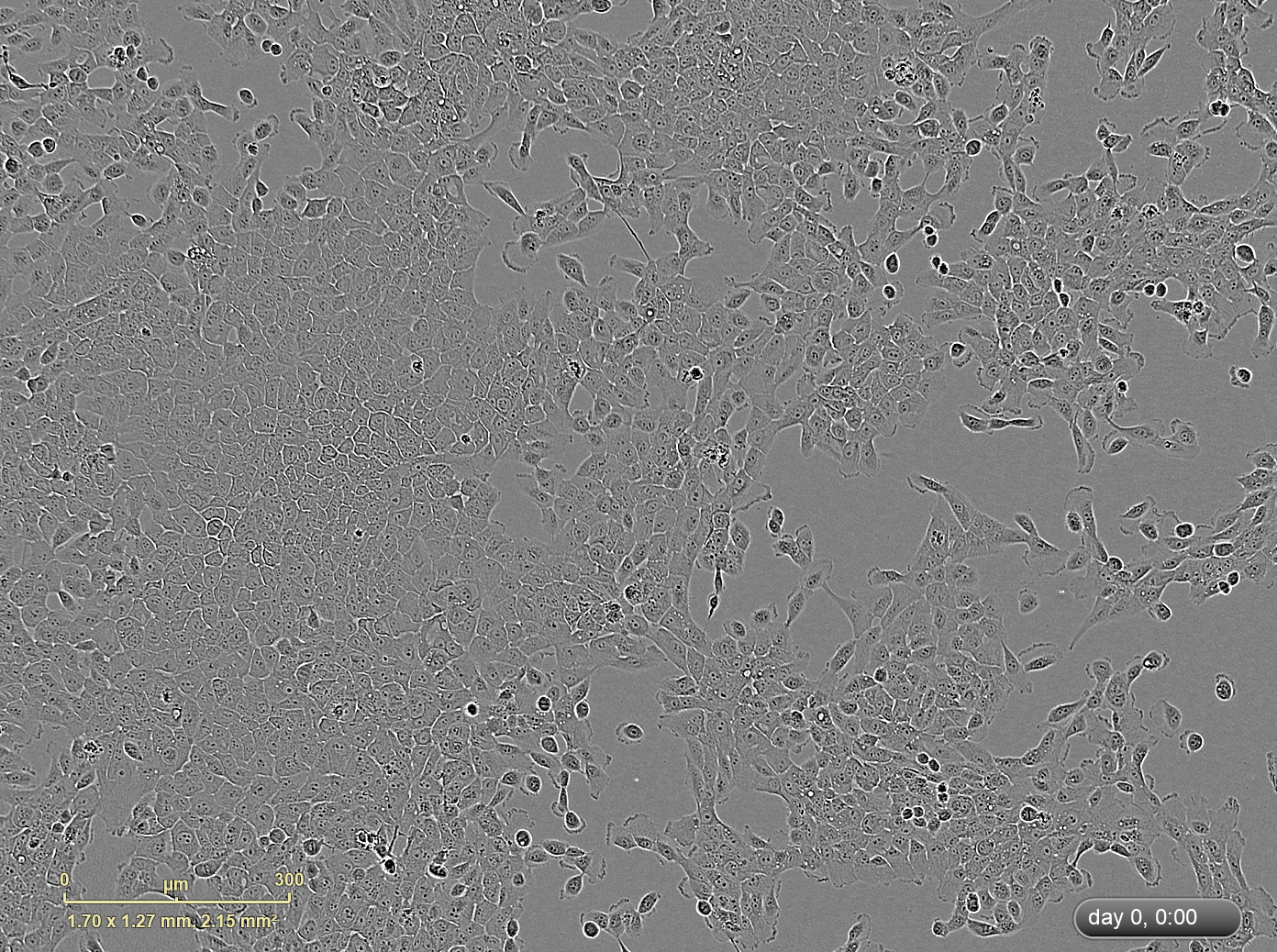

Supplement: Supplementary file 5 — Source Data for Expanded View [file EMMM-12-e10491-s012.zip › EV_source_data/Fig_EV3/Fig_EV3C_M1D.tif]

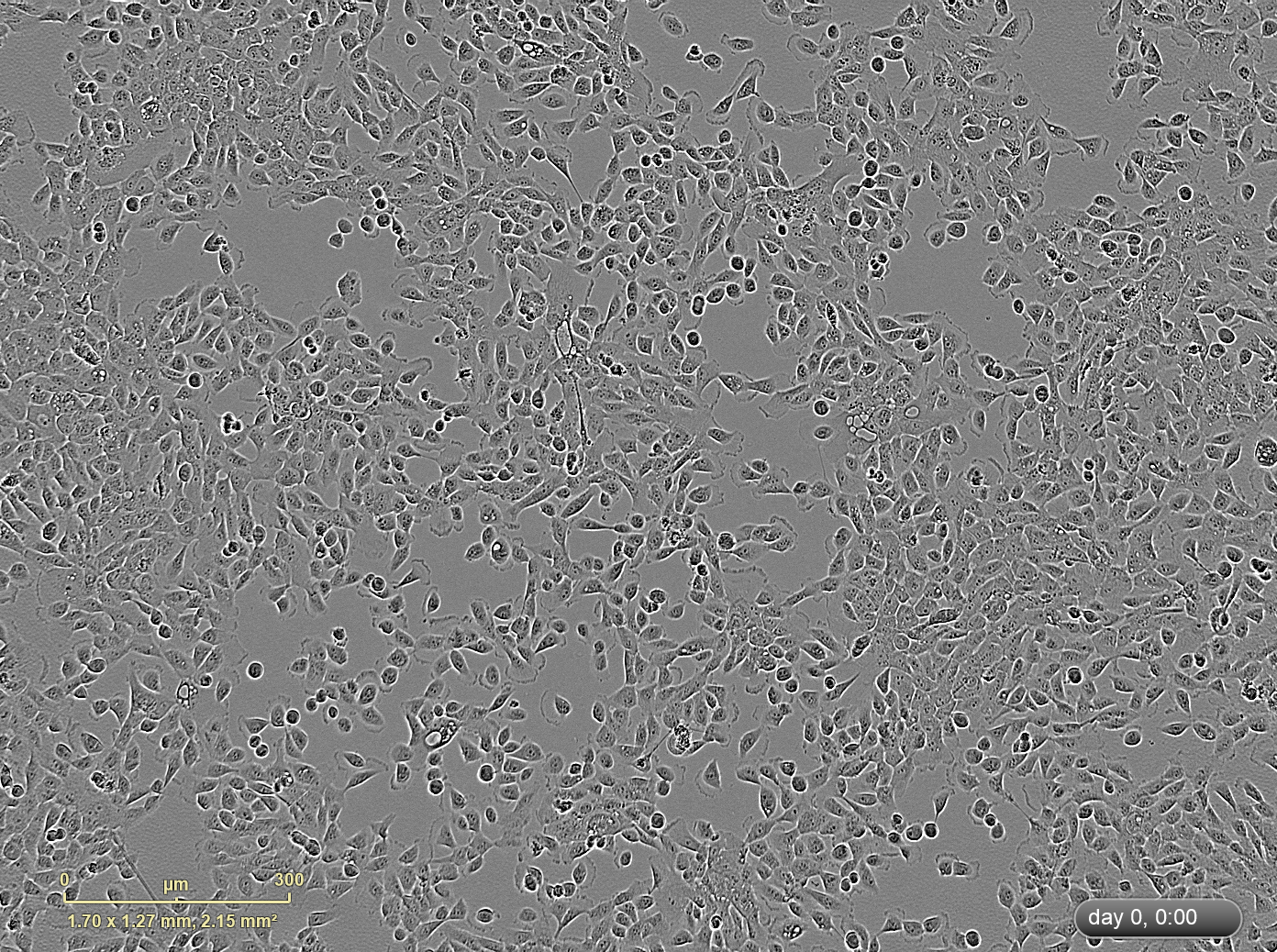

Supplement: Supplementary file 5 — Source Data for Expanded View [file EMMM-12-e10491-s012.zip › EV_source_data/Fig_EV3/Fig_EV3C_M2A.tif]

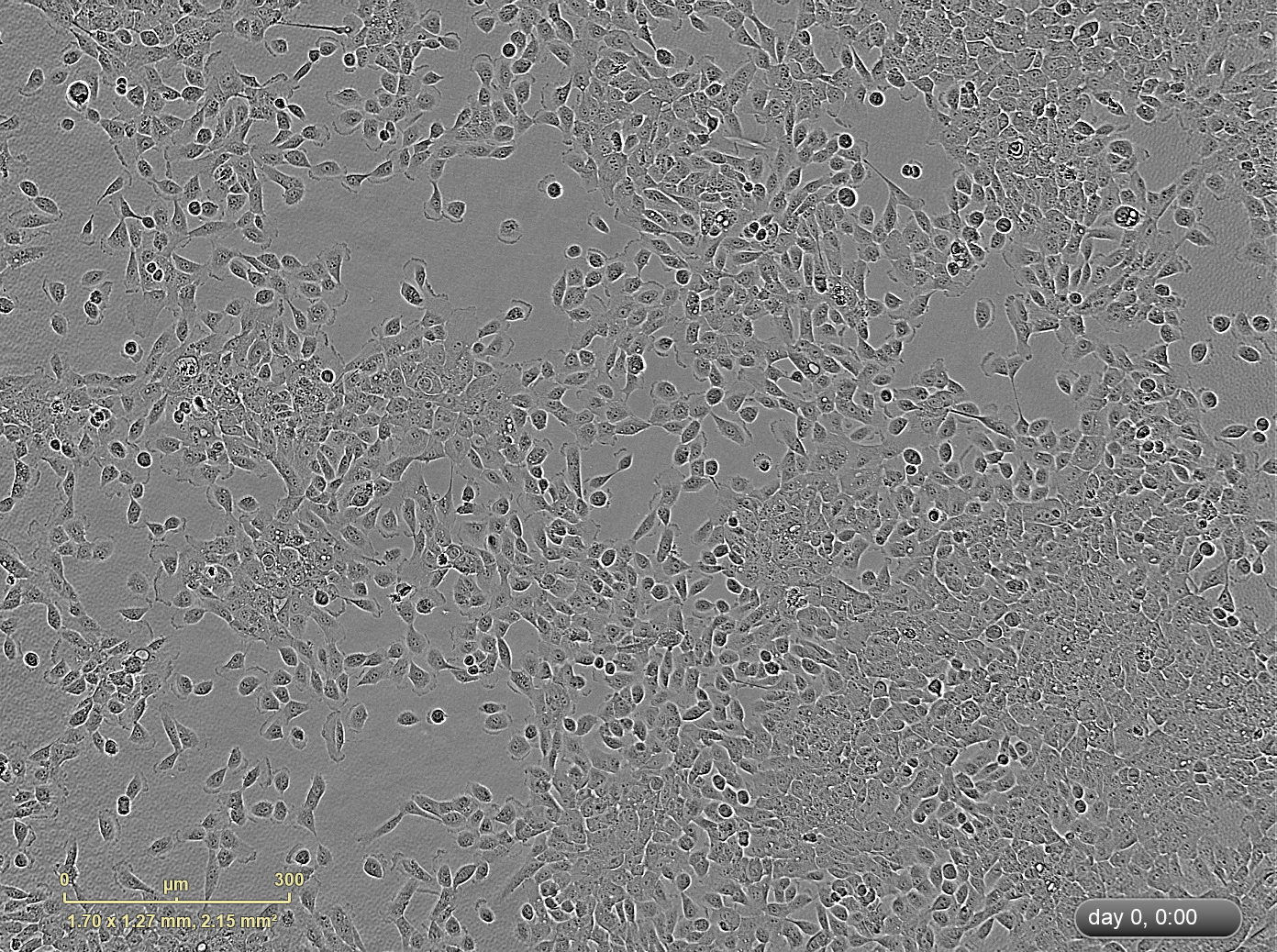

Supplement: Supplementary file 5 — Source Data for Expanded View [file EMMM-12-e10491-s012.zip › EV_source_data/Fig_EV3/Fig_EV3C_M2D.tif]

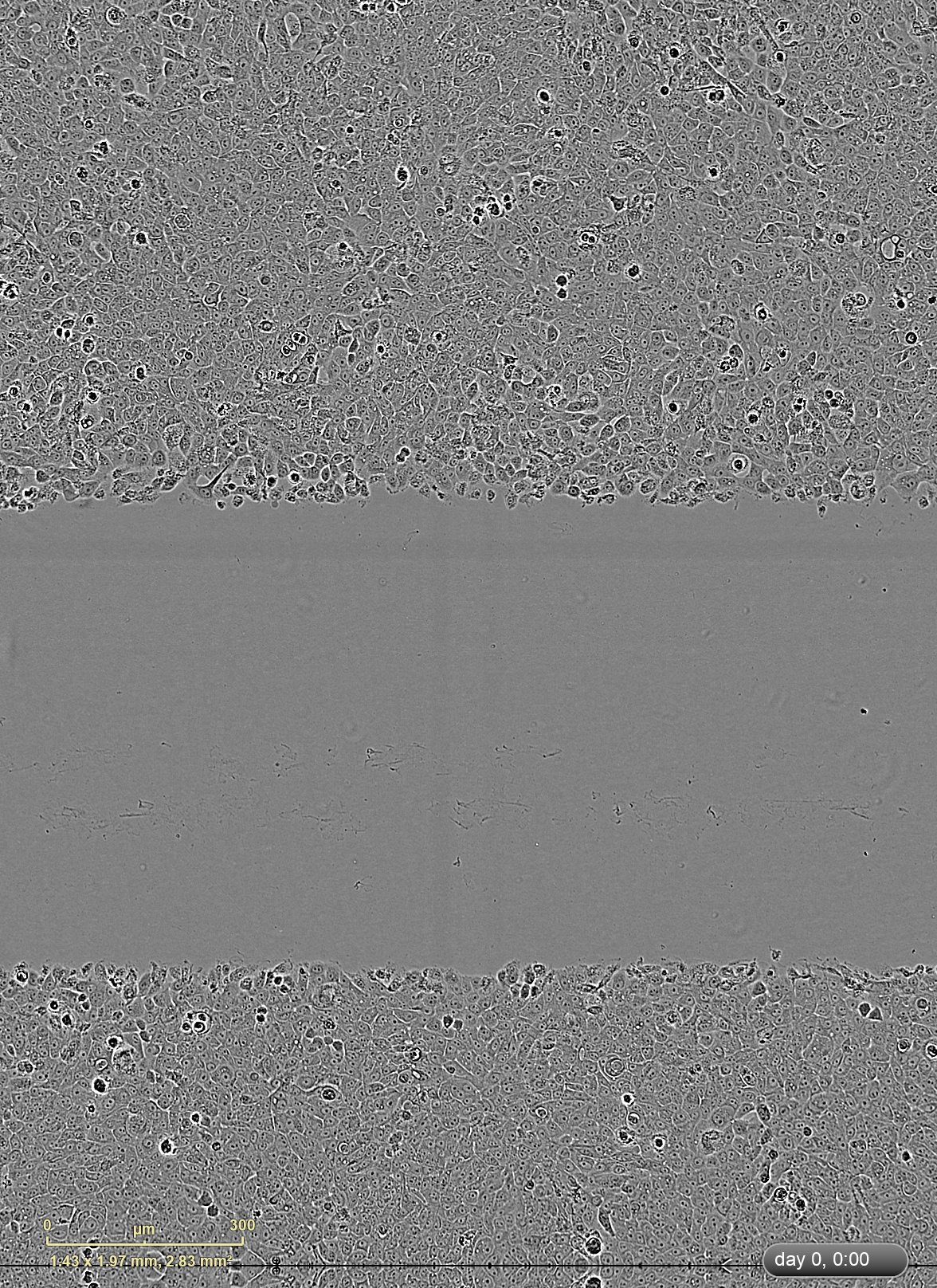

Supplement: Supplementary file 5 — Source Data for Expanded View [file EMMM-12-e10491-s012.zip › EV_source_data/Fig_EV3/Fig_EV3H_control_(0h).tif]

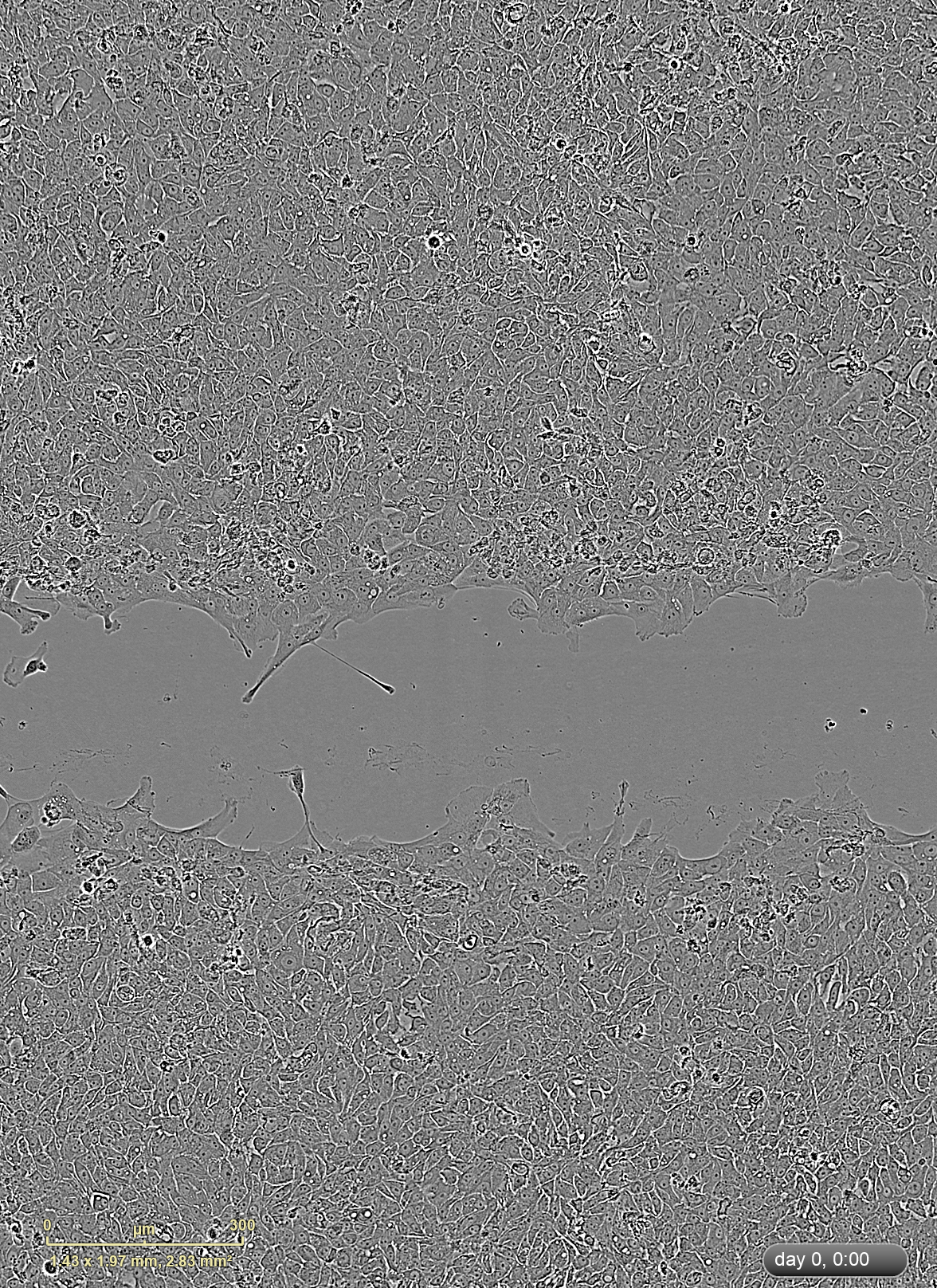

Supplement: Supplementary file 5 — Source Data for Expanded View [file EMMM-12-e10491-s012.zip › EV_source_data/Fig_EV3/Fig_EV3H_control_(48h).tif]

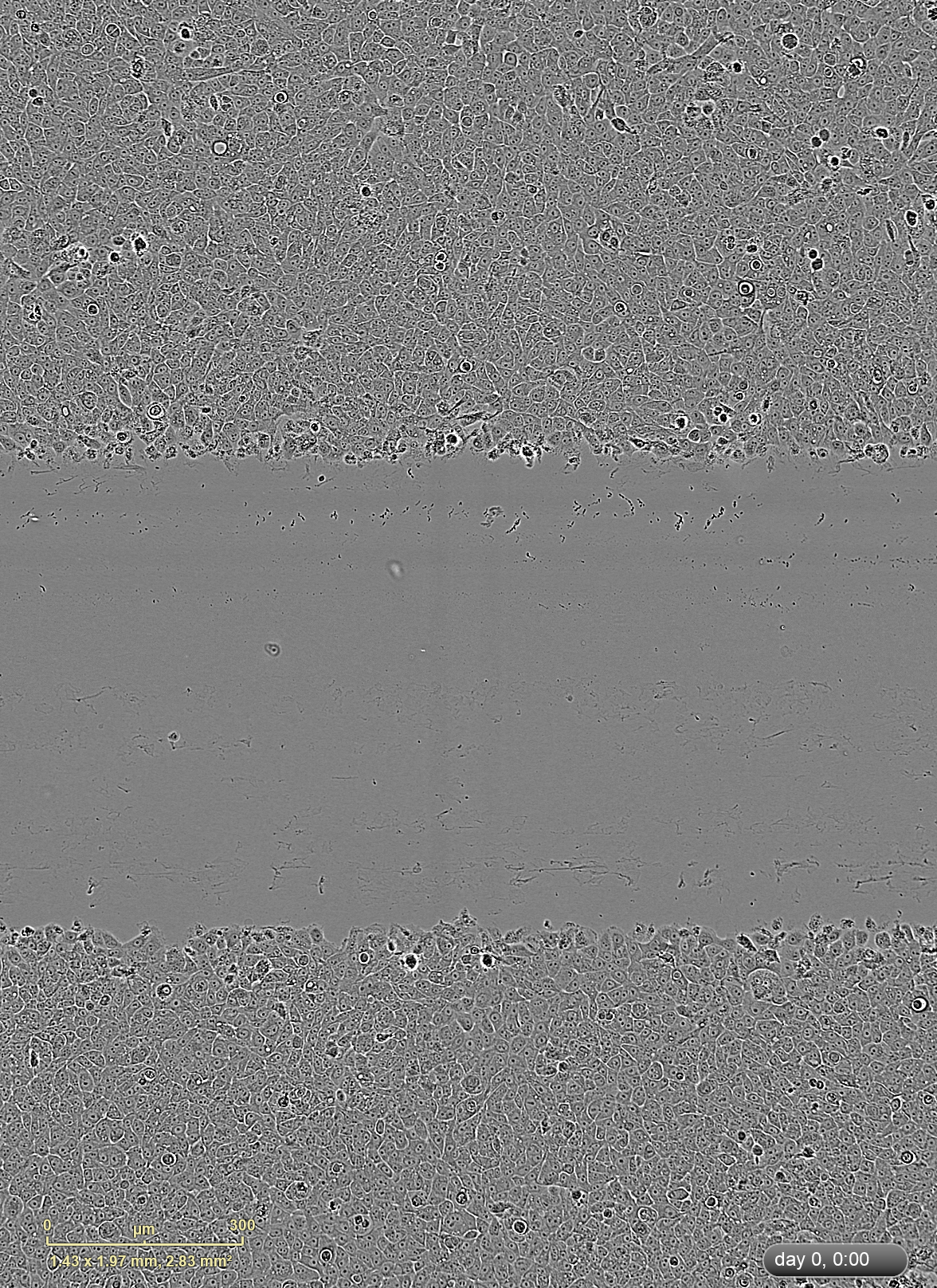

Supplement: Supplementary file 5 — Source Data for Expanded View [file EMMM-12-e10491-s012.zip › EV_source_data/Fig_EV3/Fig_EV3H_M1A(0h).tif]

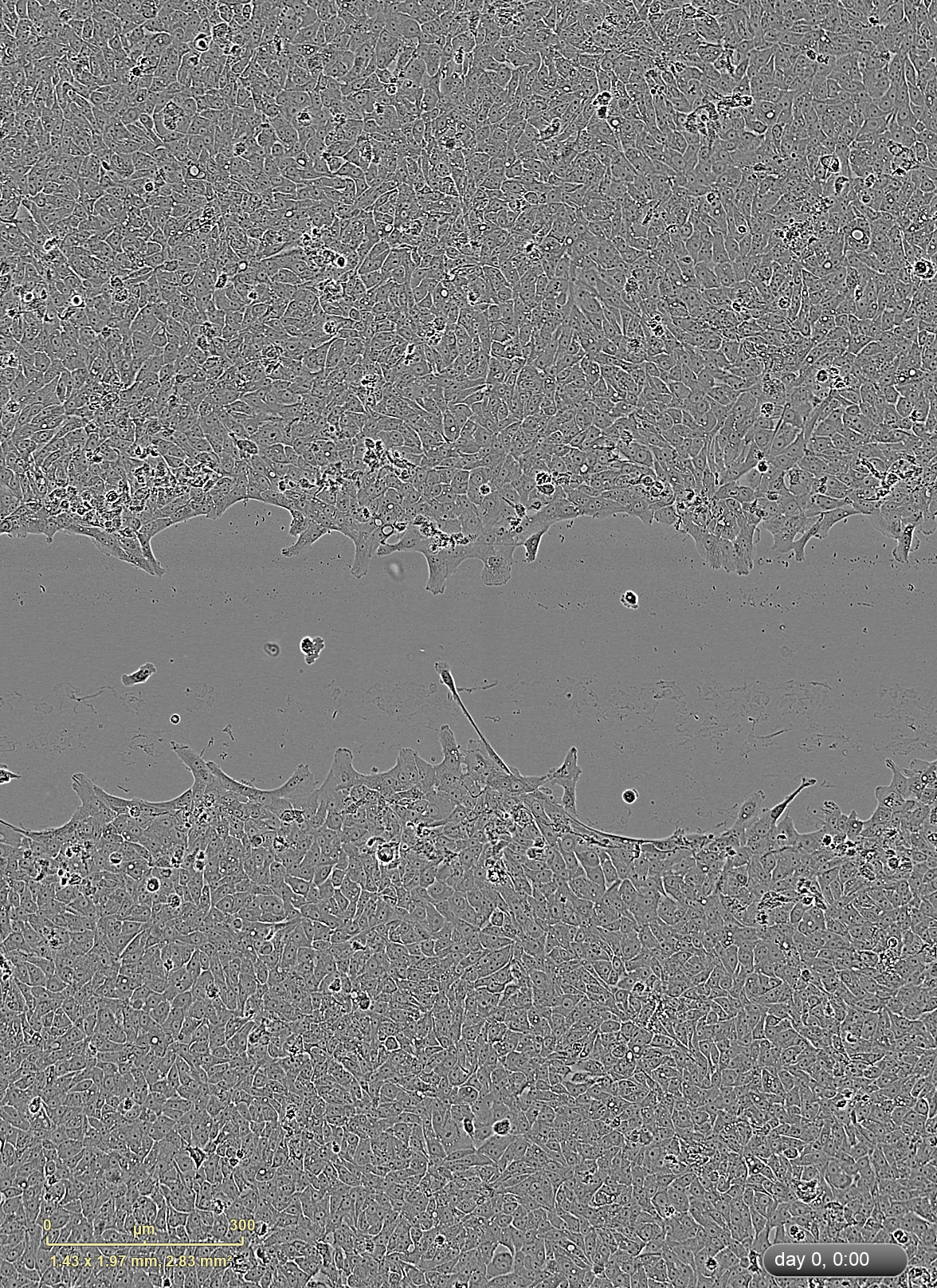

Supplement: Supplementary file 5 — Source Data for Expanded View [file EMMM-12-e10491-s012.zip › EV_source_data/Fig_EV3/Fig_EV3H_M1A(48h).tif]

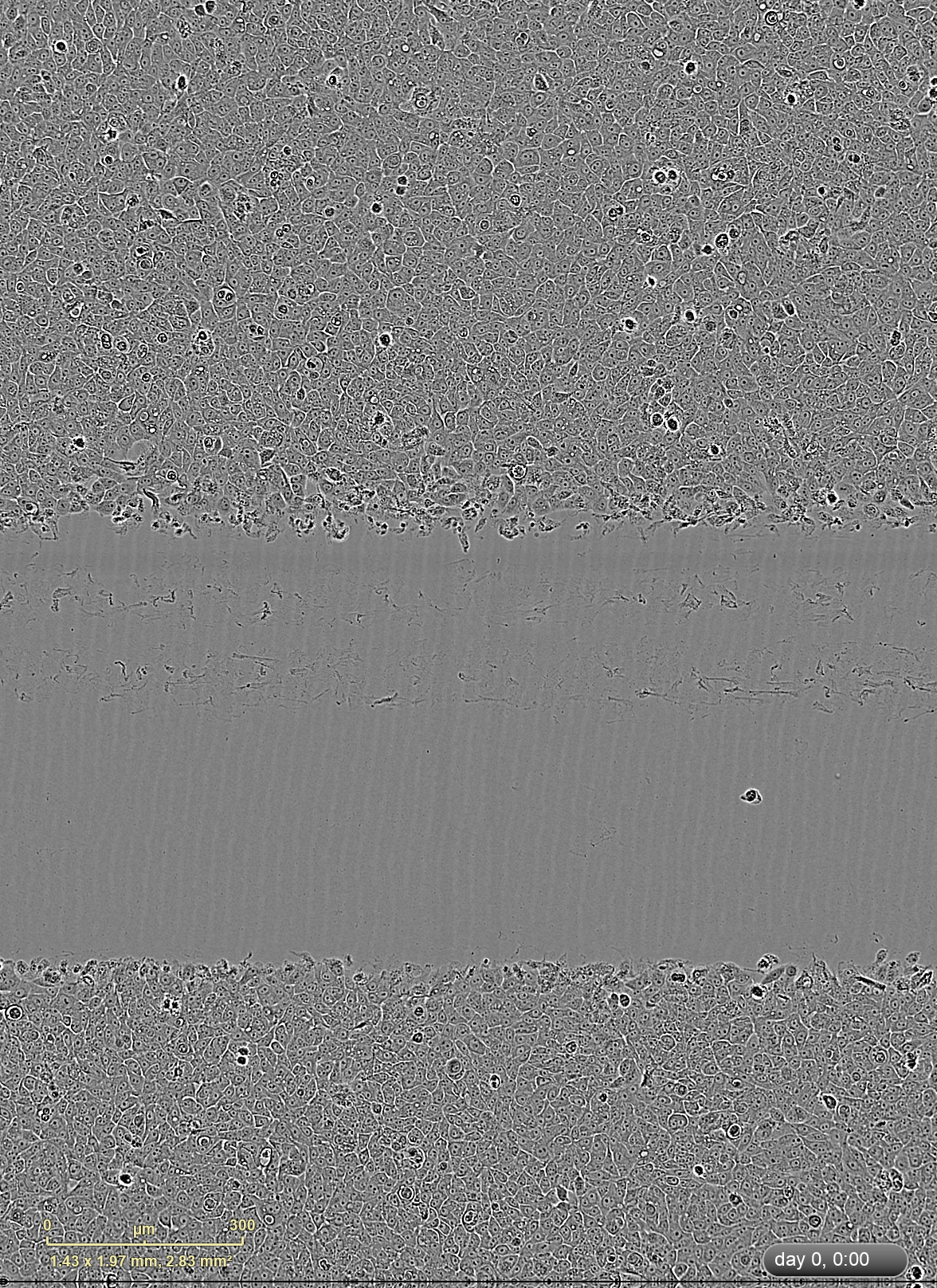

Supplement: Supplementary file 5 — Source Data for Expanded View [file EMMM-12-e10491-s012.zip › EV_source_data/Fig_EV3/Fig_EV3H_M1D_(0h).tif]

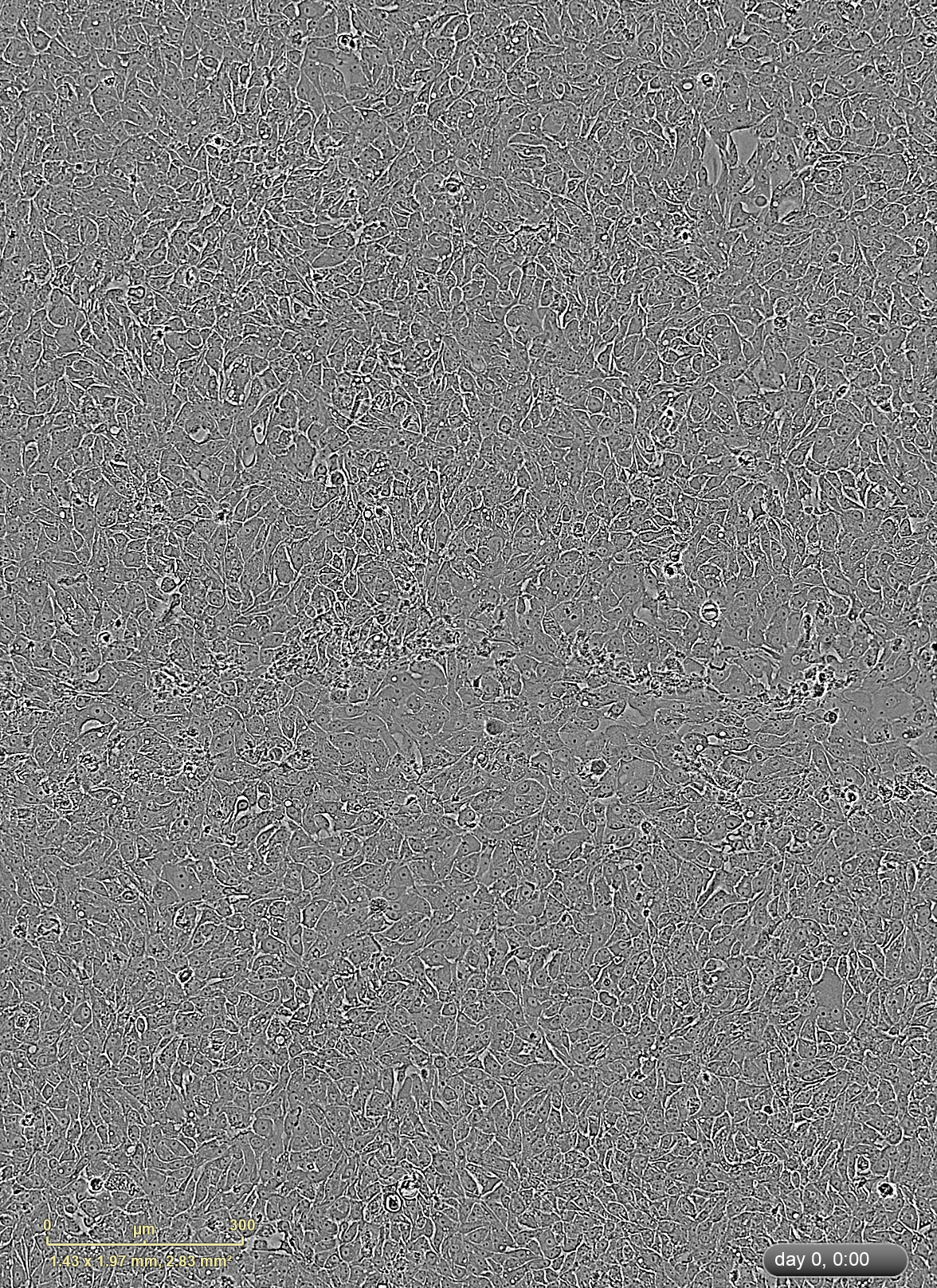

Supplement: Supplementary file 5 — Source Data for Expanded View [file EMMM-12-e10491-s012.zip › EV_source_data/Fig_EV3/Fig_EV3H_M1D_(48h).tif]

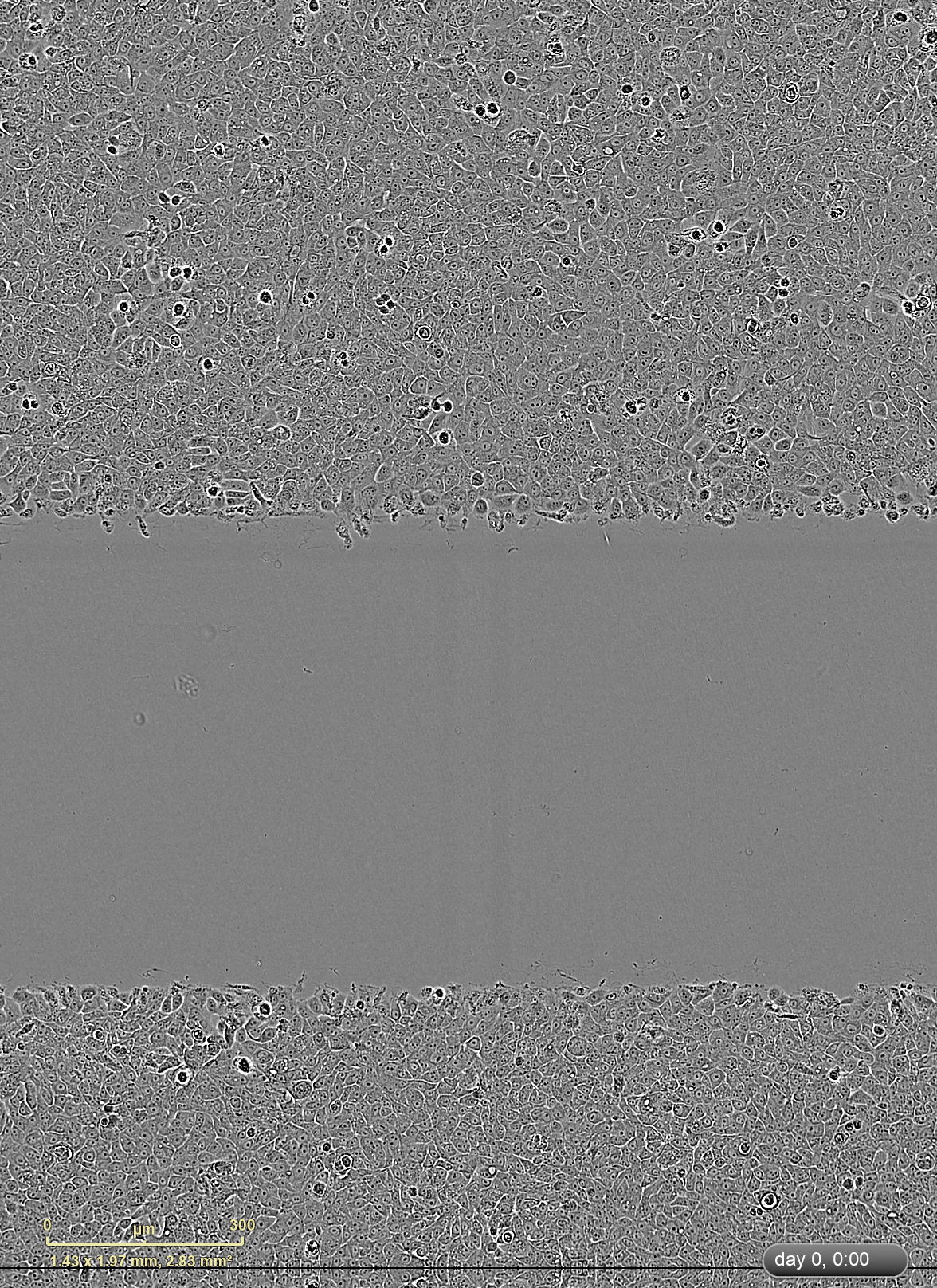

Supplement: Supplementary file 5 — Source Data for Expanded View [file EMMM-12-e10491-s012.zip › EV_source_data/Fig_EV3/Fig_EV3H_M2A(0h).tif]

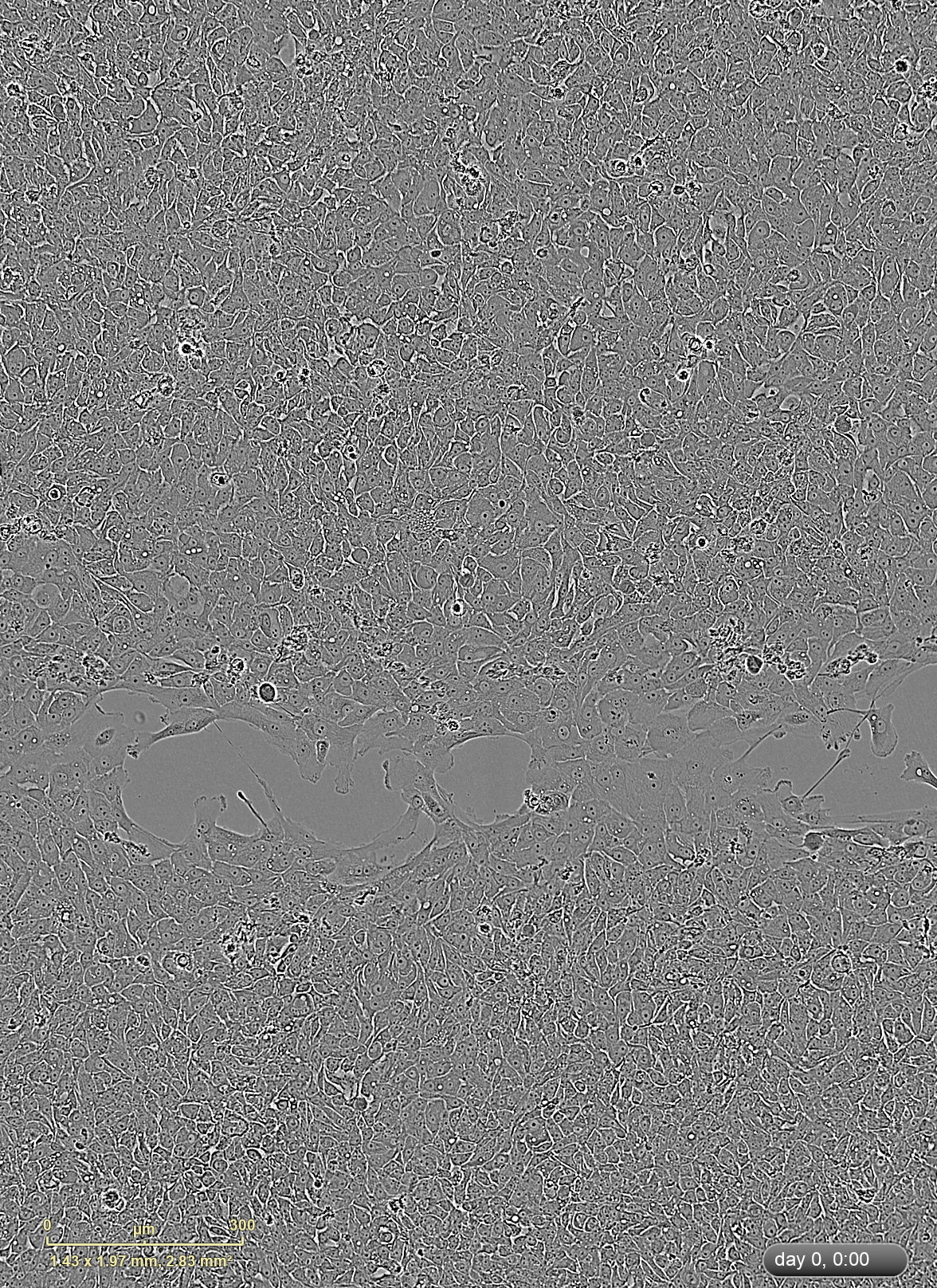

Supplement: Supplementary file 5 — Source Data for Expanded View [file EMMM-12-e10491-s012.zip › EV_source_data/Fig_EV3/Fig_EV3H_M2A(48h).tif]

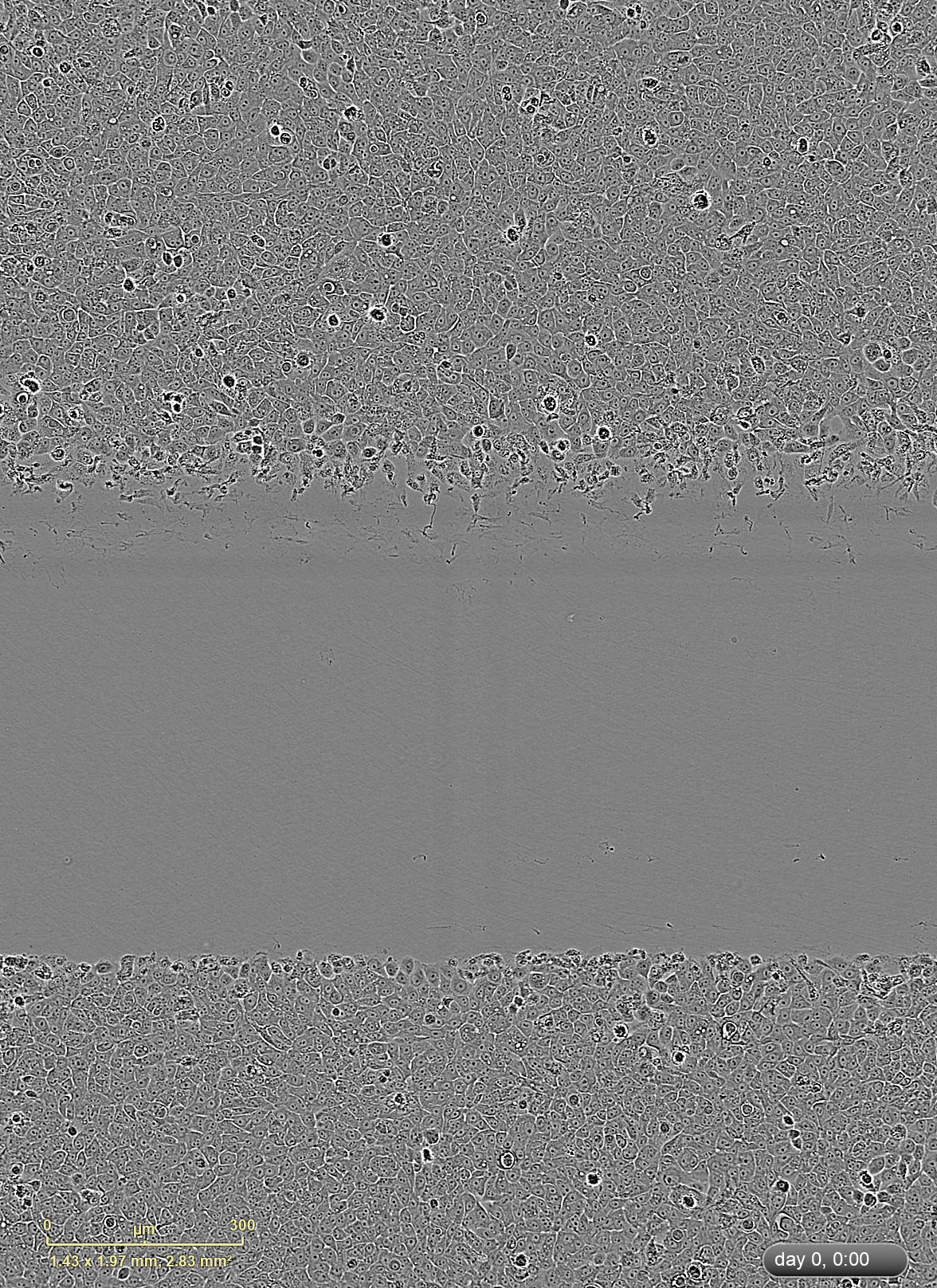

Supplement: Supplementary file 5 — Source Data for Expanded View [file EMMM-12-e10491-s012.zip › EV_source_data/Fig_EV3/Fig_EV3H_M2D_(0h).tif]

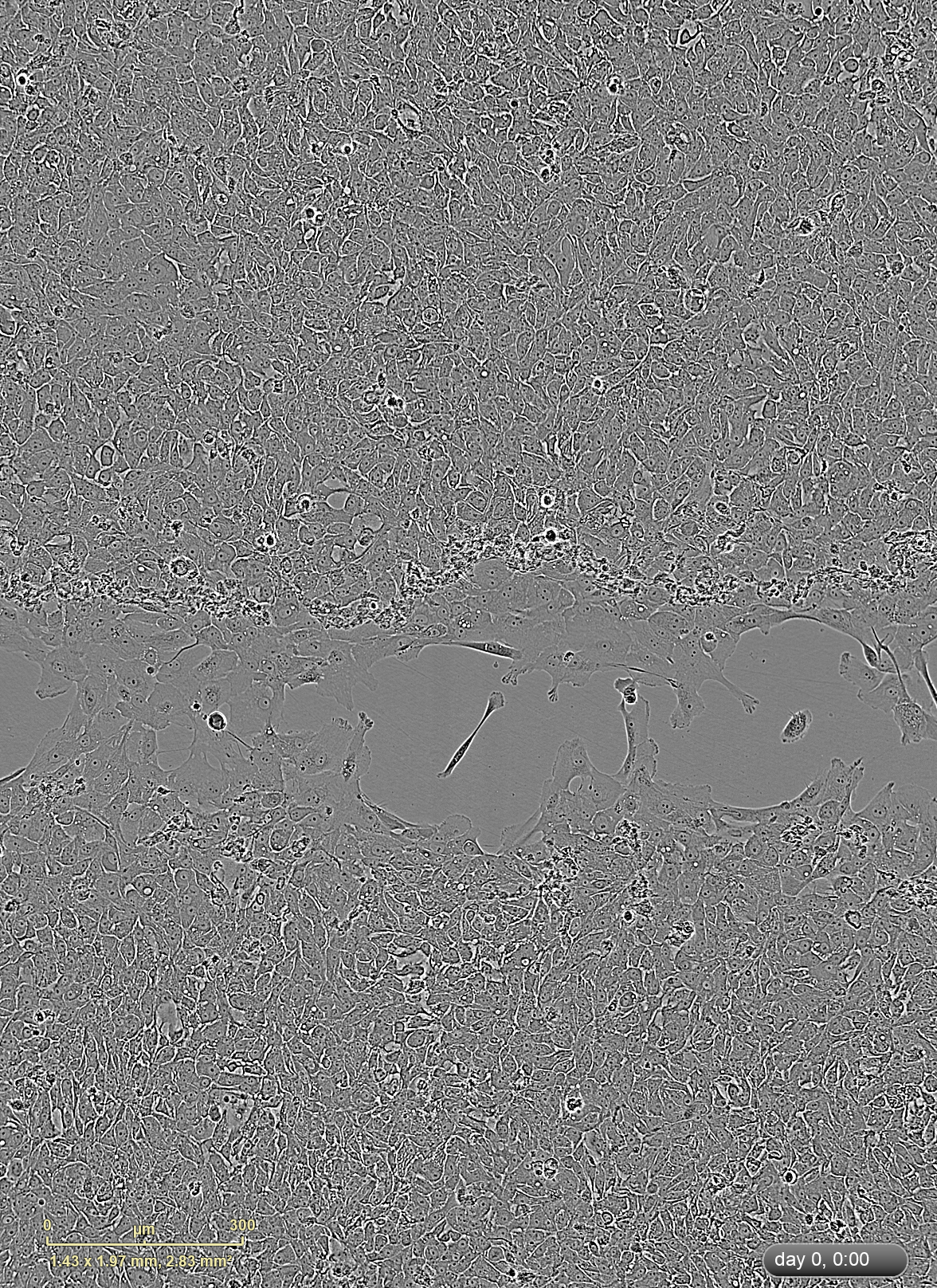

Supplement: Supplementary file 5 — Source Data for Expanded View [file EMMM-12-e10491-s012.zip › EV_source_data/Fig_EV3/Fig_EV3H_M2D_(48h).tif]

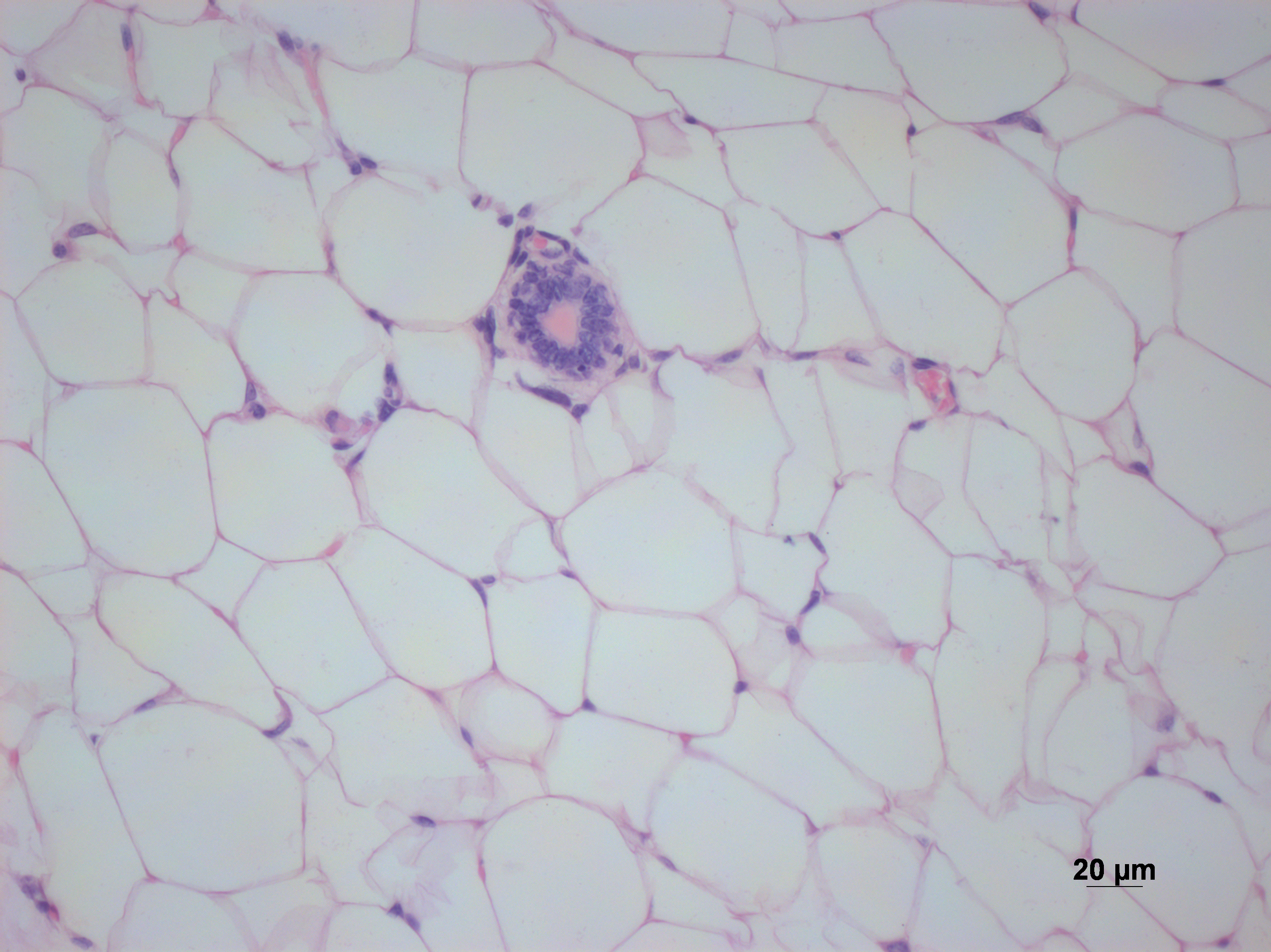

Supplement: Supplementary file 5 — Source Data for Expanded View [file EMMM-12-e10491-s012.zip › EV_source_data/Fig_EV4/Fig_EV4C_HFD.tif]

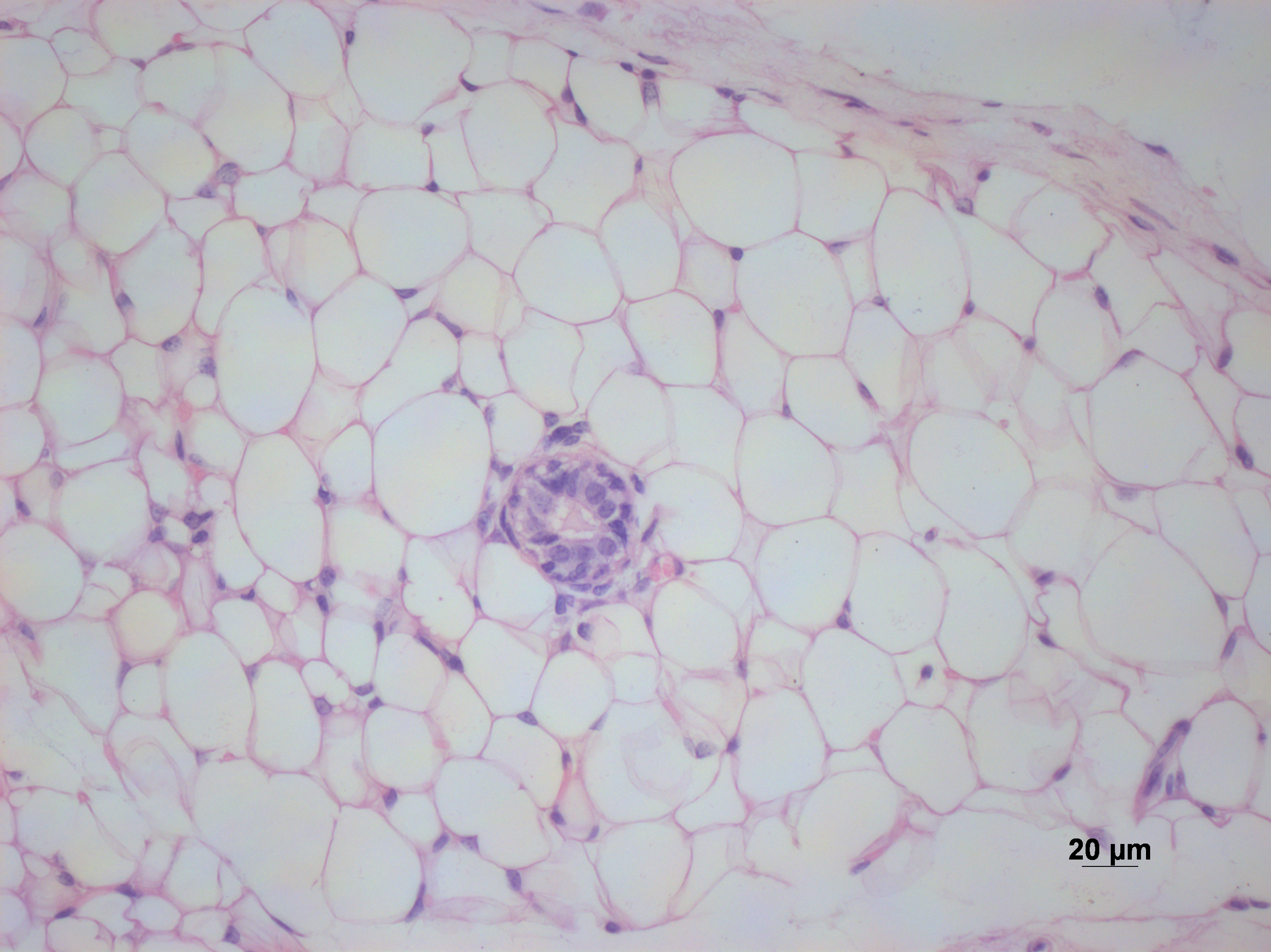

Supplement: Supplementary file 5 — Source Data for Expanded View [file EMMM-12-e10491-s012.zip › EV_source_data/Fig_EV4/Fig_EV4C_ND.tif]

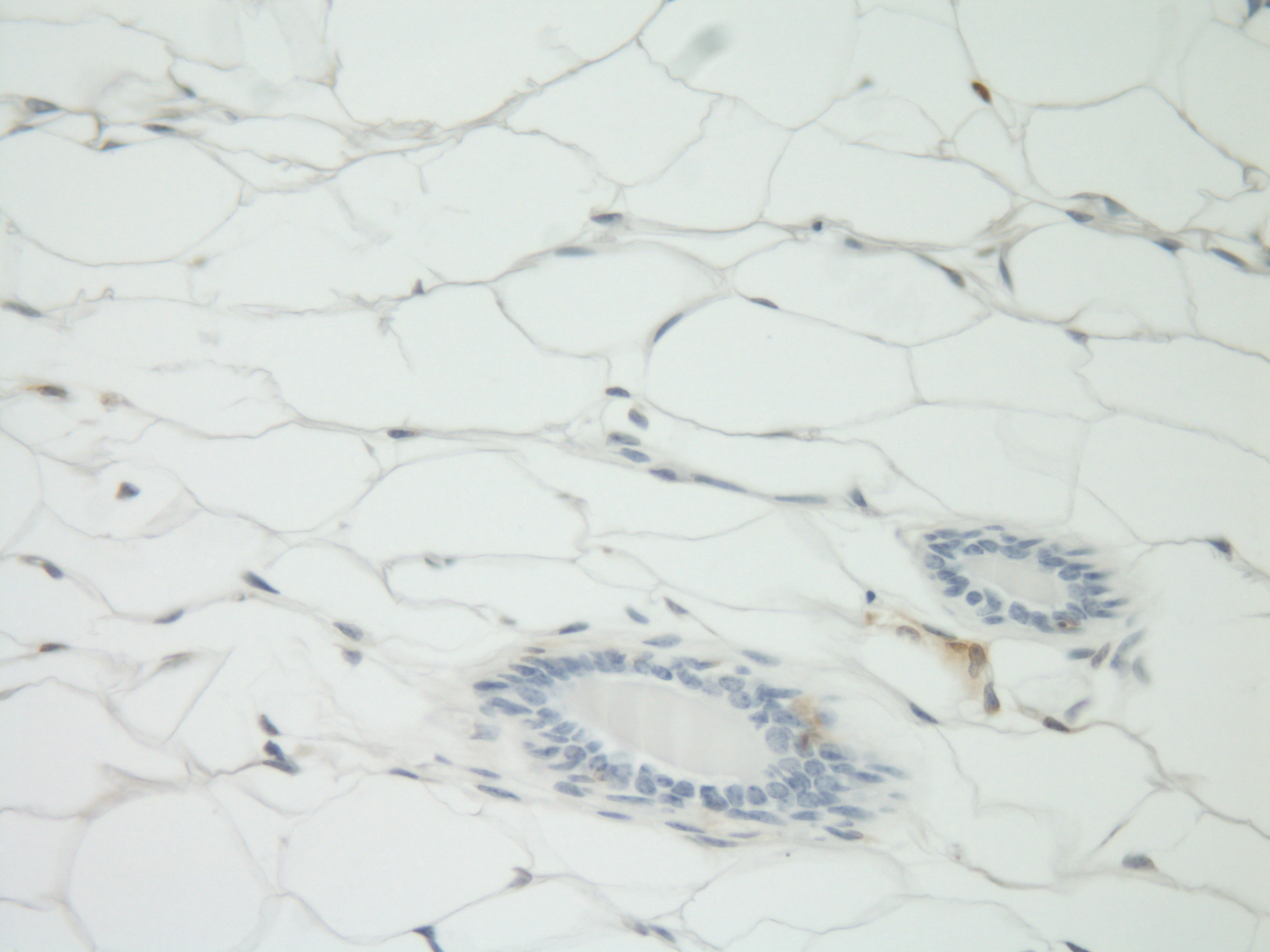

Supplement: Supplementary file 5 — Source Data for Expanded View [file EMMM-12-e10491-s012.zip › EV_source_data/Fig_EV4/Fig_EV4E.tif]

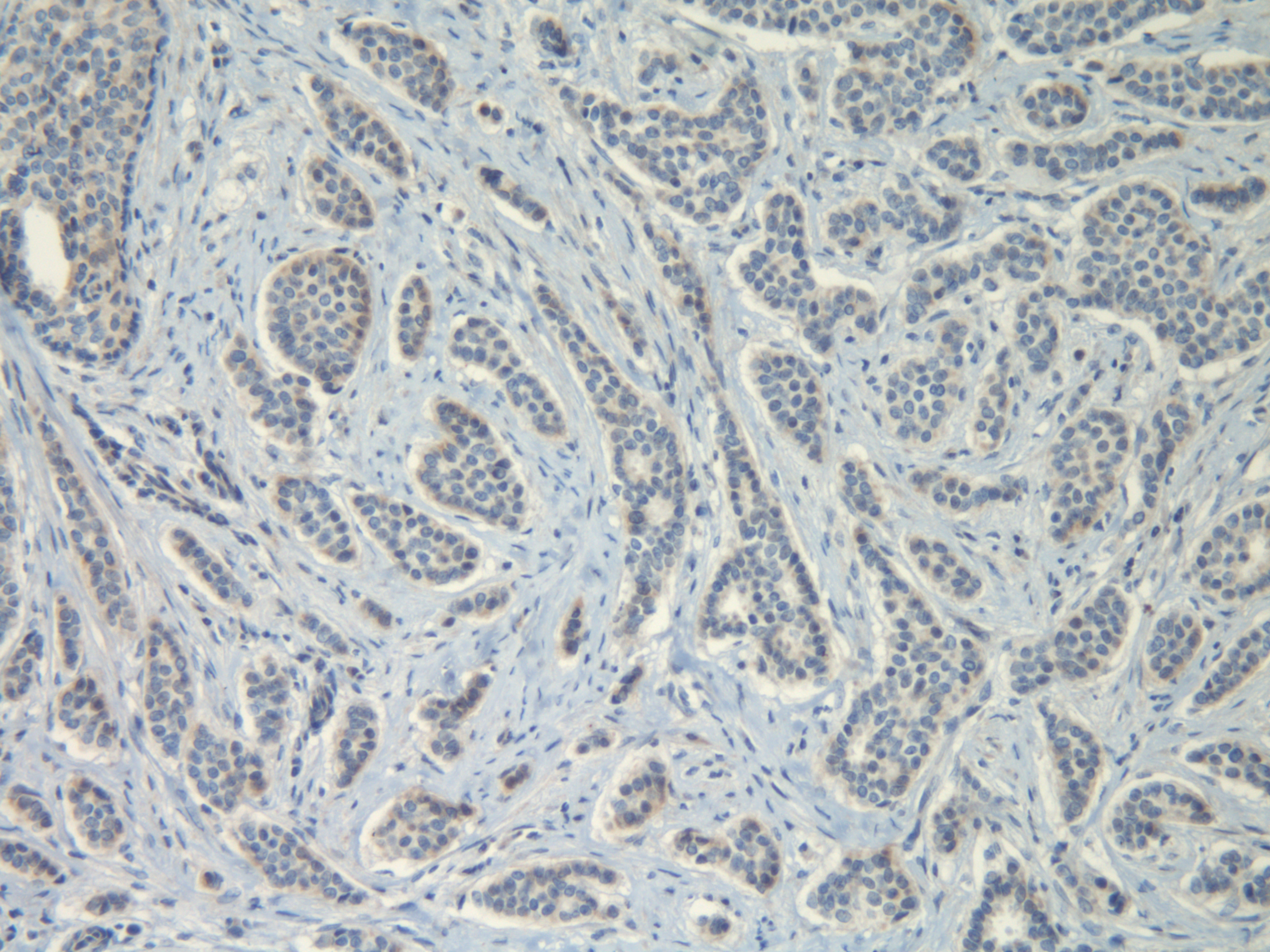

Supplement: Supplementary file 5 — Source Data for Expanded View [file EMMM-12-e10491-s012.zip › EV_source_data/Fig_EV5/Fig_EV5A_Grade_1.tif]

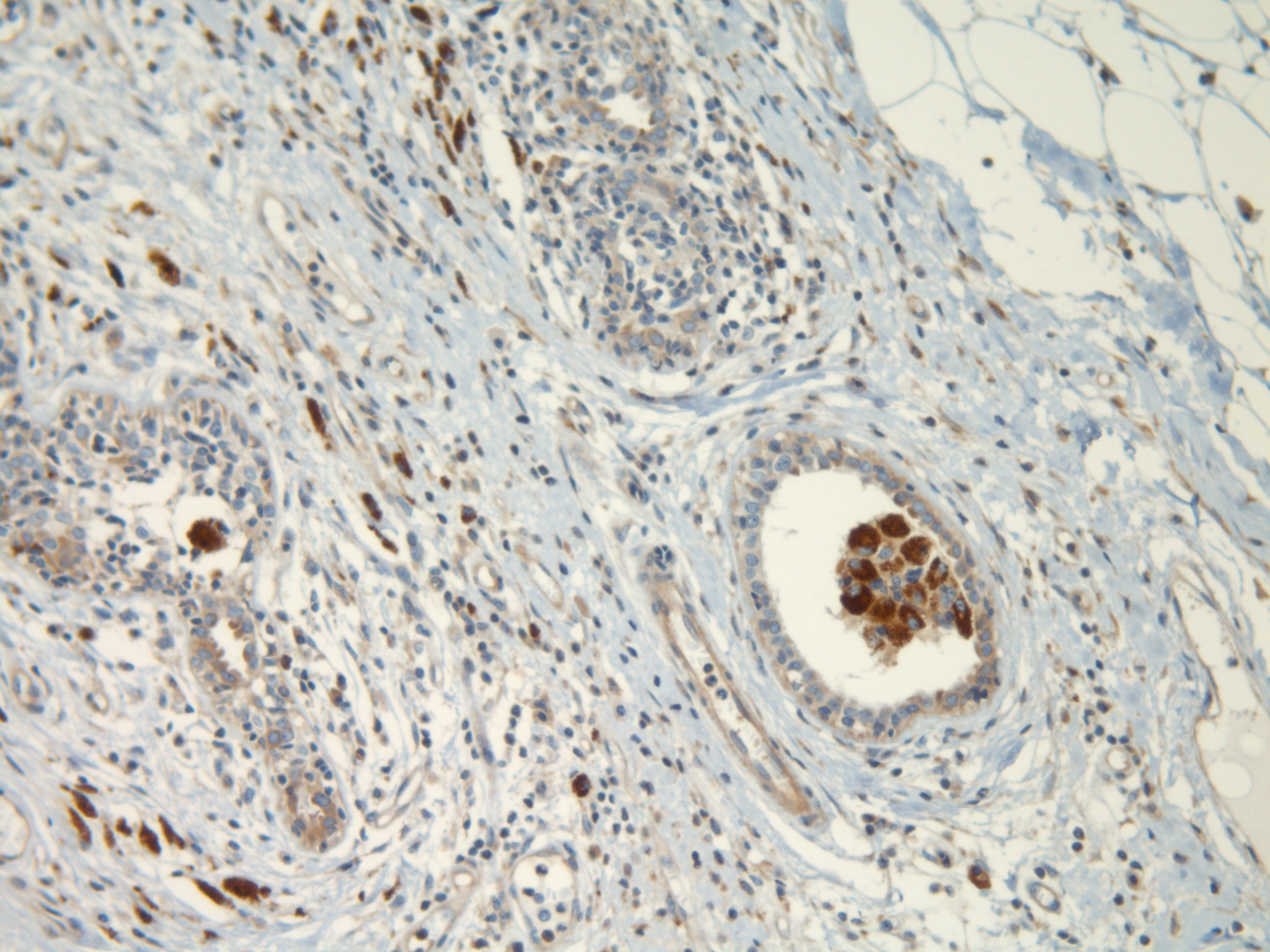

Supplement: Supplementary file 5 — Source Data for Expanded View [file EMMM-12-e10491-s012.zip › EV_source_data/Fig_EV5/Fig_EV5A_Grade_2.tif]

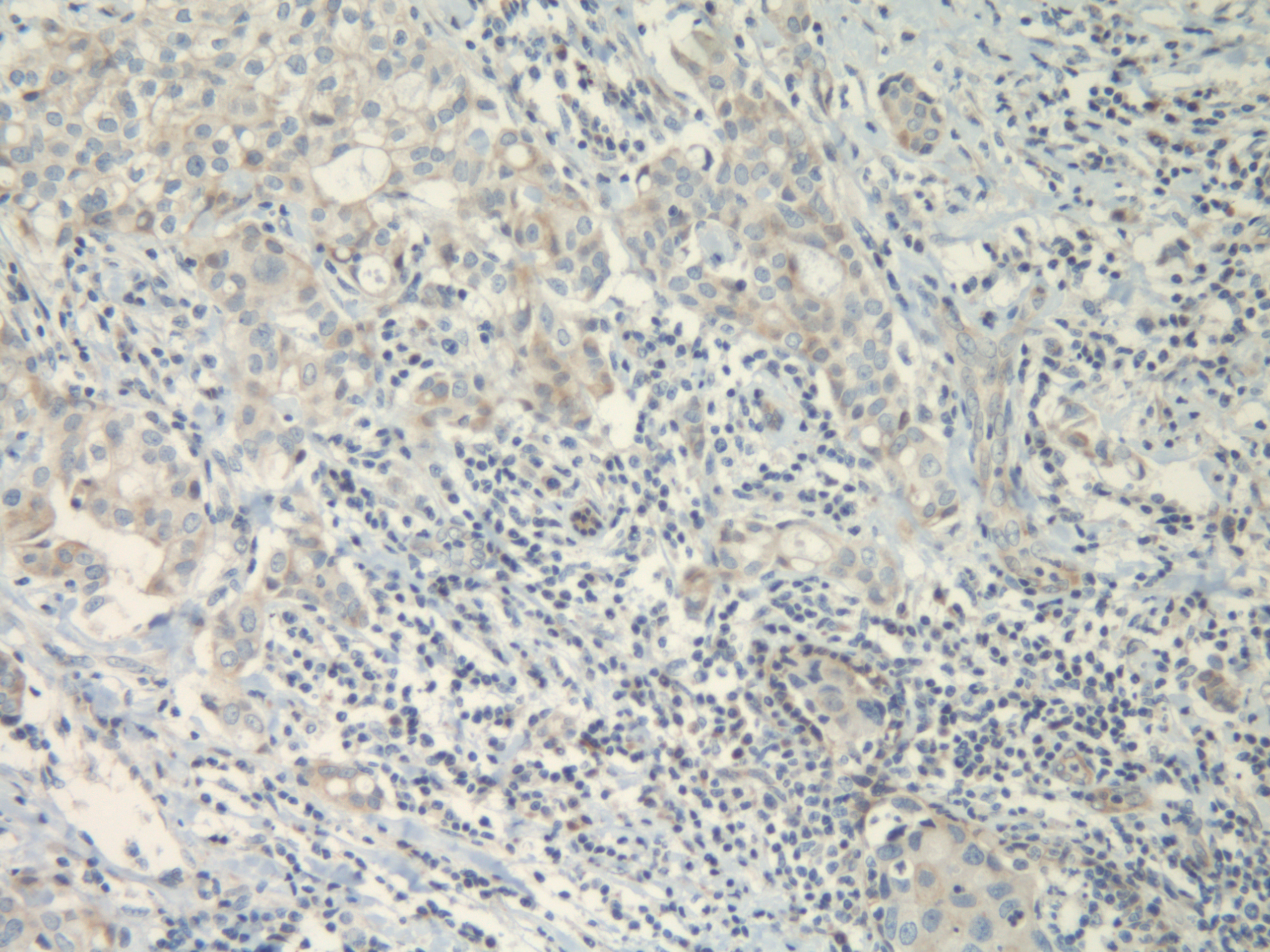

Supplement: Supplementary file 5 — Source Data for Expanded View [file EMMM-12-e10491-s012.zip › EV_source_data/Fig_EV5/Fig_EV5A_Grade_3.tif]

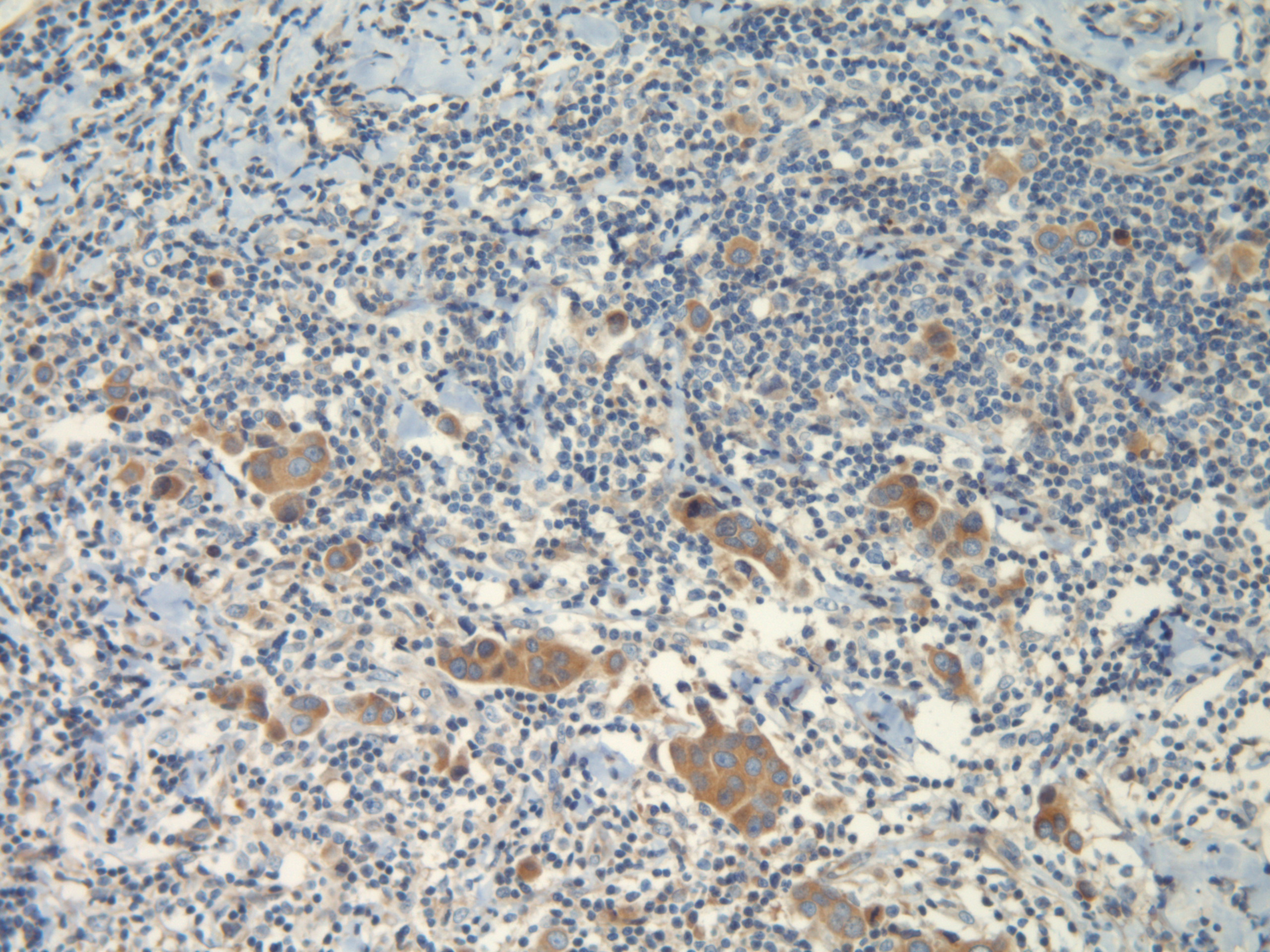

Supplement: Supplementary file 5 — Source Data for Expanded View [file EMMM-12-e10491-s012.zip › EV_source_data/Fig_EV5/Fig_EV5A_Grade_4.tif]

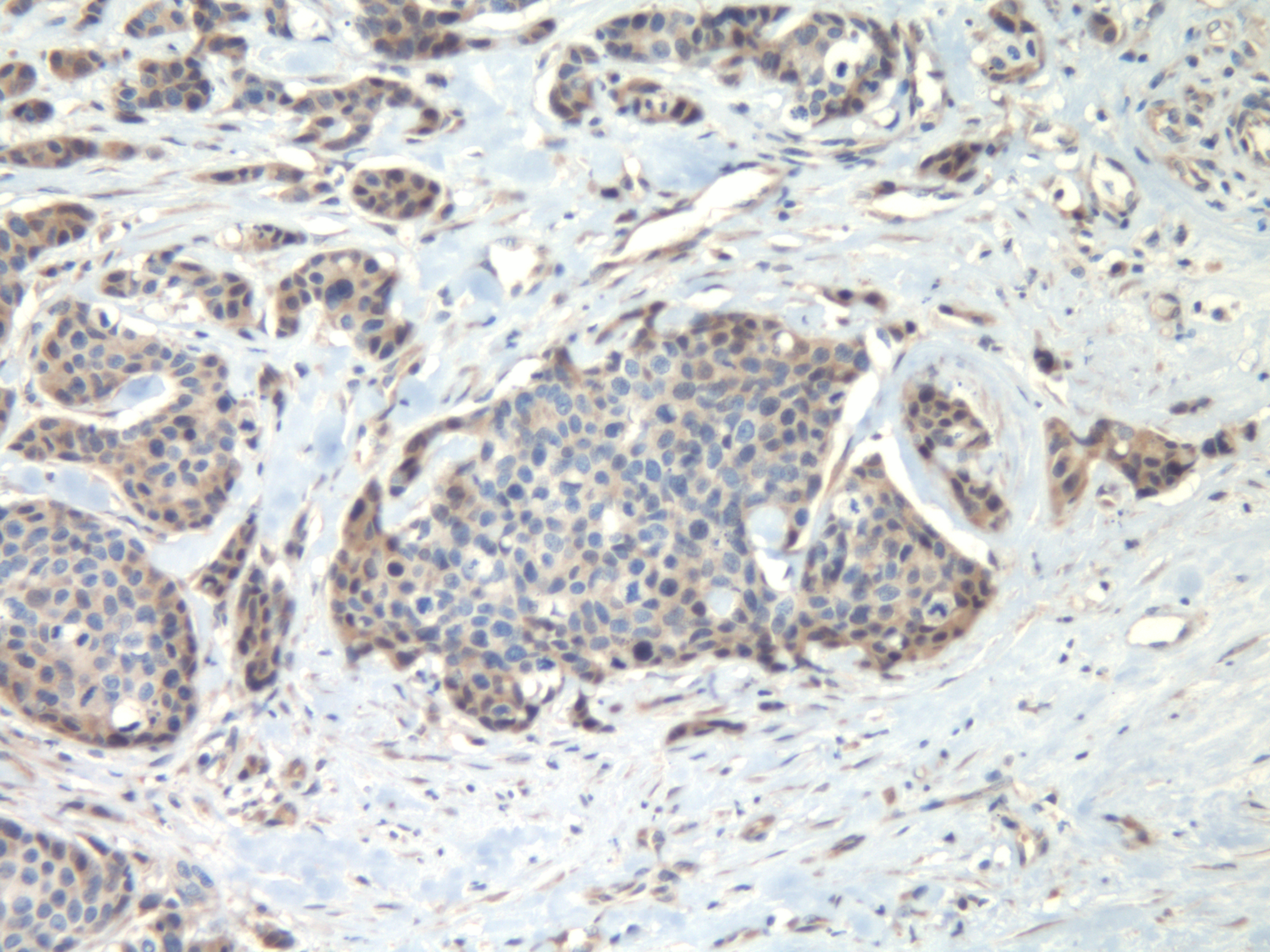

Supplement: Supplementary file 5 — Source Data for Expanded View [file EMMM-12-e10491-s012.zip › EV_source_data/Fig_EV5/Fig_EV5B.tif]

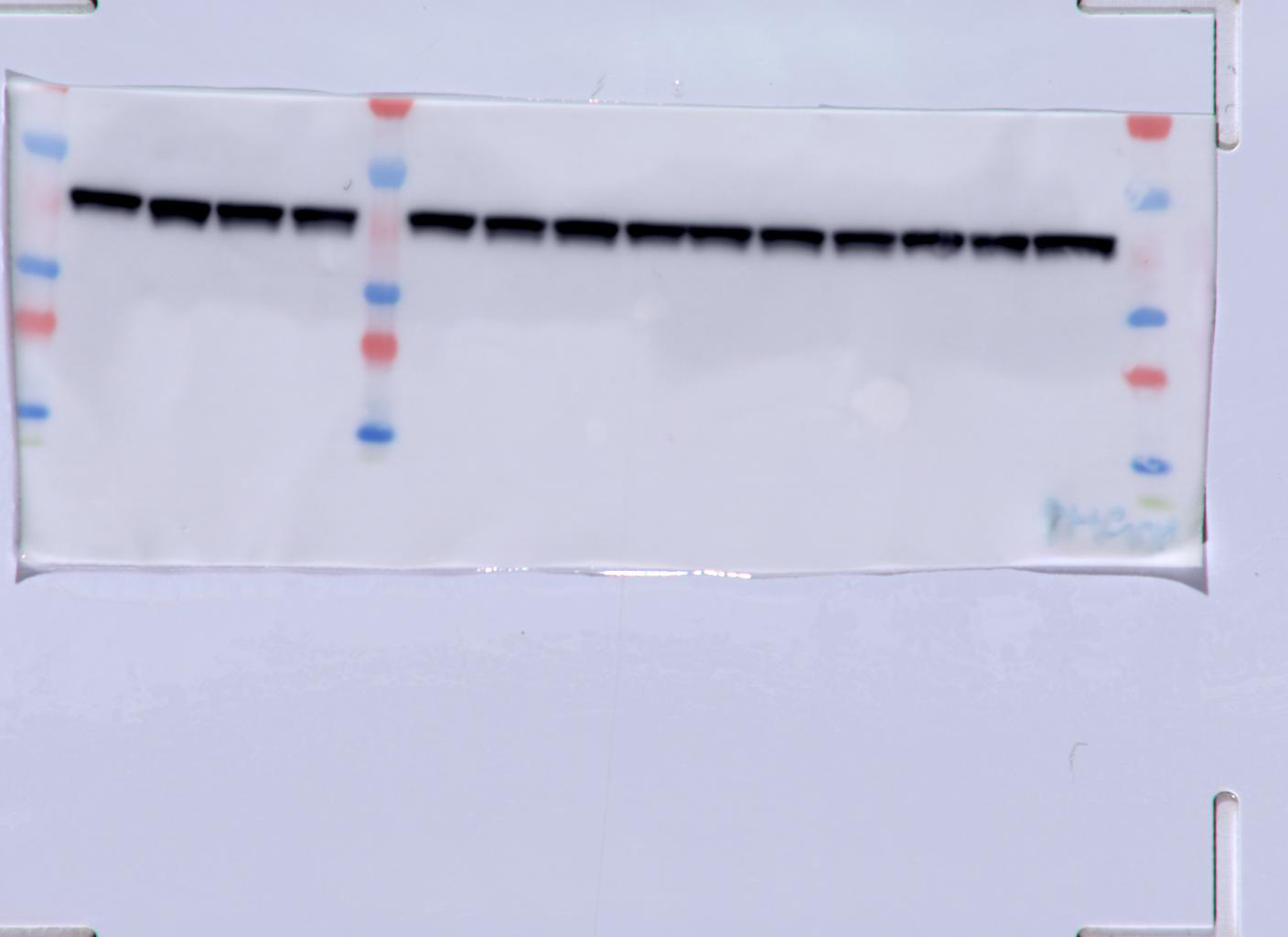

Supplement: Supplementary file 5 — Source Data for Expanded View [file EMMM-12-e10491-s012.zip › EV_source_data/Fig_EV6/Fig_EV6A,B_WB_for_Actin.jpg]

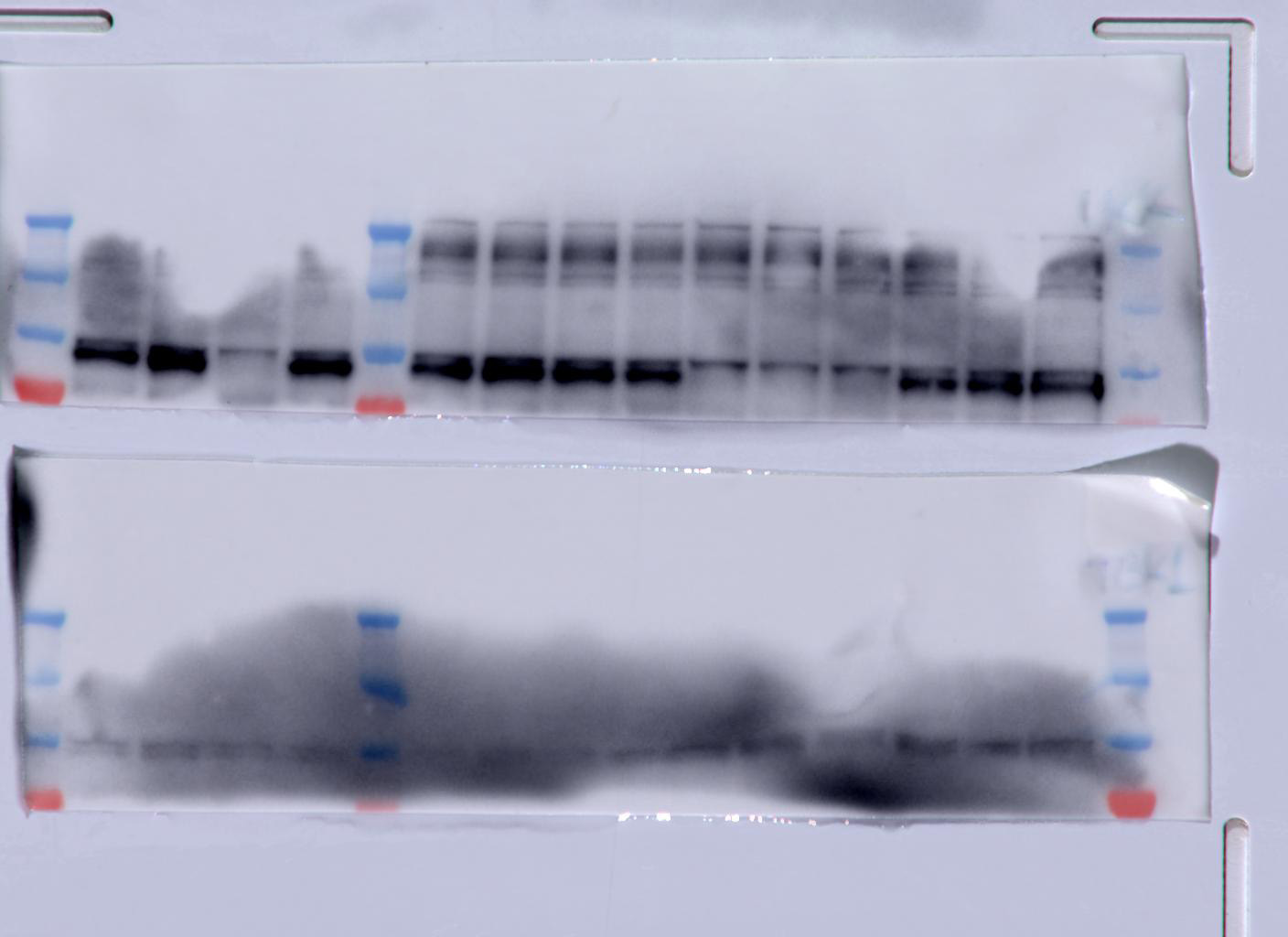

Supplement: Supplementary file 5 — Source Data for Expanded View [file EMMM-12-e10491-s012.zip › EV_source_data/Fig_EV6/Fig_EV6A,B_WB_for_IKKe.jpg]

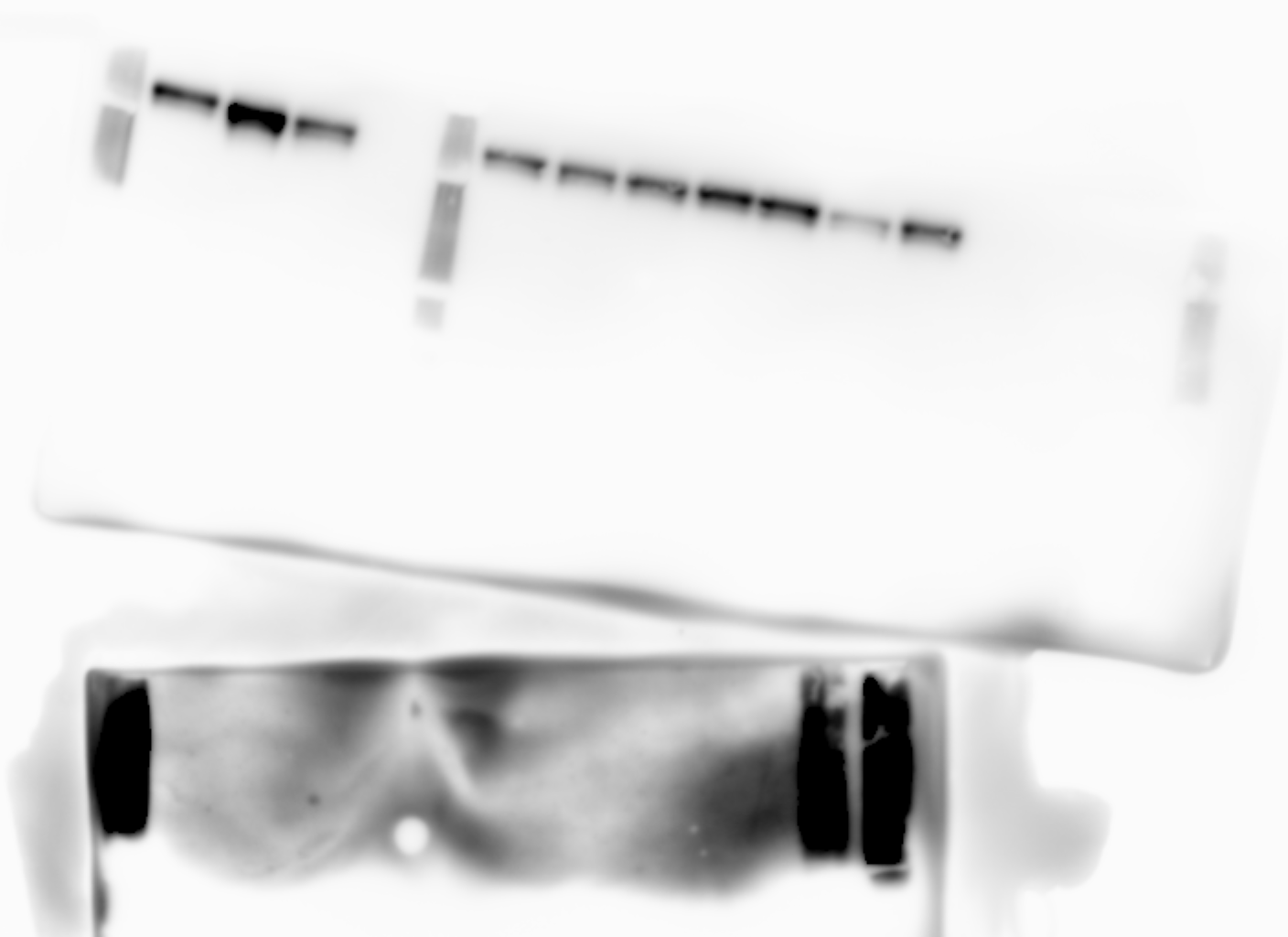

Supplement: Supplementary file 5 — Source Data for Expanded View [file EMMM-12-e10491-s012.zip › EV_source_data/Fig_EV6/Fig_EV6A,B_WB_for_PHGDH.tif]

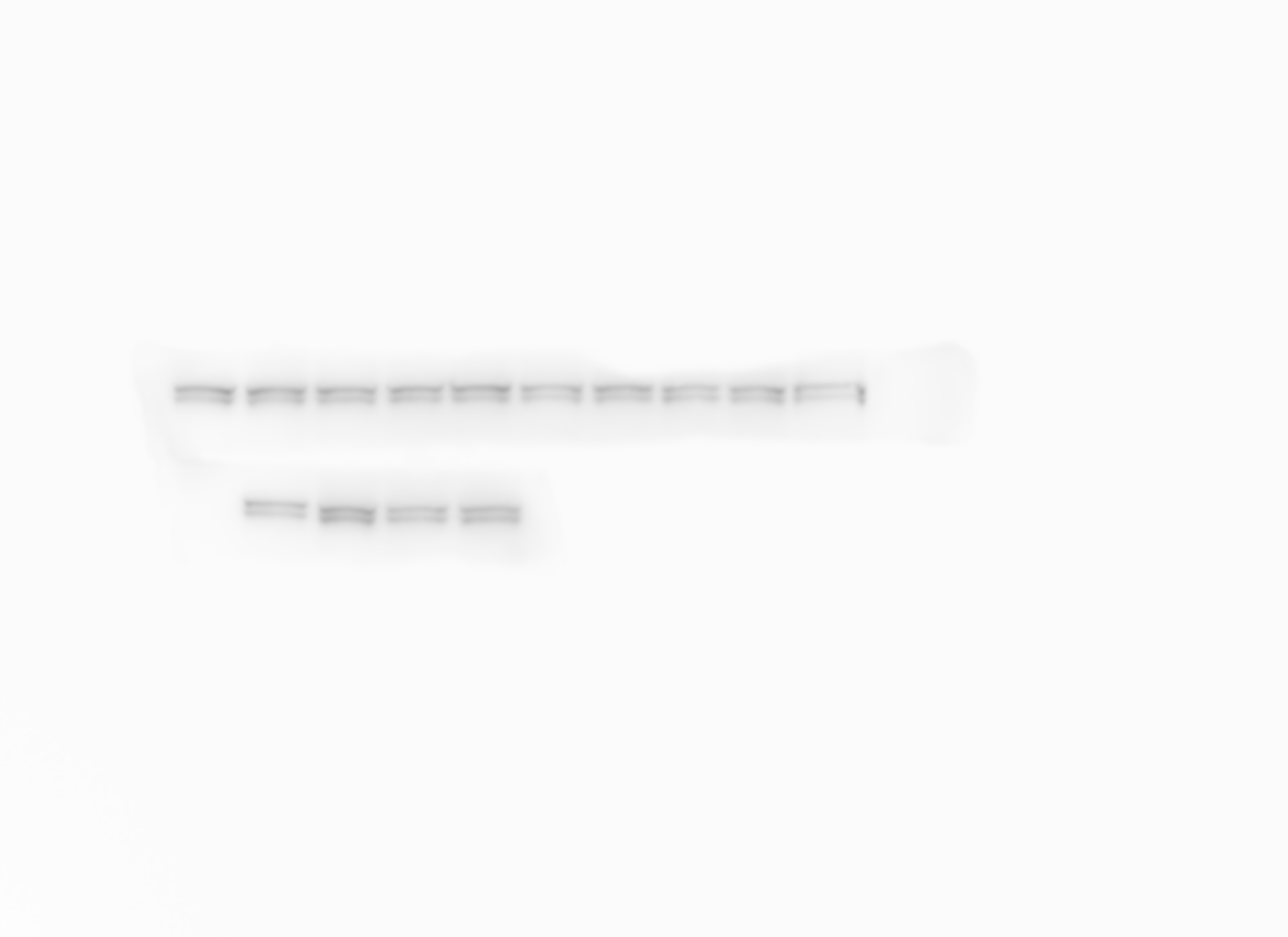

Supplement: Supplementary file 5 — Source Data for Expanded View [file EMMM-12-e10491-s012.zip › EV_source_data/Fig_EV6/Fig_EV6A,B_WB_for_TBK1.tif]

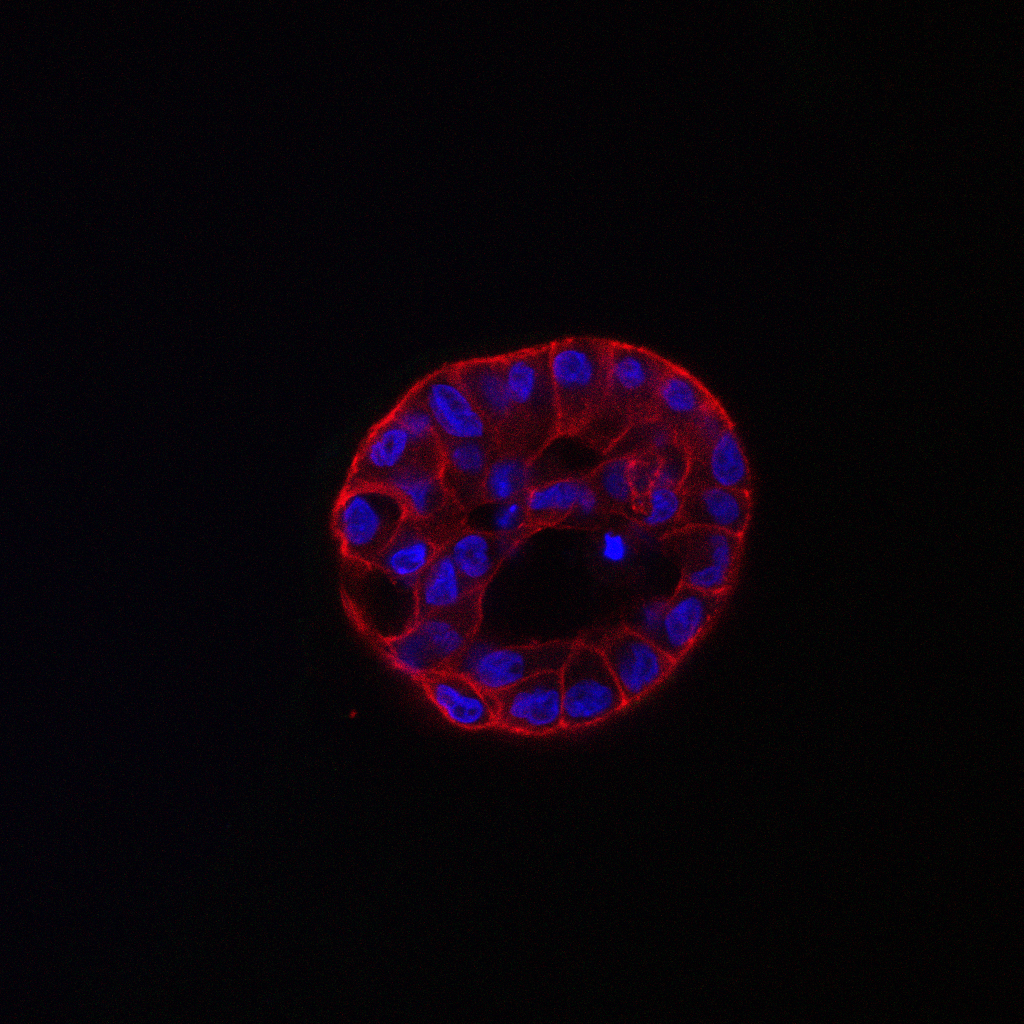

Supplement: Supplementary file 7 — Source Data for Figure 1 [file EMMM-12-e10491-s005.zip › Fig1/Fig_1B_(almost_filled).tif]

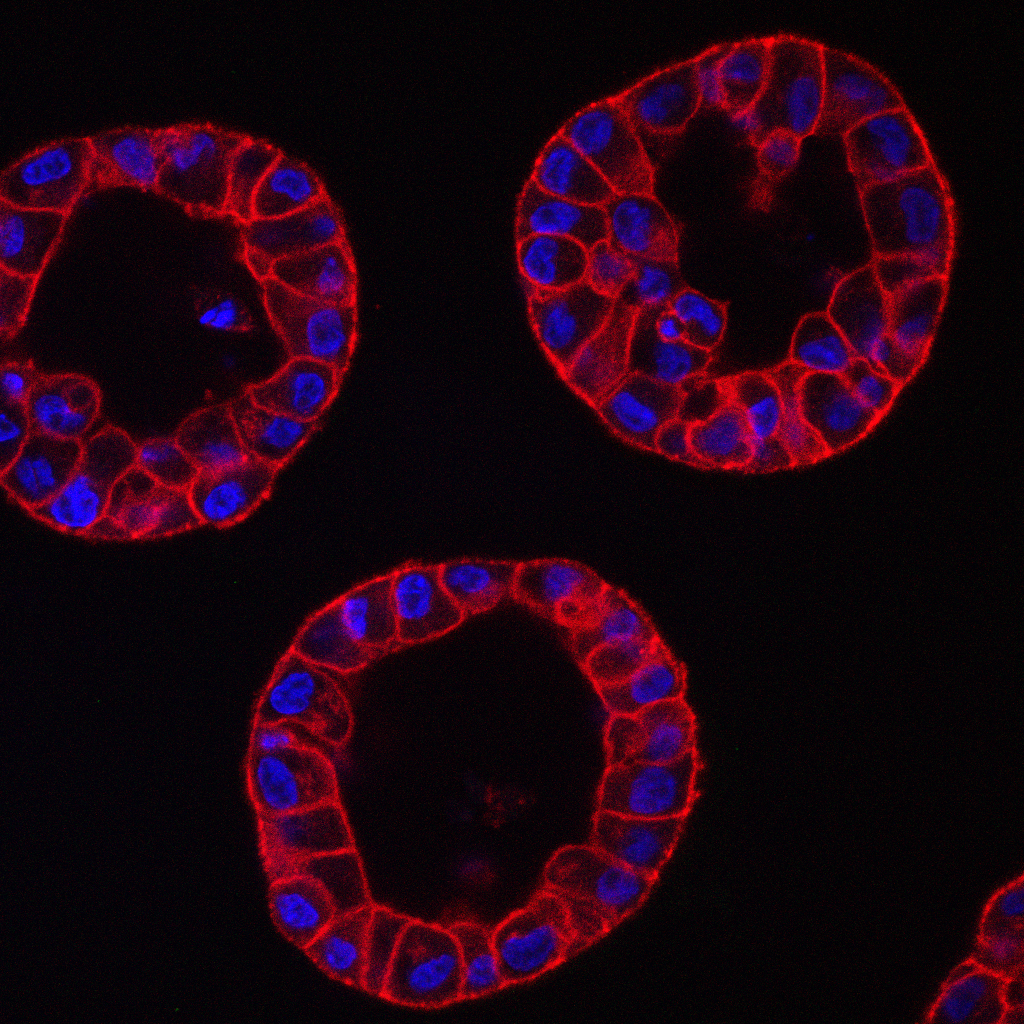

Supplement: Supplementary file 7 — Source Data for Figure 1 [file EMMM-12-e10491-s005.zip › Fig1/Fig_1B_(clear).tif]

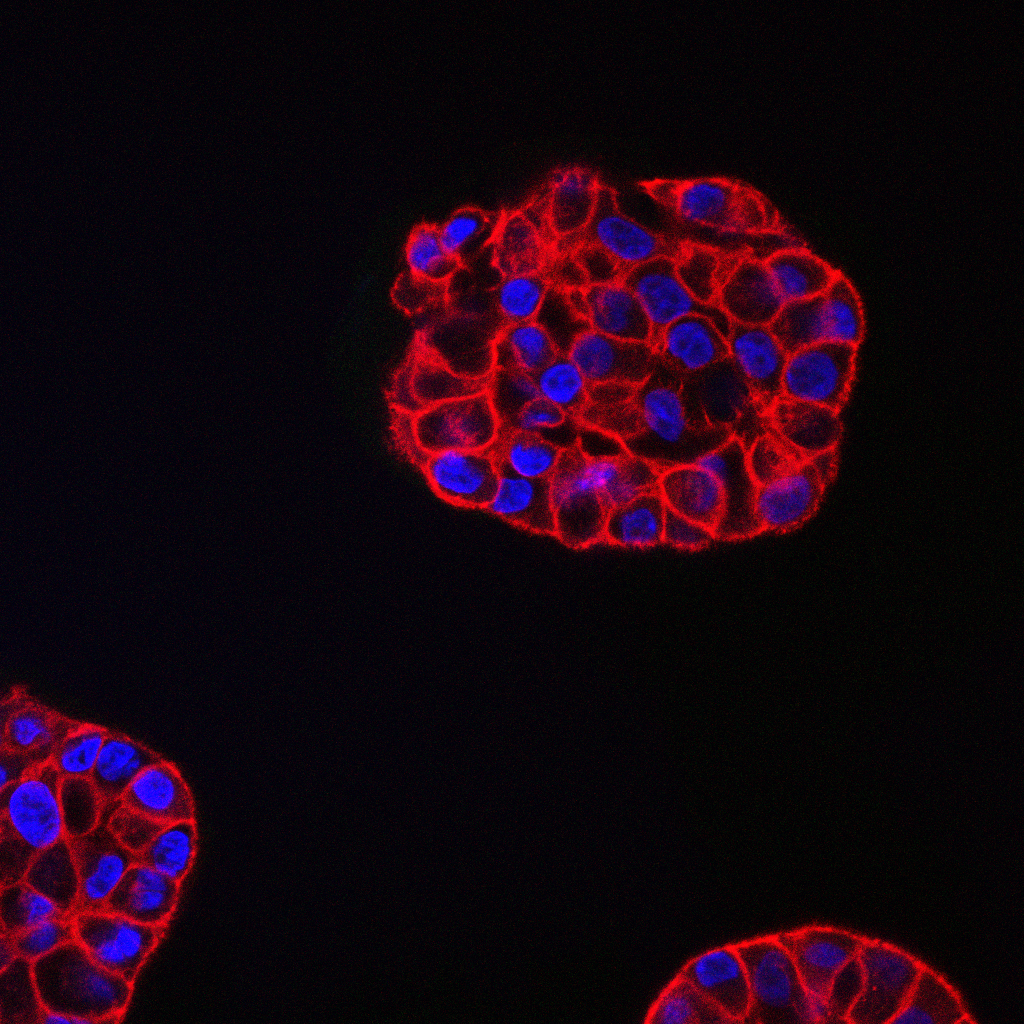

Supplement: Supplementary file 7 — Source Data for Figure 1 [file EMMM-12-e10491-s005.zip › Fig1/Fig_1B_(filled).tif]

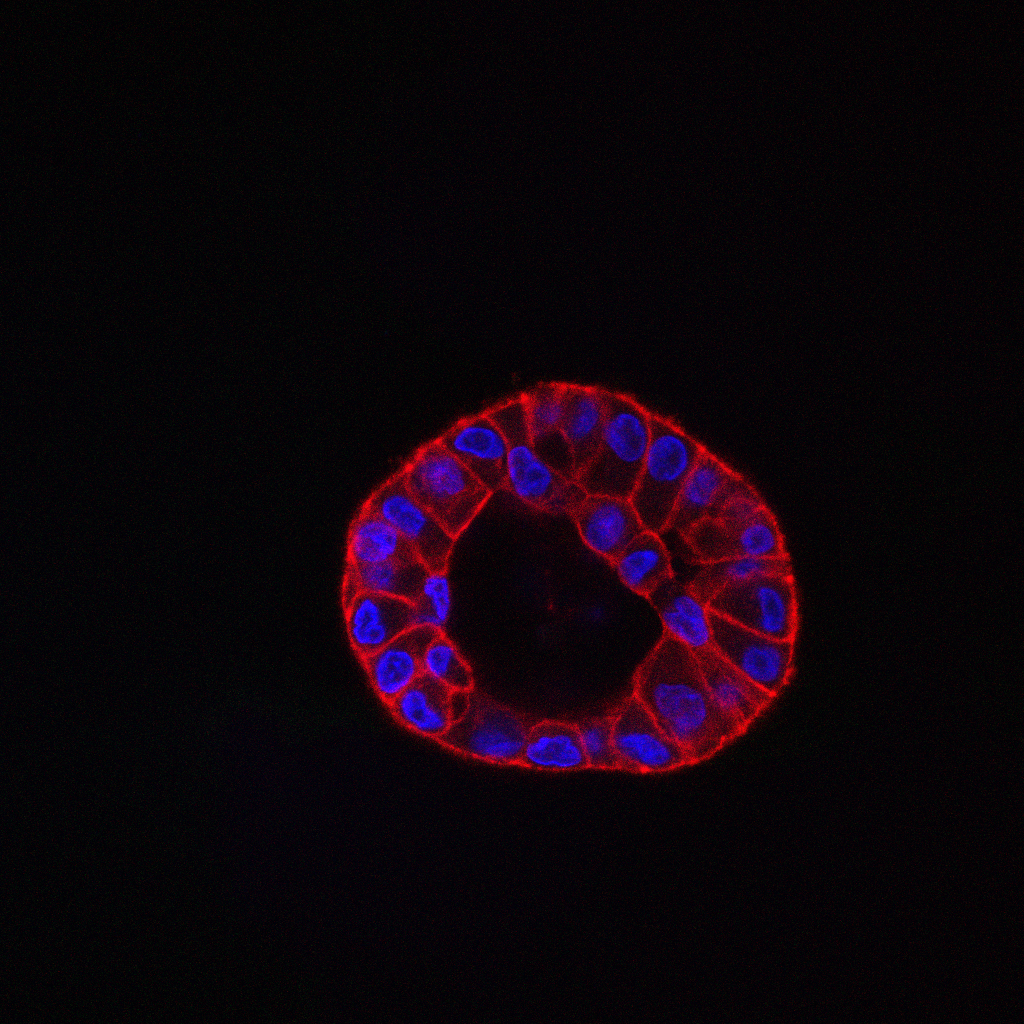

Supplement: Supplementary file 7 — Source Data for Figure 1 [file EMMM-12-e10491-s005.zip › Fig1/Fig_1B_(partially_clear).tif]

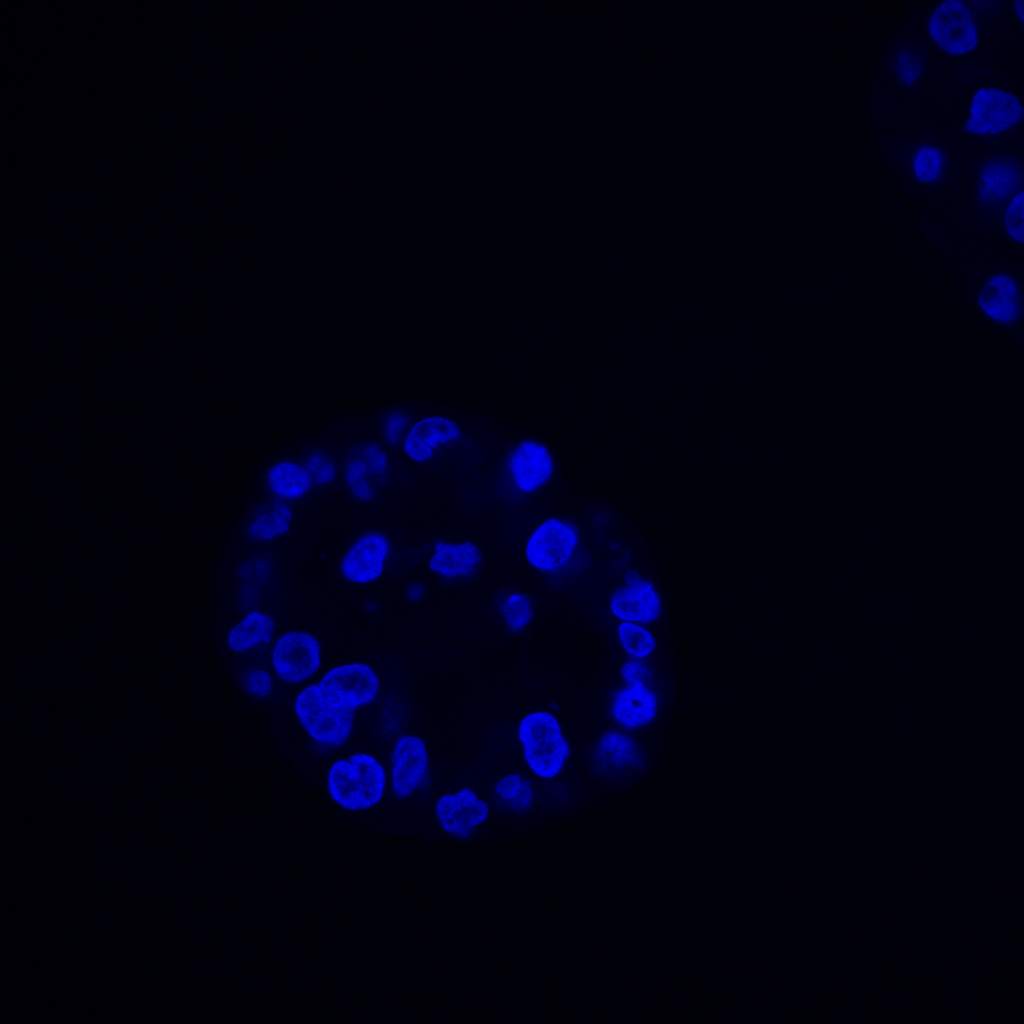

Supplement: Supplementary file 7 — Source Data for Figure 1 [file EMMM-12-e10491-s005.zip › Fig1/Fig_1D_DAPI.tif]

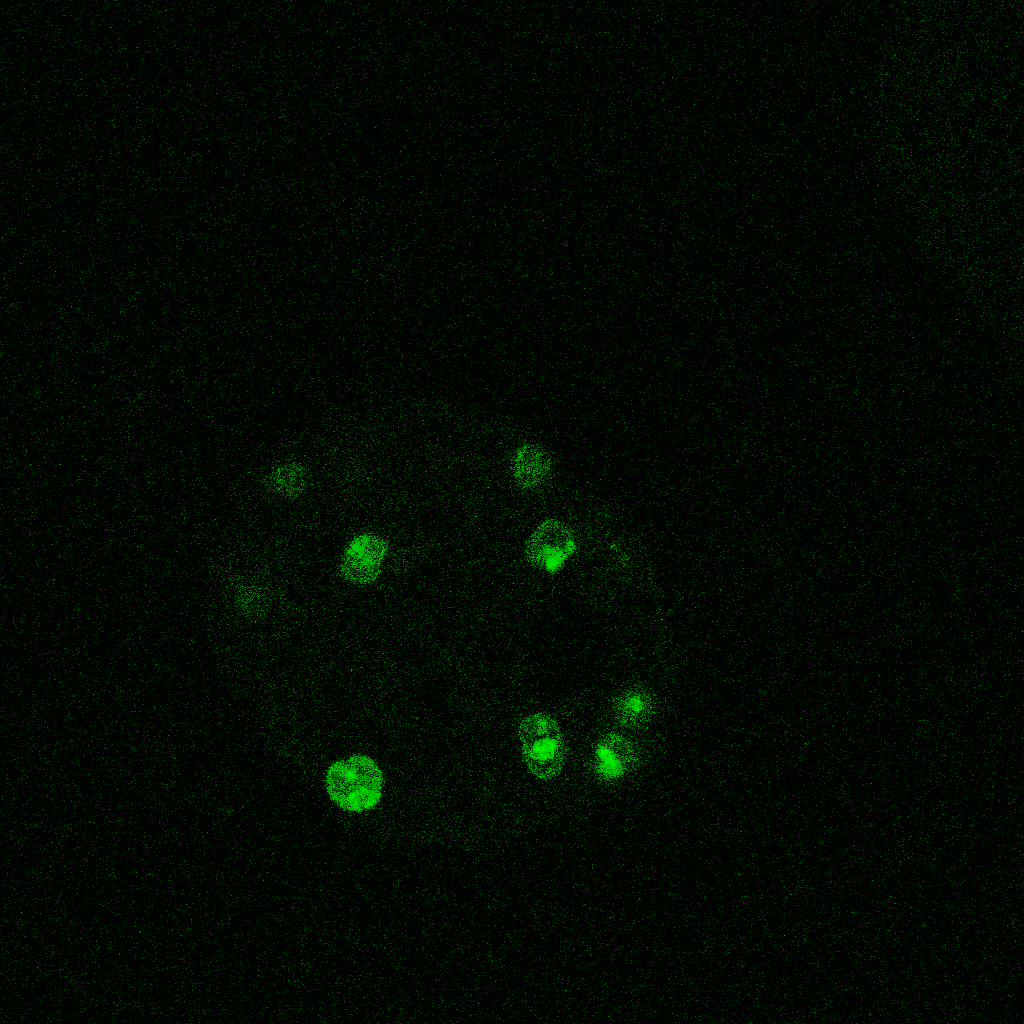

Supplement: Supplementary file 7 — Source Data for Figure 1 [file EMMM-12-e10491-s005.zip › Fig1/Fig_1D_ki67.tif]

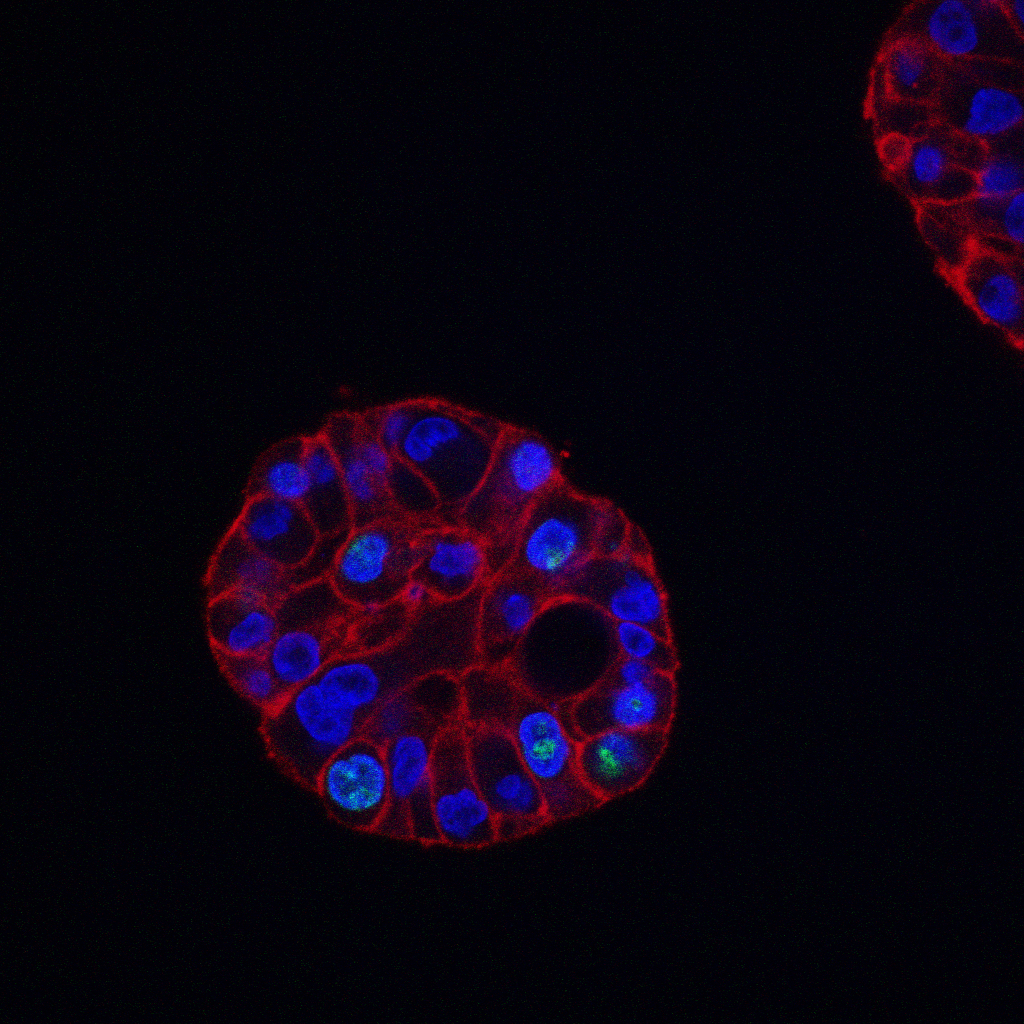

Supplement: Supplementary file 7 — Source Data for Figure 1 [file EMMM-12-e10491-s005.zip › Fig1/Fig_1D_merge.tif]

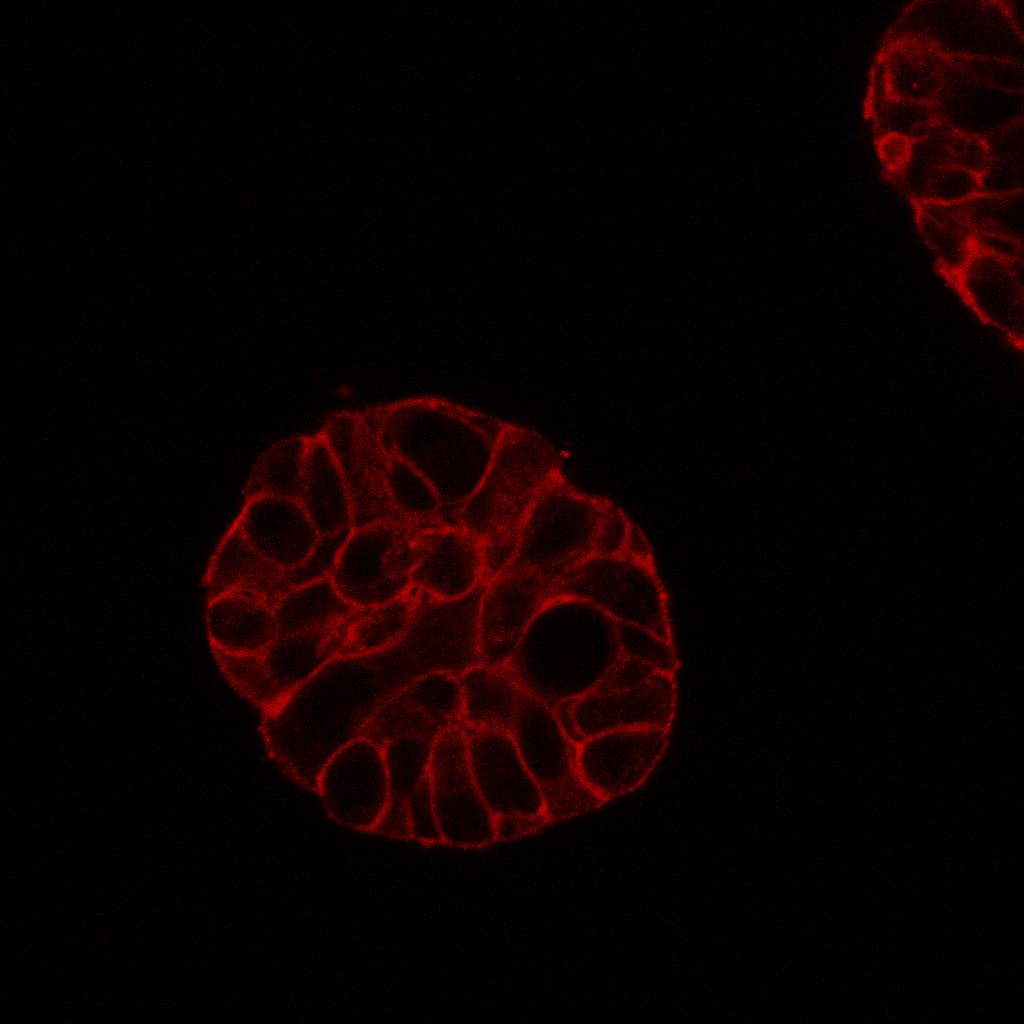

Supplement: Supplementary file 7 — Source Data for Figure 1 [file EMMM-12-e10491-s005.zip › Fig1/Fig_1D_phalloidin.tif]

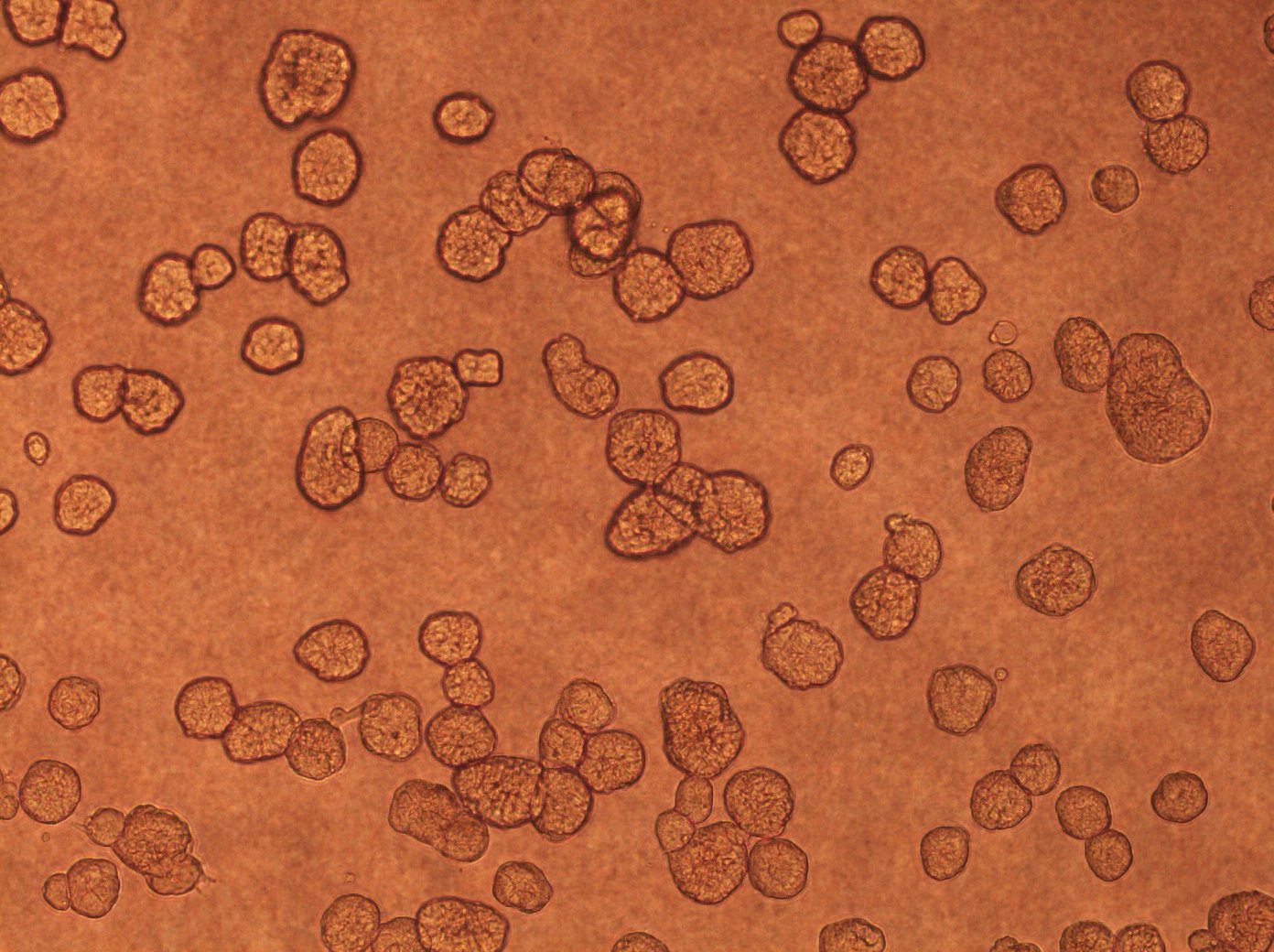

Supplement: Supplementary file 7 — Source Data for Figure 1 [file EMMM-12-e10491-s005.zip › Fig1/Fig_1G_invasive_protrusion_1.TIF]

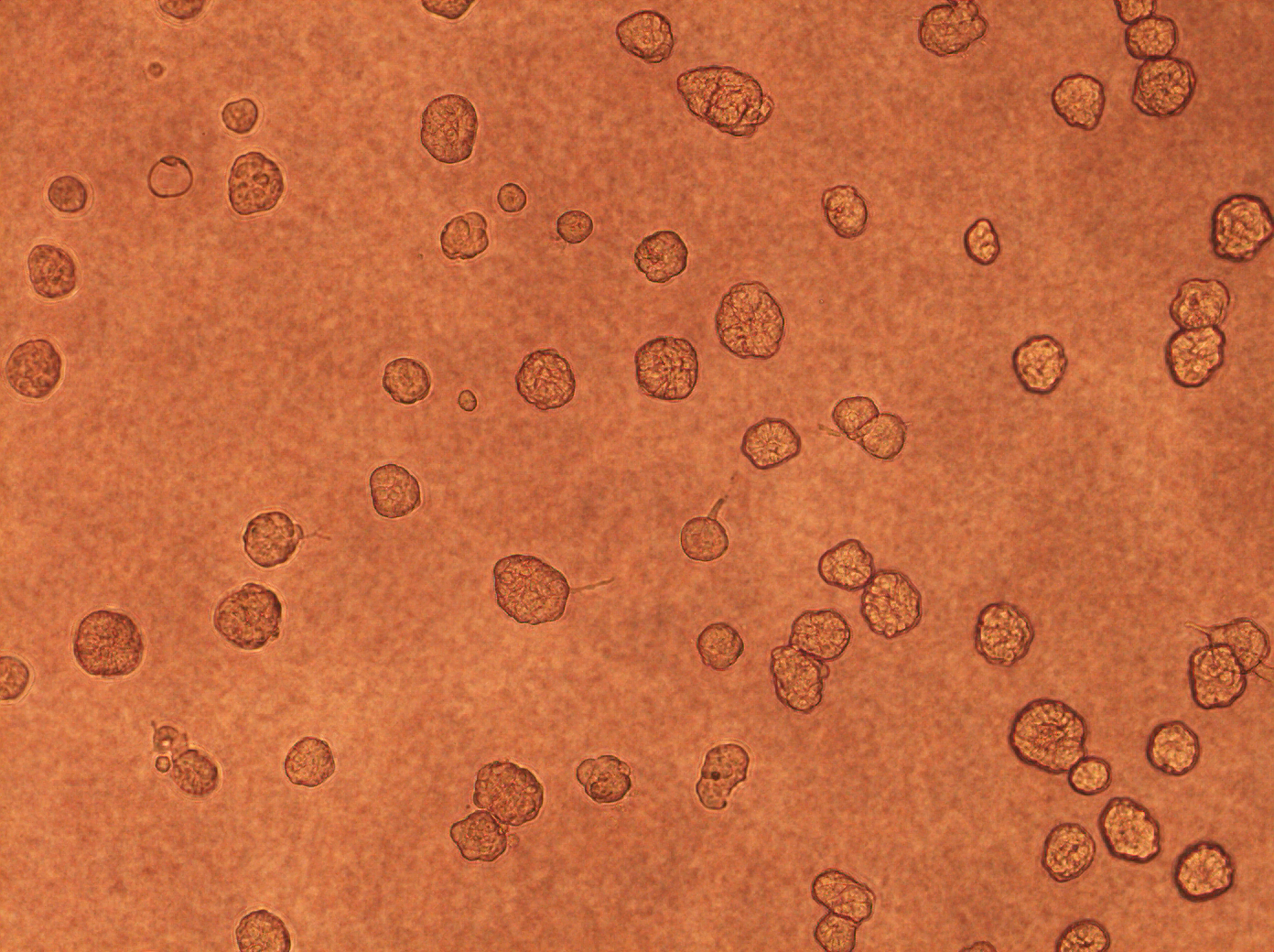

Supplement: Supplementary file 7 — Source Data for Figure 1 [file EMMM-12-e10491-s005.zip › Fig1/Fig_1G_invasive_protrusion_2.TIF]

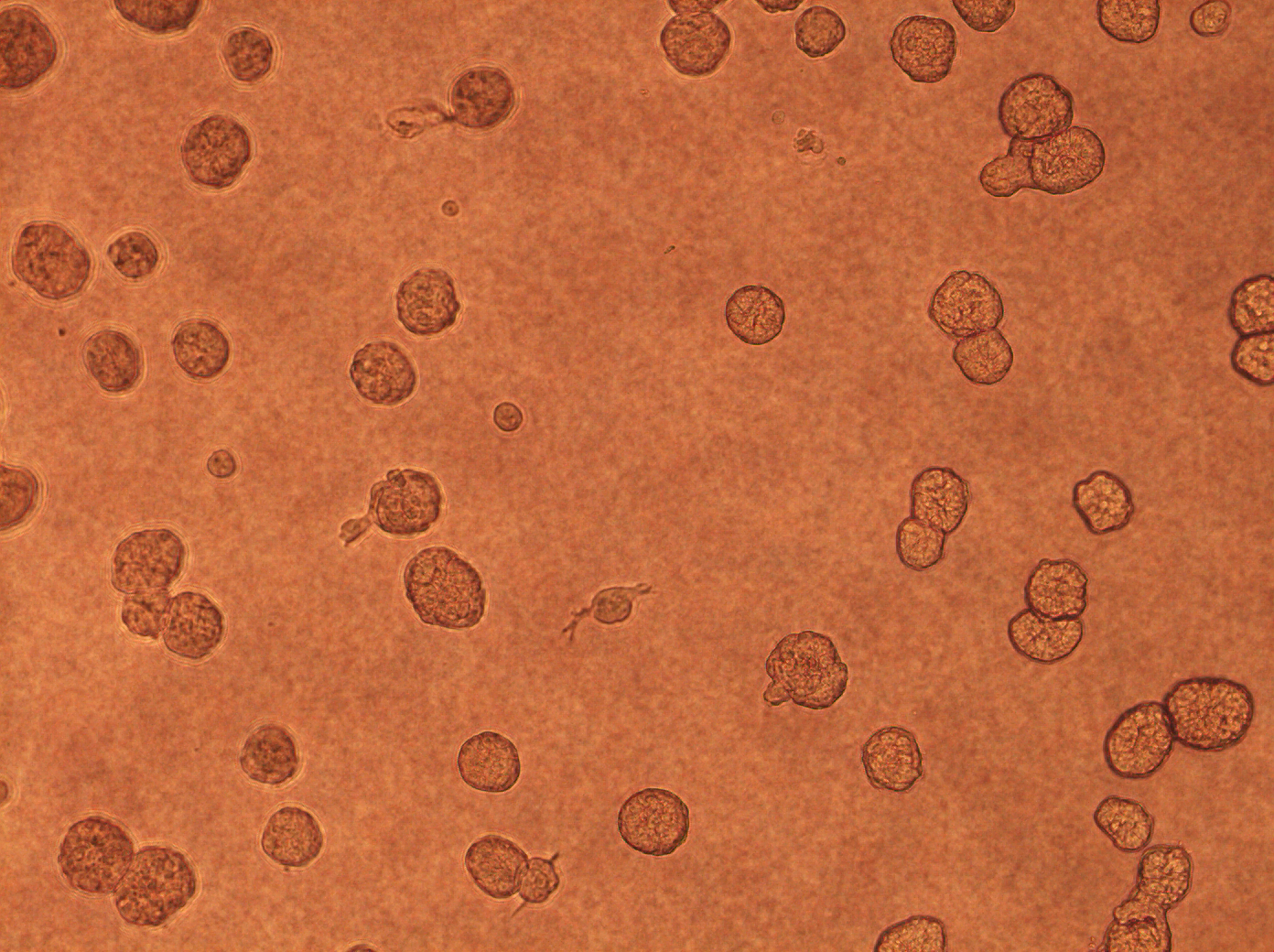

Supplement: Supplementary file 7 — Source Data for Figure 1 [file EMMM-12-e10491-s005.zip › Fig1/Fig_1G_invasive_protrusion_3.TIF]

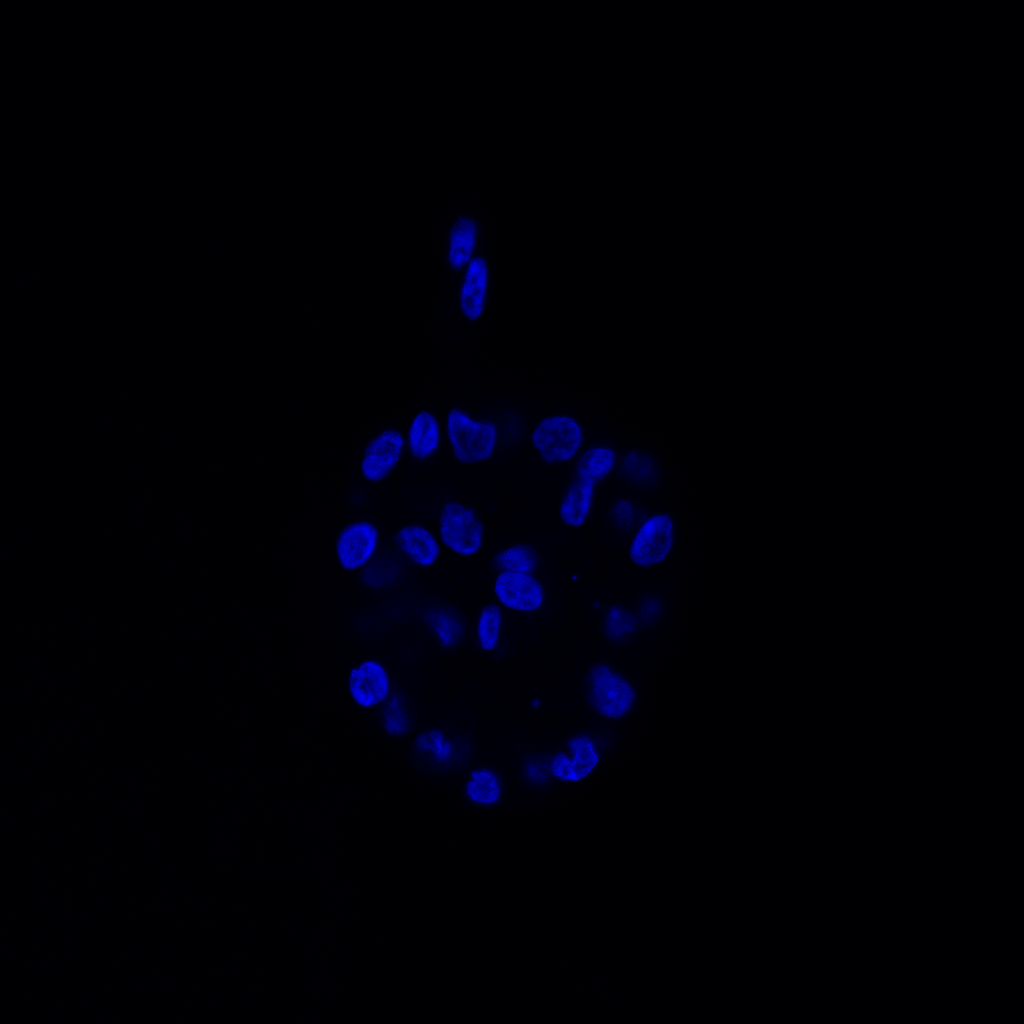

Supplement: Supplementary file 7 — Source Data for Figure 1 [file EMMM-12-e10491-s005.zip › Fig1/Fig_1H_invasive_spheroid_1_(Hoechst_33342).TIF]

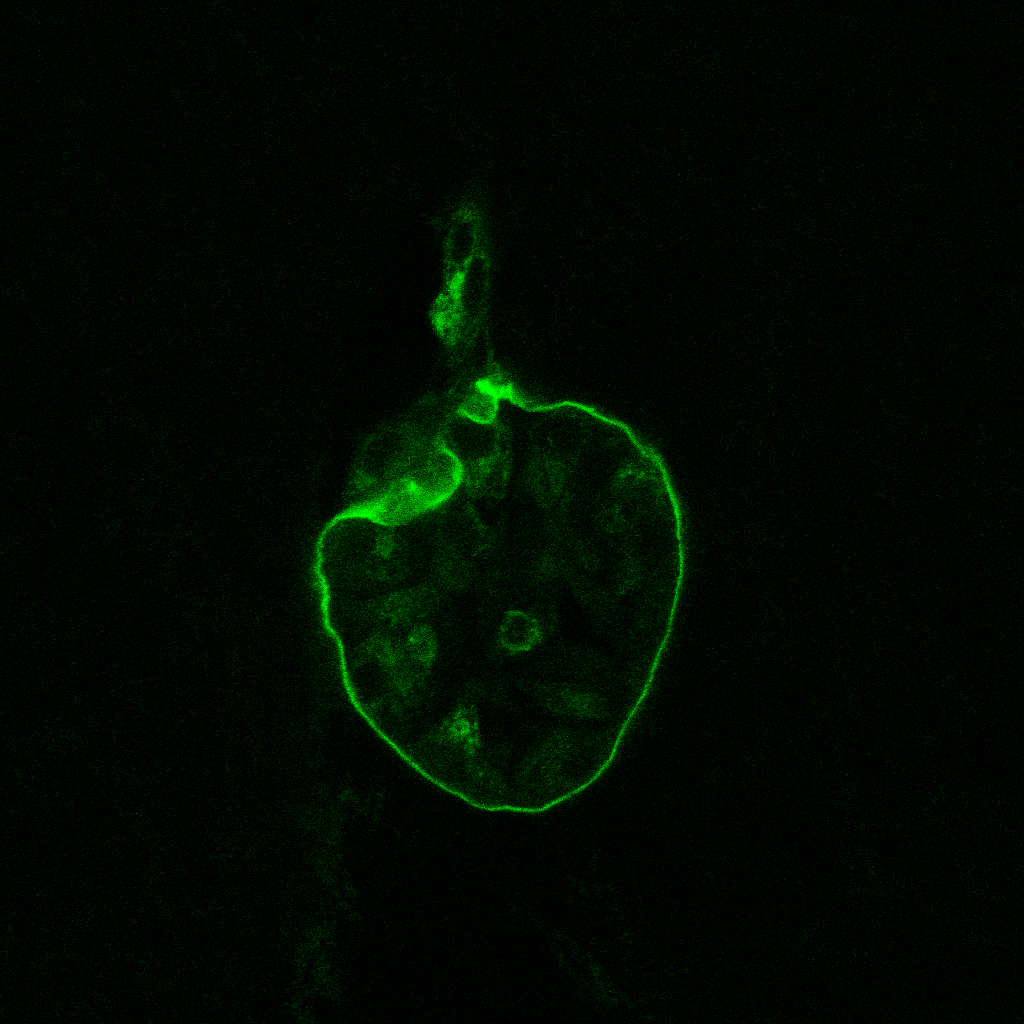

Supplement: Supplementary file 7 — Source Data for Figure 1 [file EMMM-12-e10491-s005.zip › Fig1/Fig_1H_invasive_spheroid_1_(laminin_V).tif]

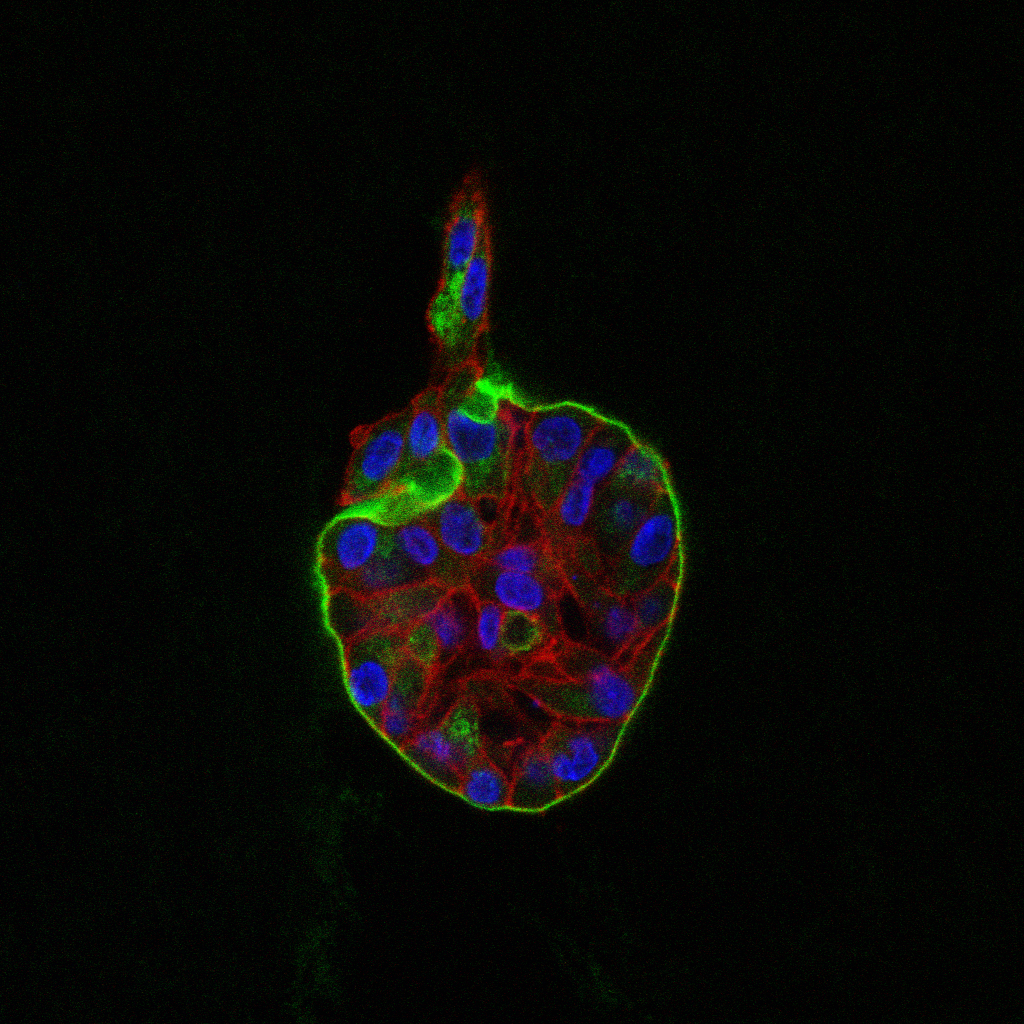

Supplement: Supplementary file 7 — Source Data for Figure 1 [file EMMM-12-e10491-s005.zip › Fig1/Fig_1H_invasive_spheroid_1_(merge).tif]

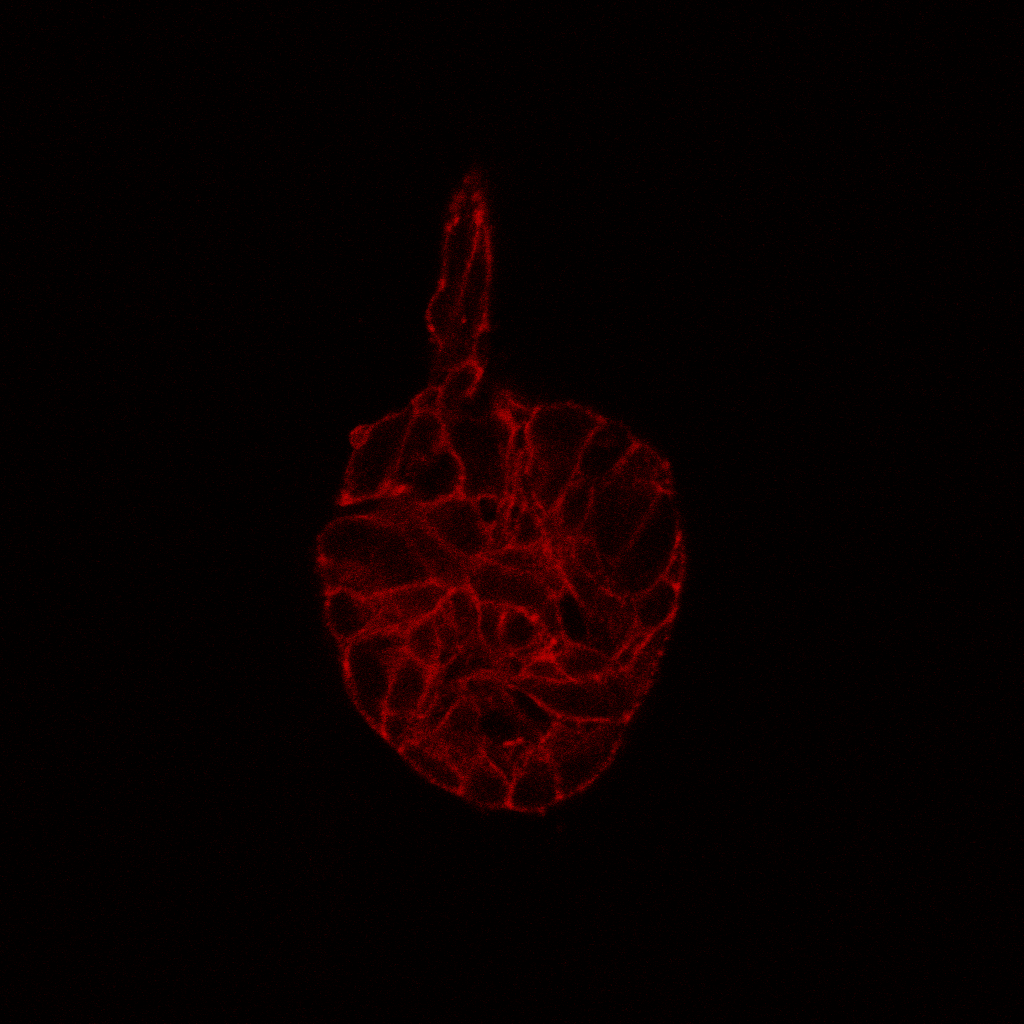

Supplement: Supplementary file 7 — Source Data for Figure 1 [file EMMM-12-e10491-s005.zip › Fig1/Fig_1H_invasive_spheroid_1_(phalloidin).tif]

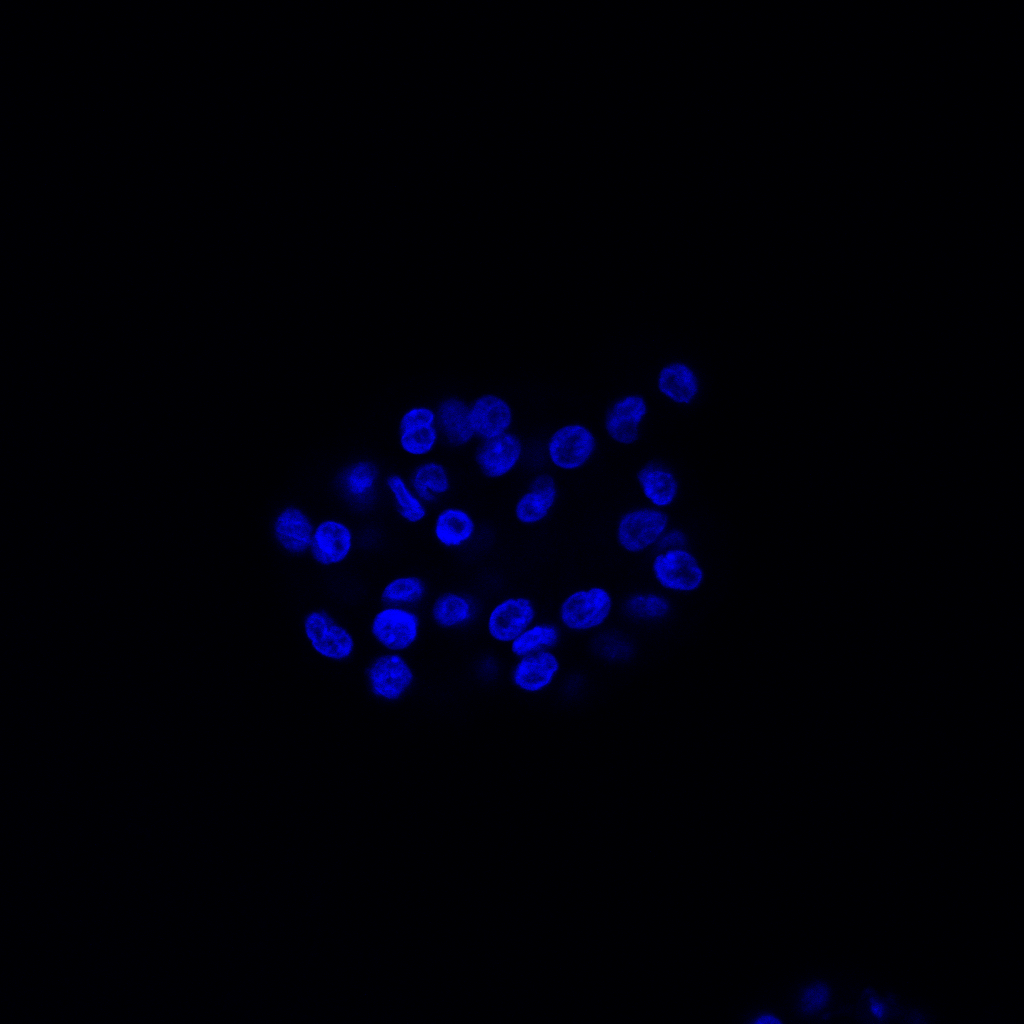

Supplement: Supplementary file 7 — Source Data for Figure 1 [file EMMM-12-e10491-s005.zip › Fig1/Fig_1H_invasive_spheroid_2_(Hoechst_33342).TIF]

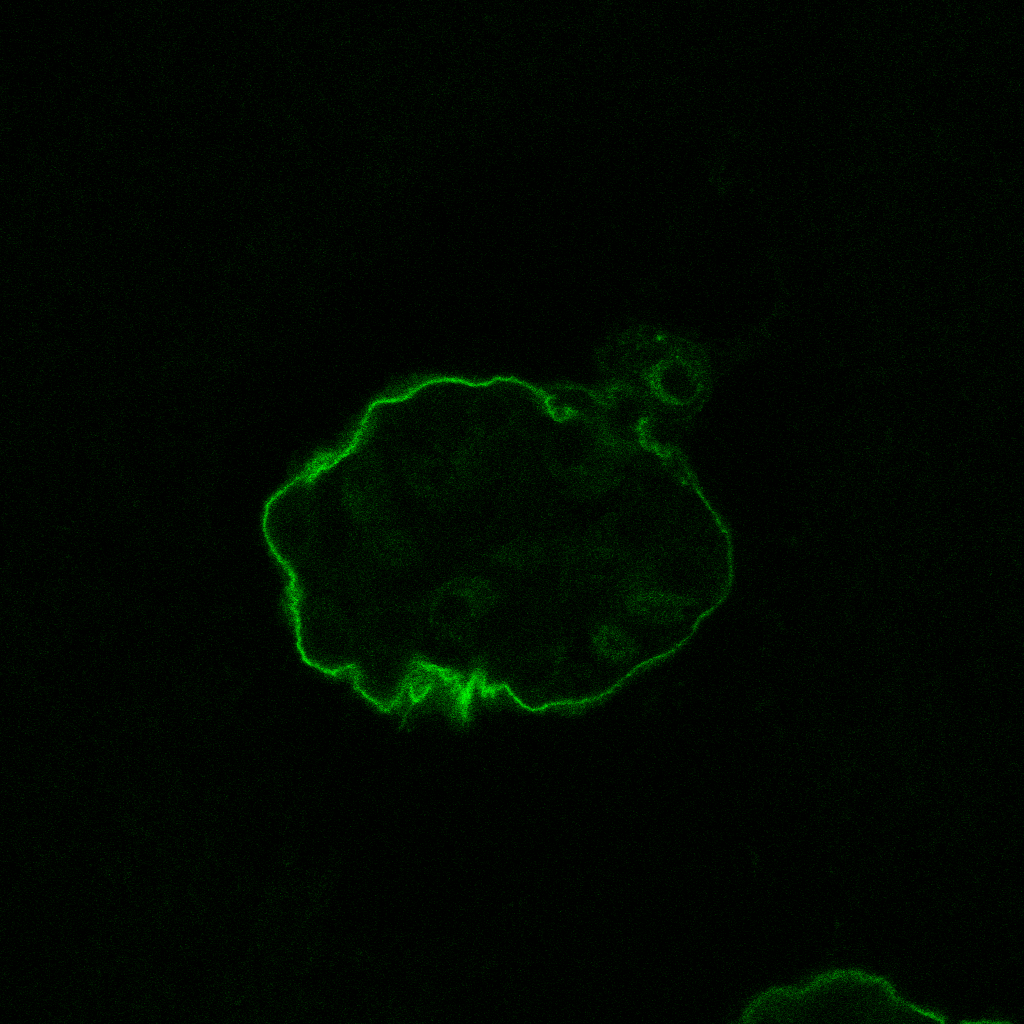

Supplement: Supplementary file 7 — Source Data for Figure 1 [file EMMM-12-e10491-s005.zip › Fig1/Fig_1H_invasive_spheroid_2_(laminin_V).tif]

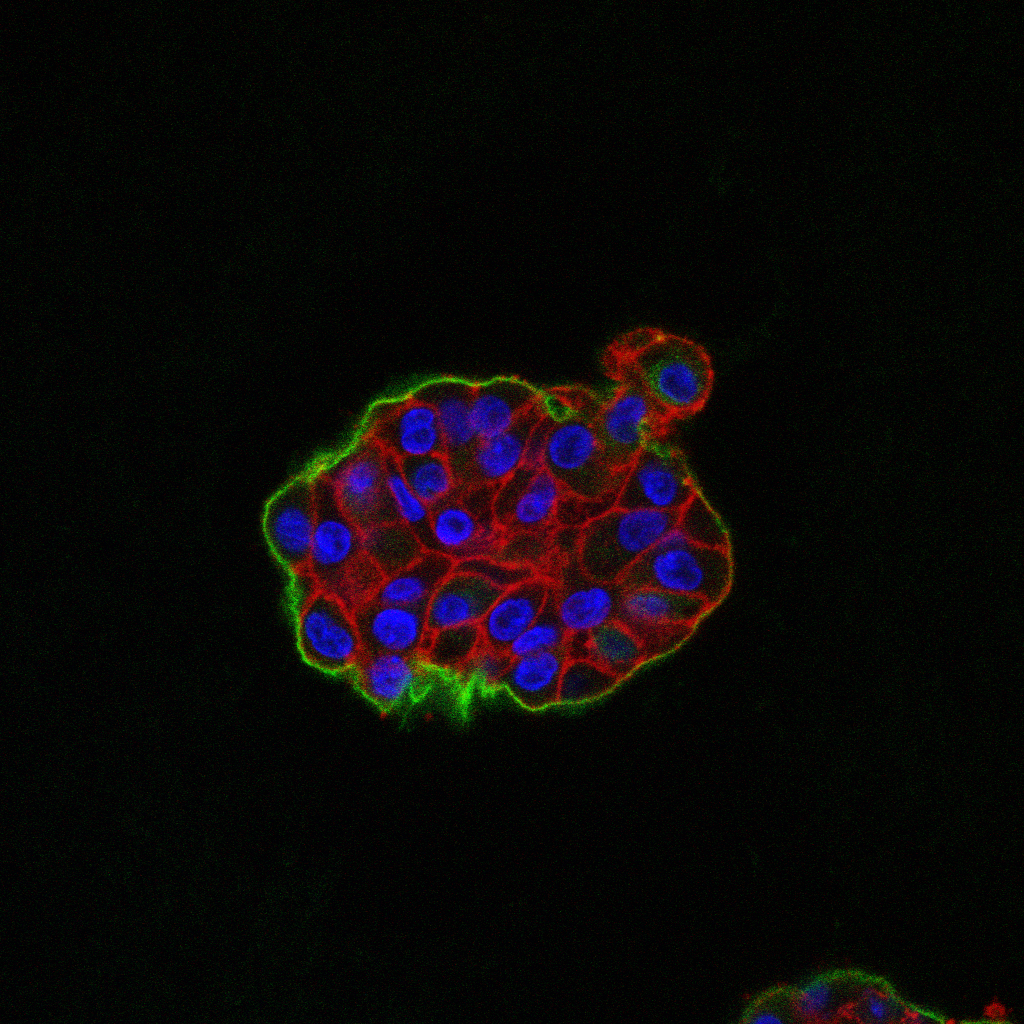

Supplement: Supplementary file 7 — Source Data for Figure 1 [file EMMM-12-e10491-s005.zip › Fig1/Fig_1H_invasive_spheroid_2_(merge).tif]

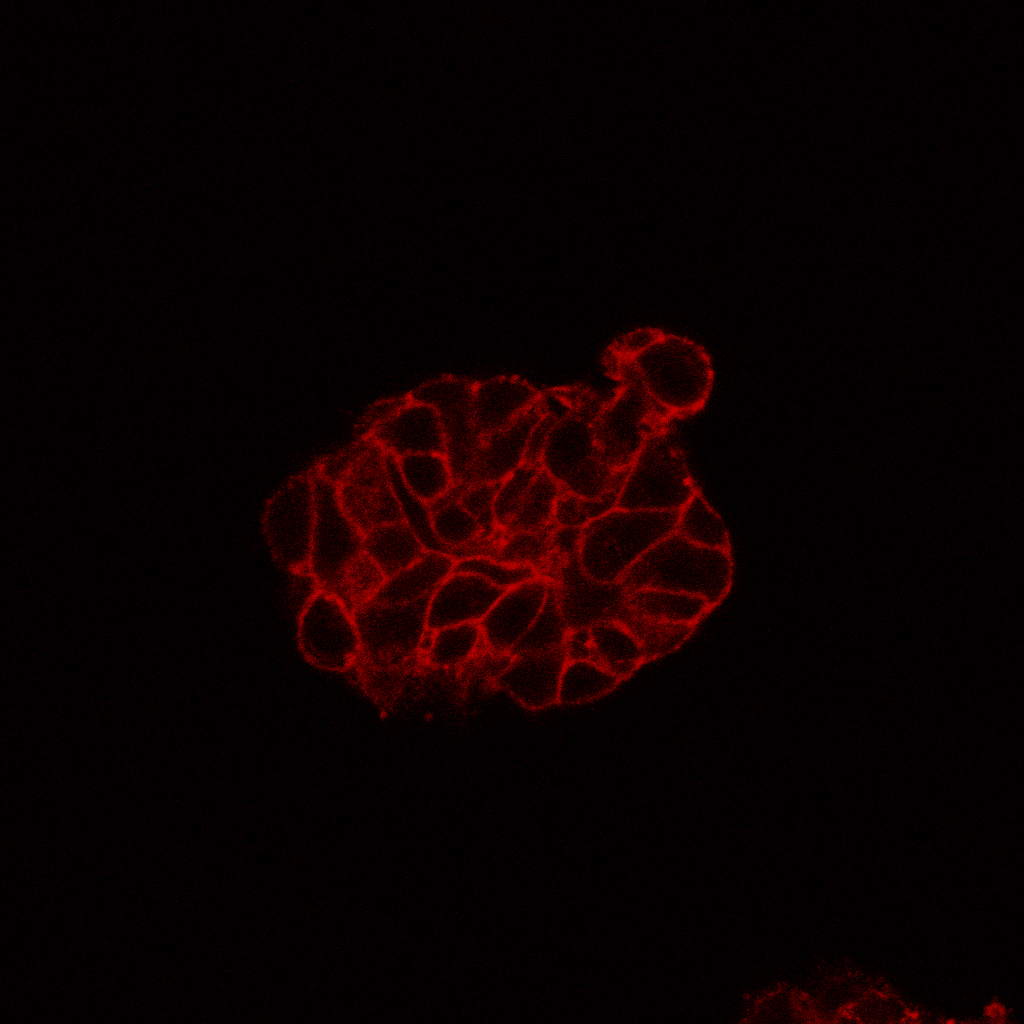

Supplement: Supplementary file 7 — Source Data for Figure 1 [file EMMM-12-e10491-s005.zip › Fig1/Fig_1H_invasive_spheroid_2_(phalloidin).tif]

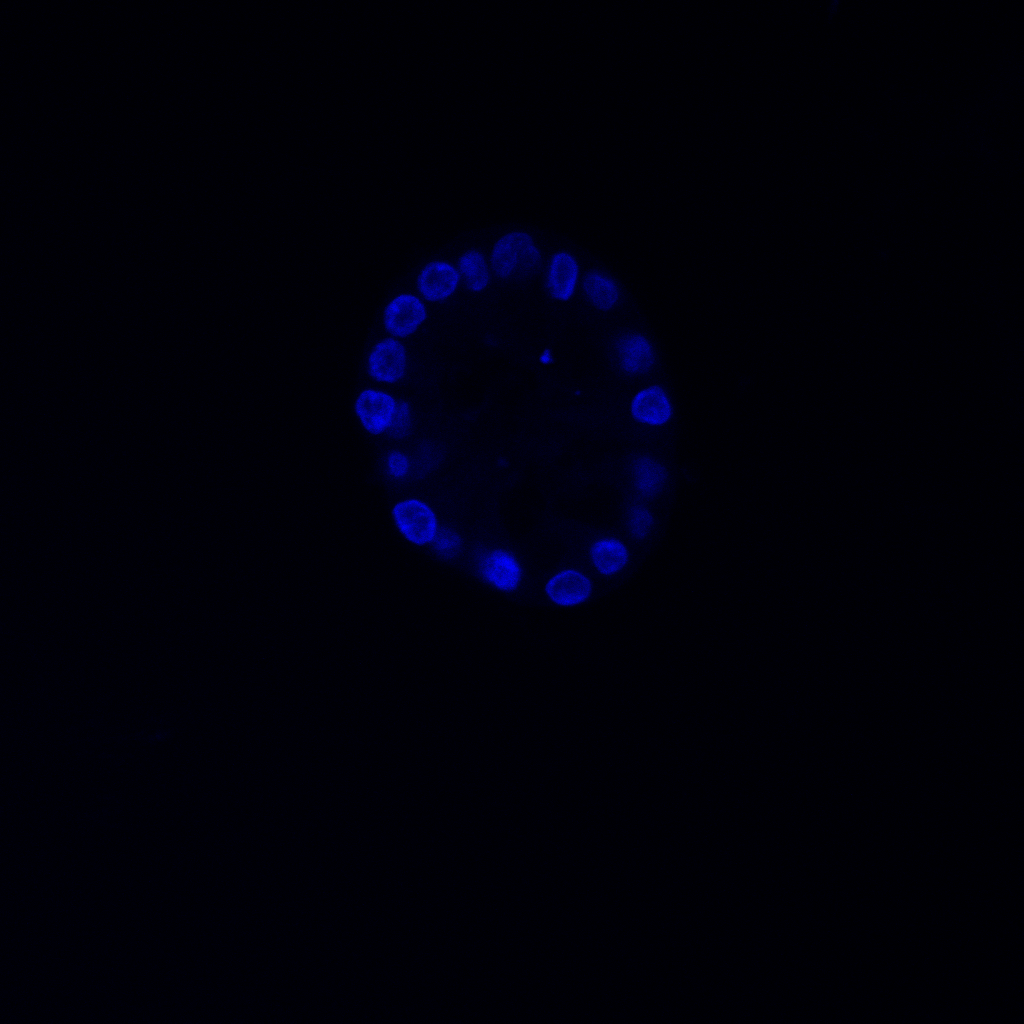

Supplement: Supplementary file 7 — Source Data for Figure 1 [file EMMM-12-e10491-s005.zip › Fig1/Fig_1H_normal_spheroid_(Hoechst_33342).TIF]

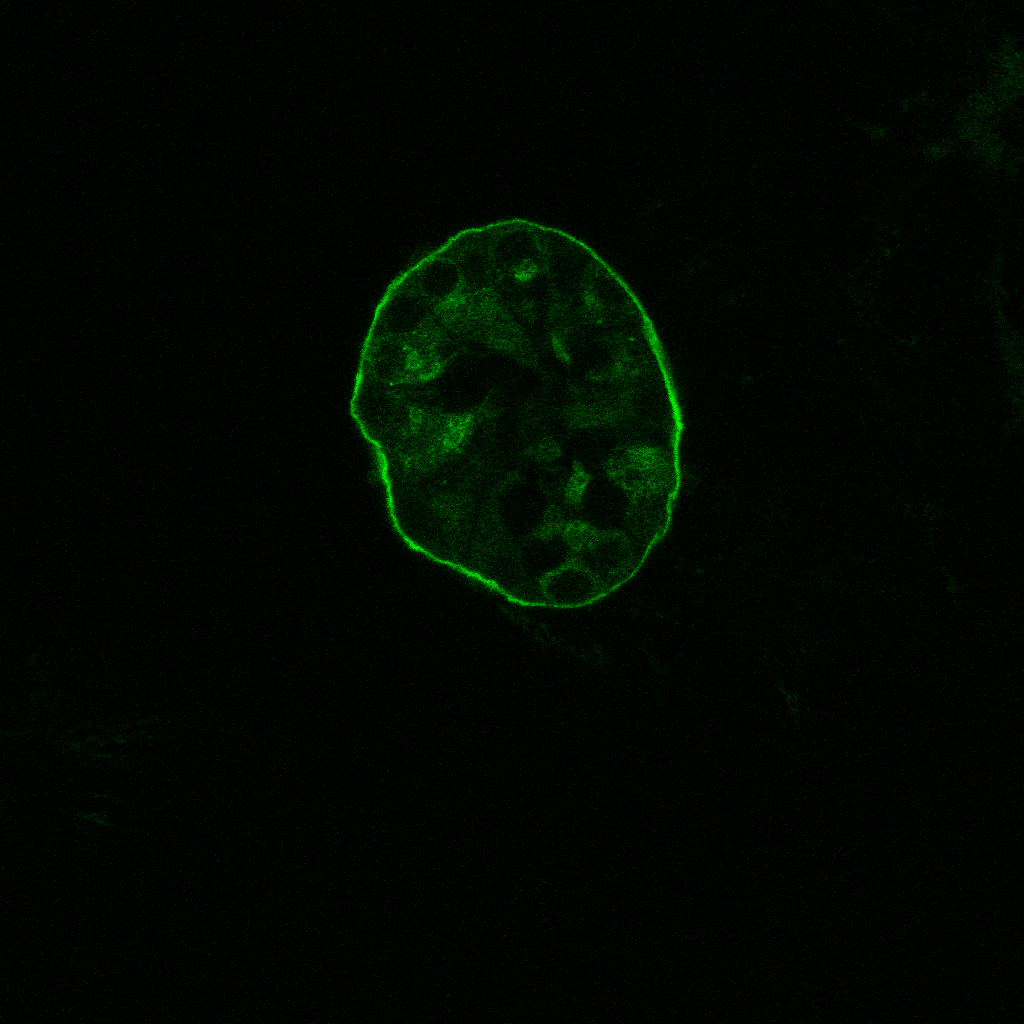

Supplement: Supplementary file 7 — Source Data for Figure 1 [file EMMM-12-e10491-s005.zip › Fig1/Fig_1H_normal_spheroid_(laminin_V).tif]

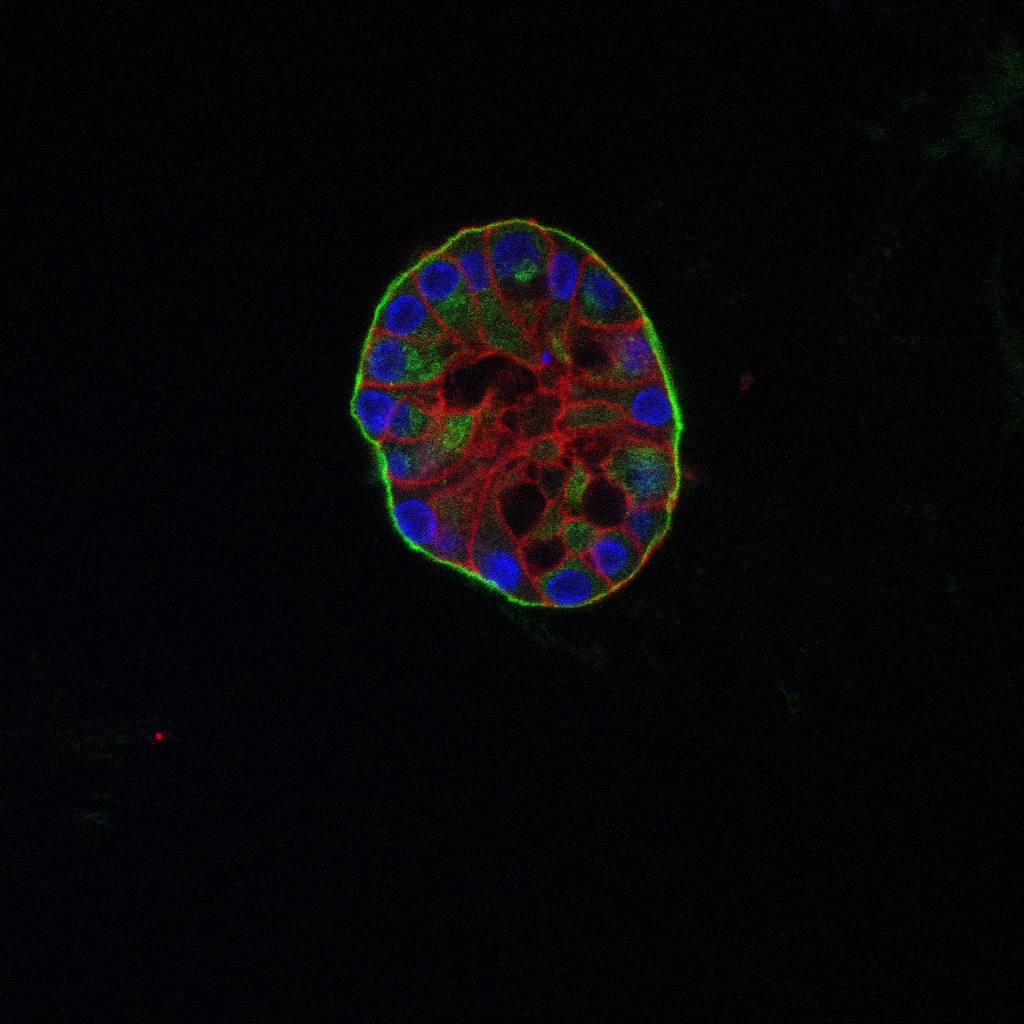

Supplement: Supplementary file 7 — Source Data for Figure 1 [file EMMM-12-e10491-s005.zip › Fig1/Fig_1H_normal_spheroid_(merge).tif]

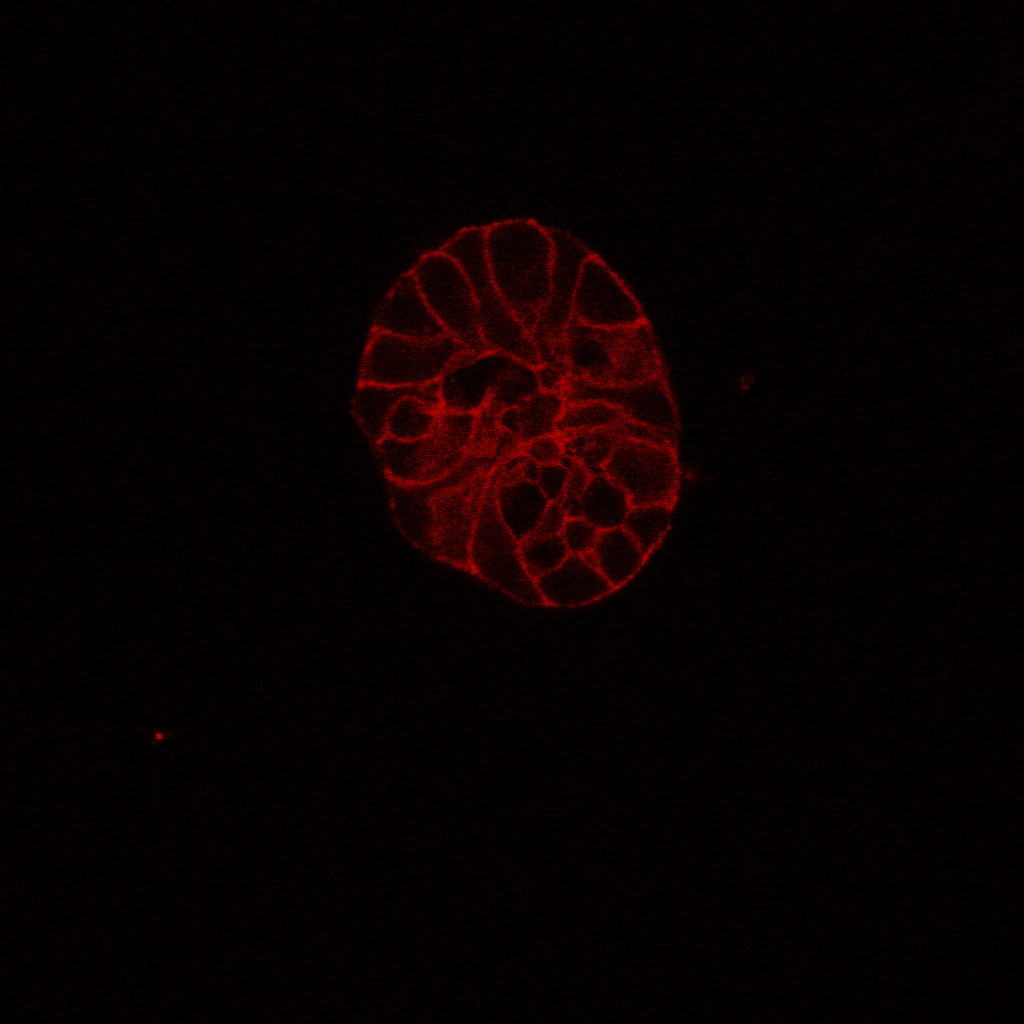

Supplement: Supplementary file 7 — Source Data for Figure 1 [file EMMM-12-e10491-s005.zip › Fig1/Fig_1H_normal_spheroid_(phalloidin).tif]

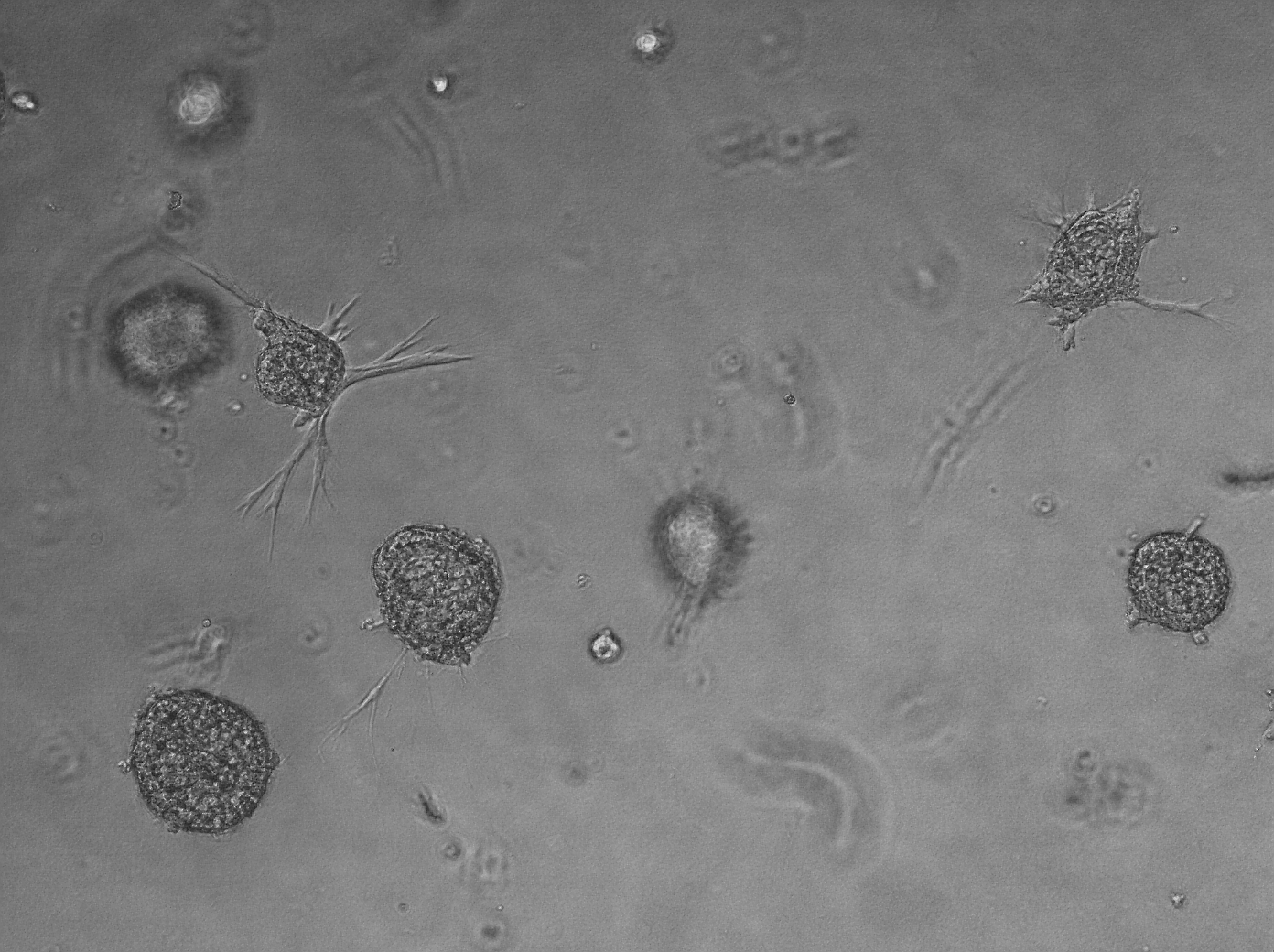

Supplement: Supplementary file 8 — Source Data for Figure 2 [file EMMM-12-e10491-s006.zip › Fig2/Fig_2A_control.TIF]

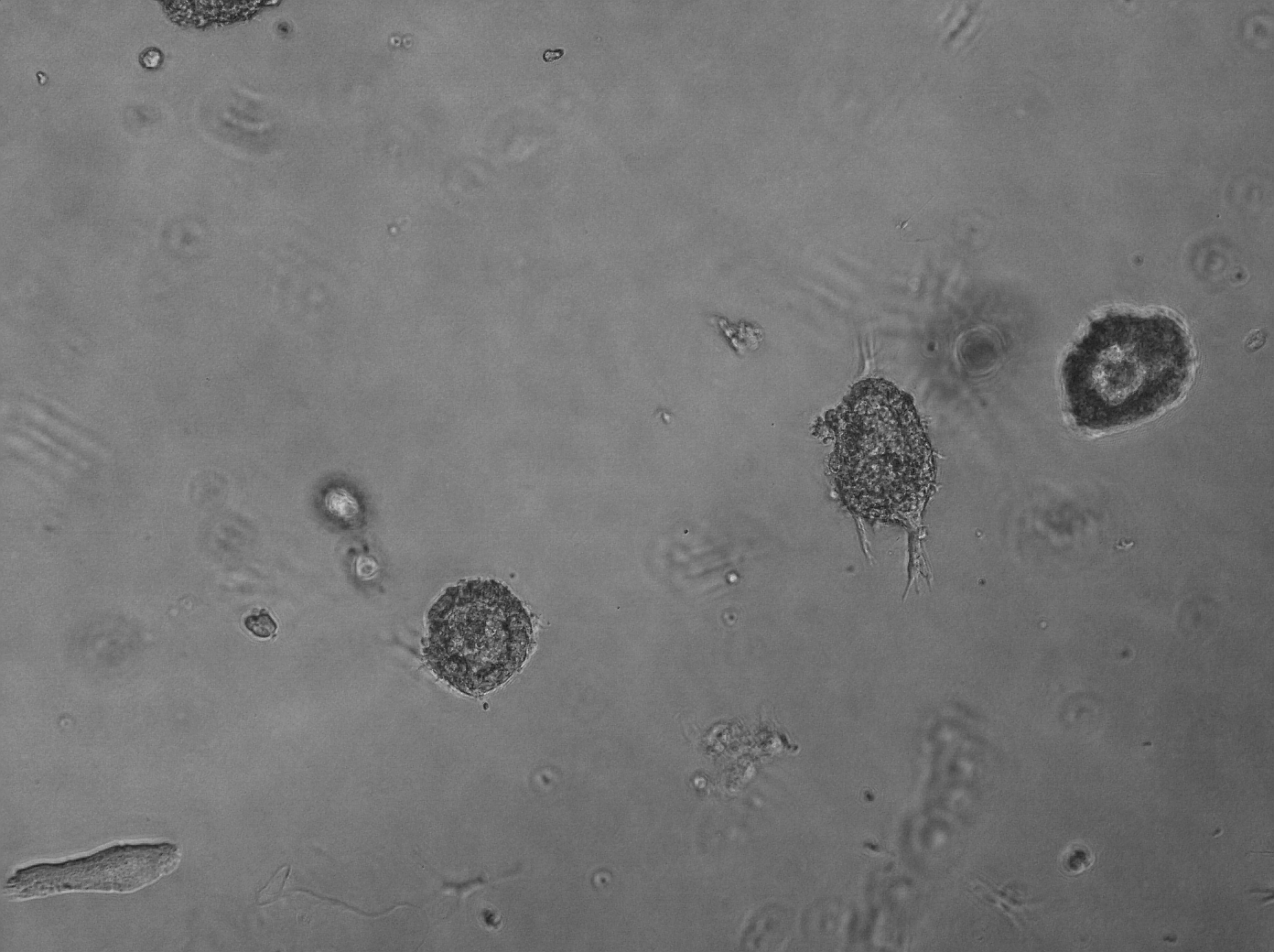

Supplement: Supplementary file 8 — Source Data for Figure 2 [file EMMM-12-e10491-s006.zip › Fig2/Fig_2A_invasive_protrusions.TIF]

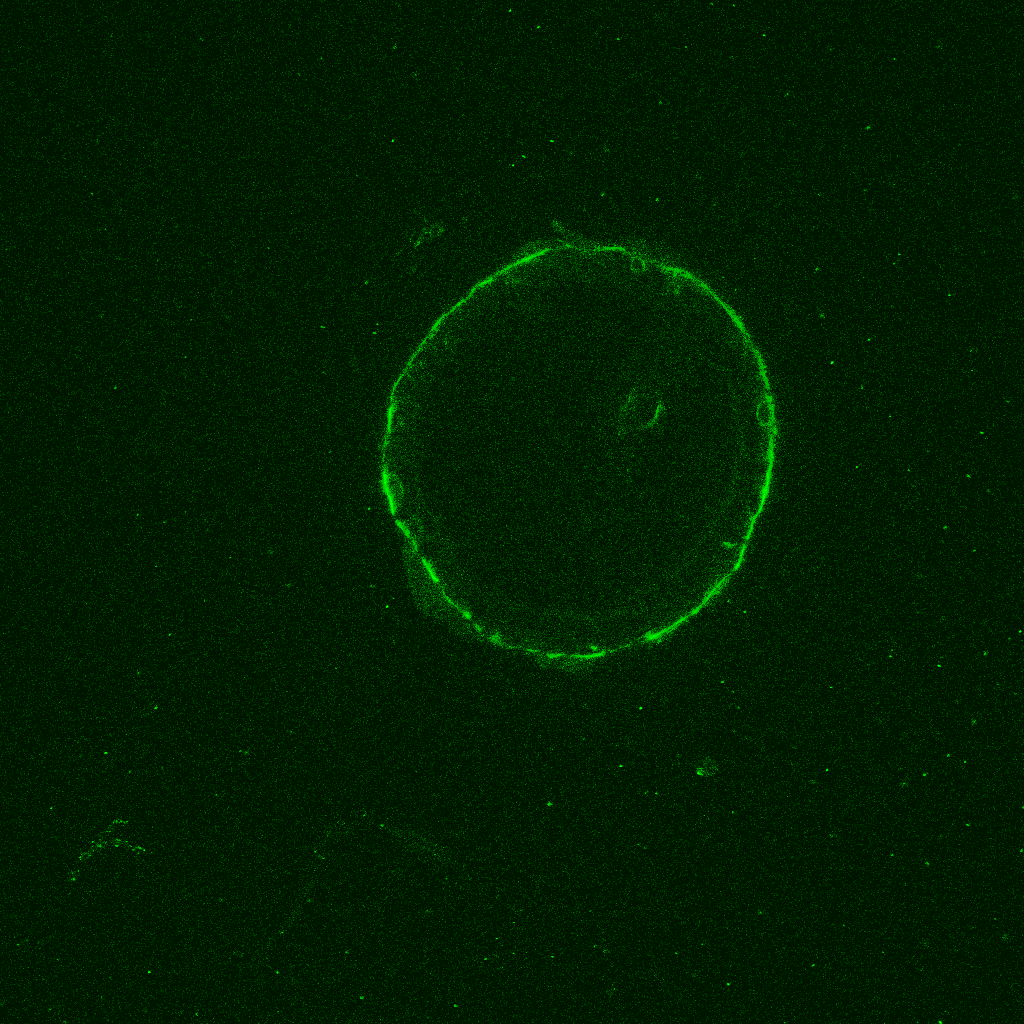

Supplement: Supplementary file 8 — Source Data for Figure 2 [file EMMM-12-e10491-s006.zip › Fig2/Fig_2C_control_(alpha-SMA).TIF]

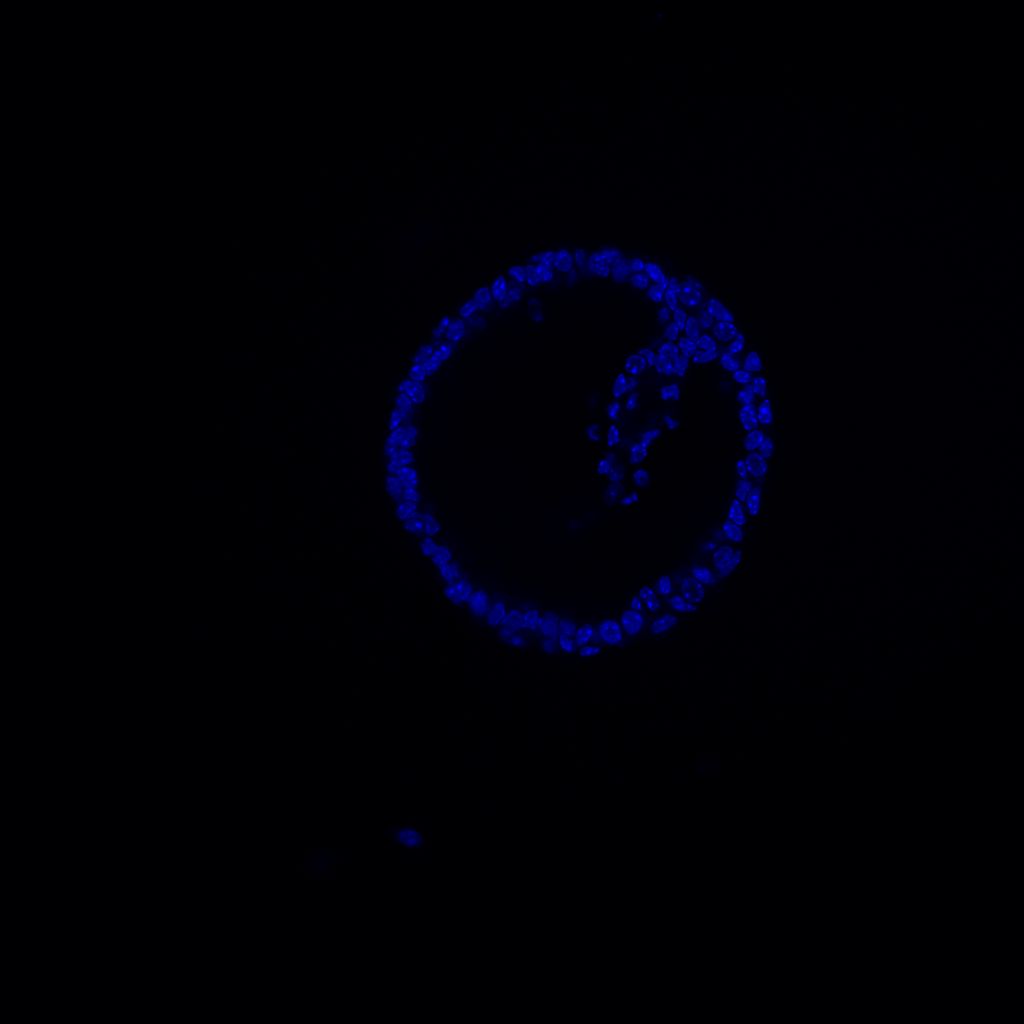

Supplement: Supplementary file 8 — Source Data for Figure 2 [file EMMM-12-e10491-s006.zip › Fig2/Fig_2C_control_(Hoechst_33342).TIF]

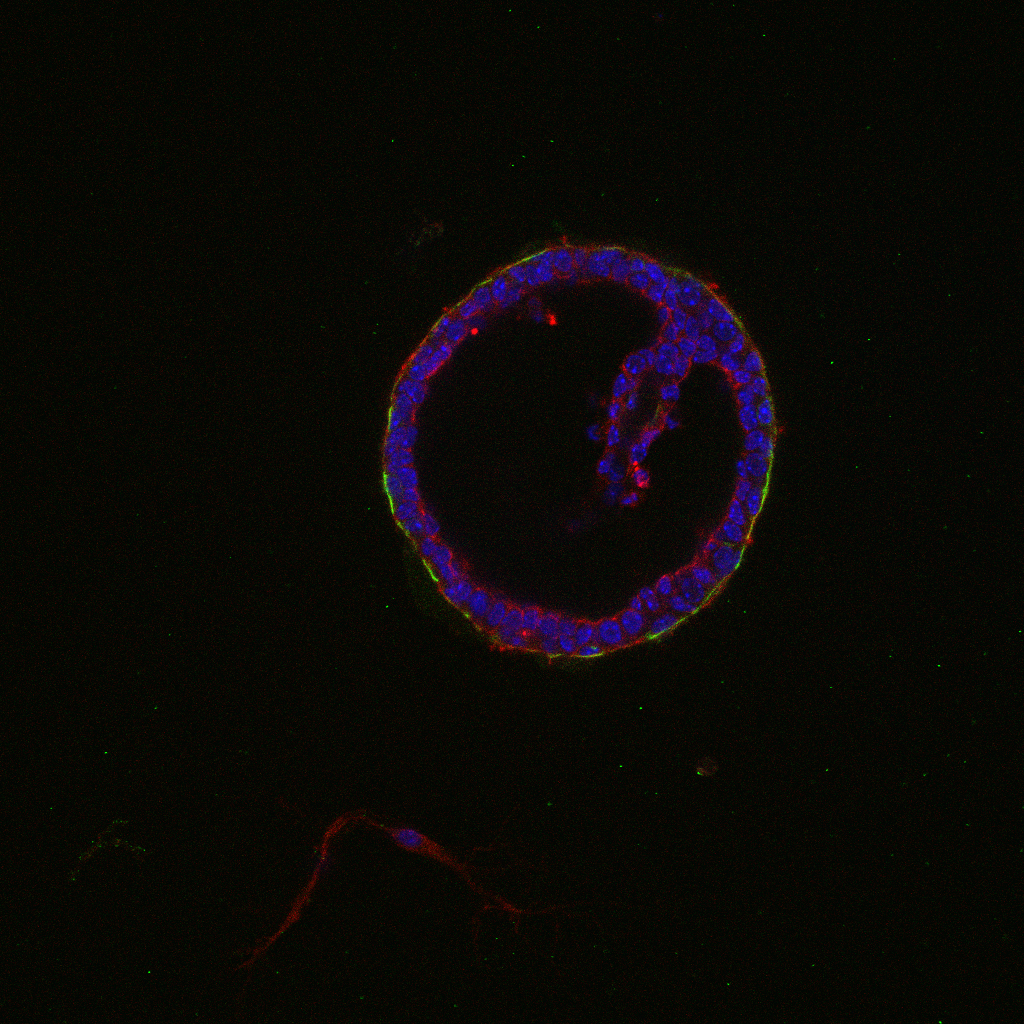

Supplement: Supplementary file 8 — Source Data for Figure 2 [file EMMM-12-e10491-s006.zip › Fig2/Fig_2C_control_(merge).TIF]

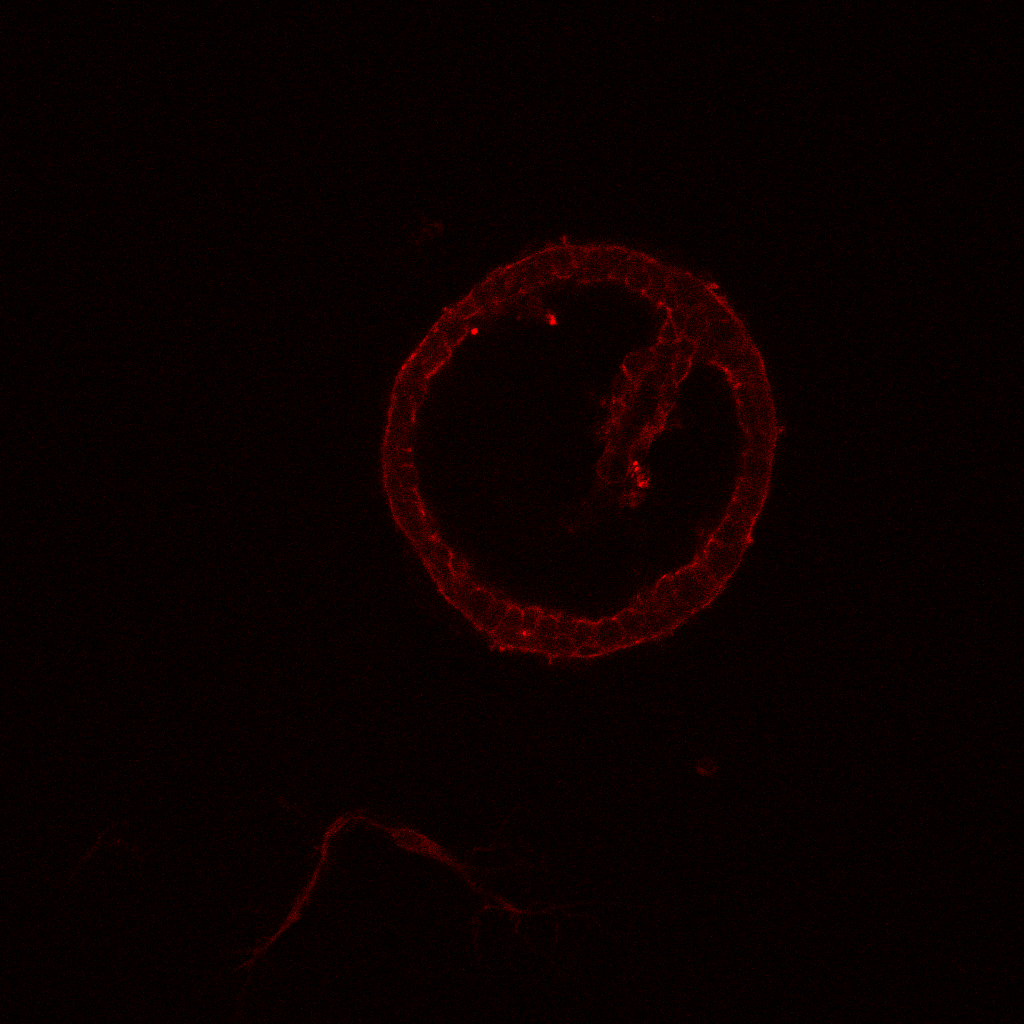

Supplement: Supplementary file 8 — Source Data for Figure 2 [file EMMM-12-e10491-s006.zip › Fig2/Fig_2C_control_(phalloidin).TIF]

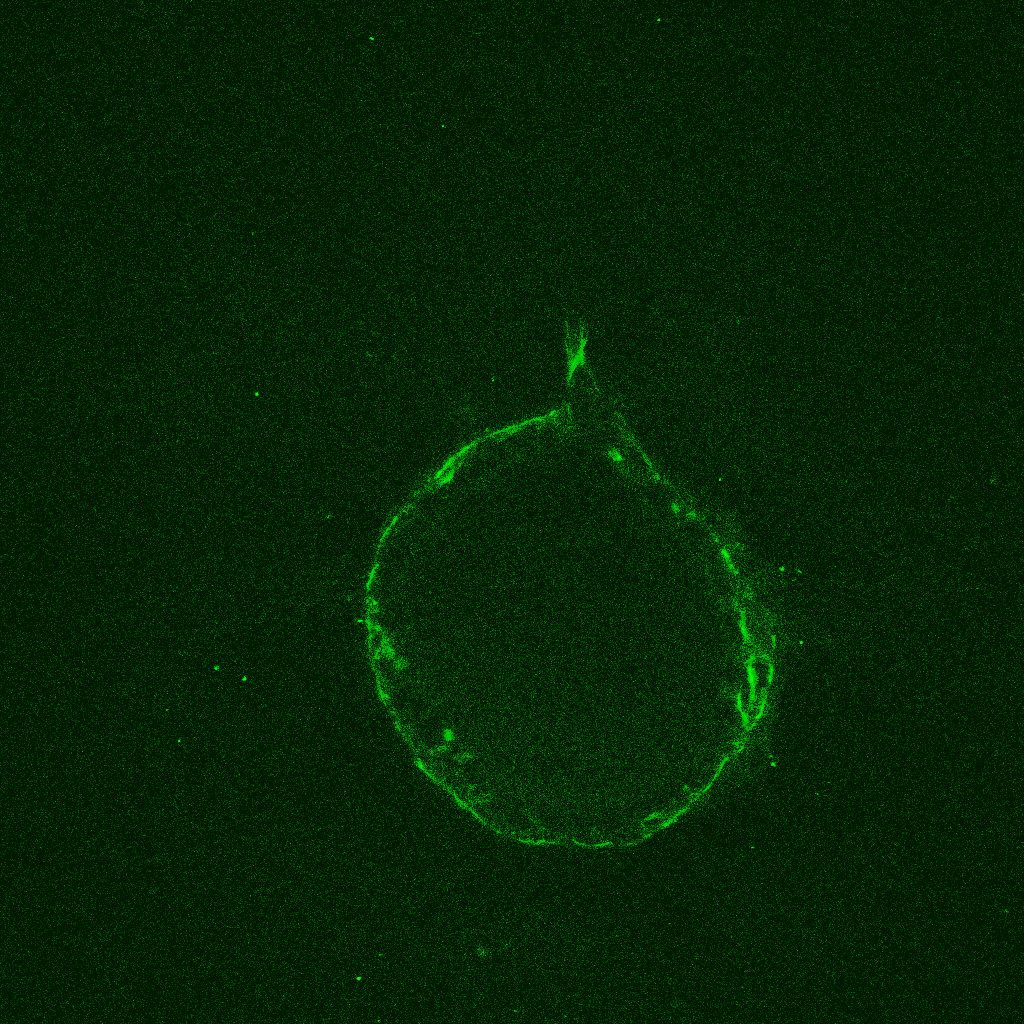

Supplement: Supplementary file 8 — Source Data for Figure 2 [file EMMM-12-e10491-s006.zip › Fig2/Fig_2C_invasive_organoid_(alpha-SMA).TIF]

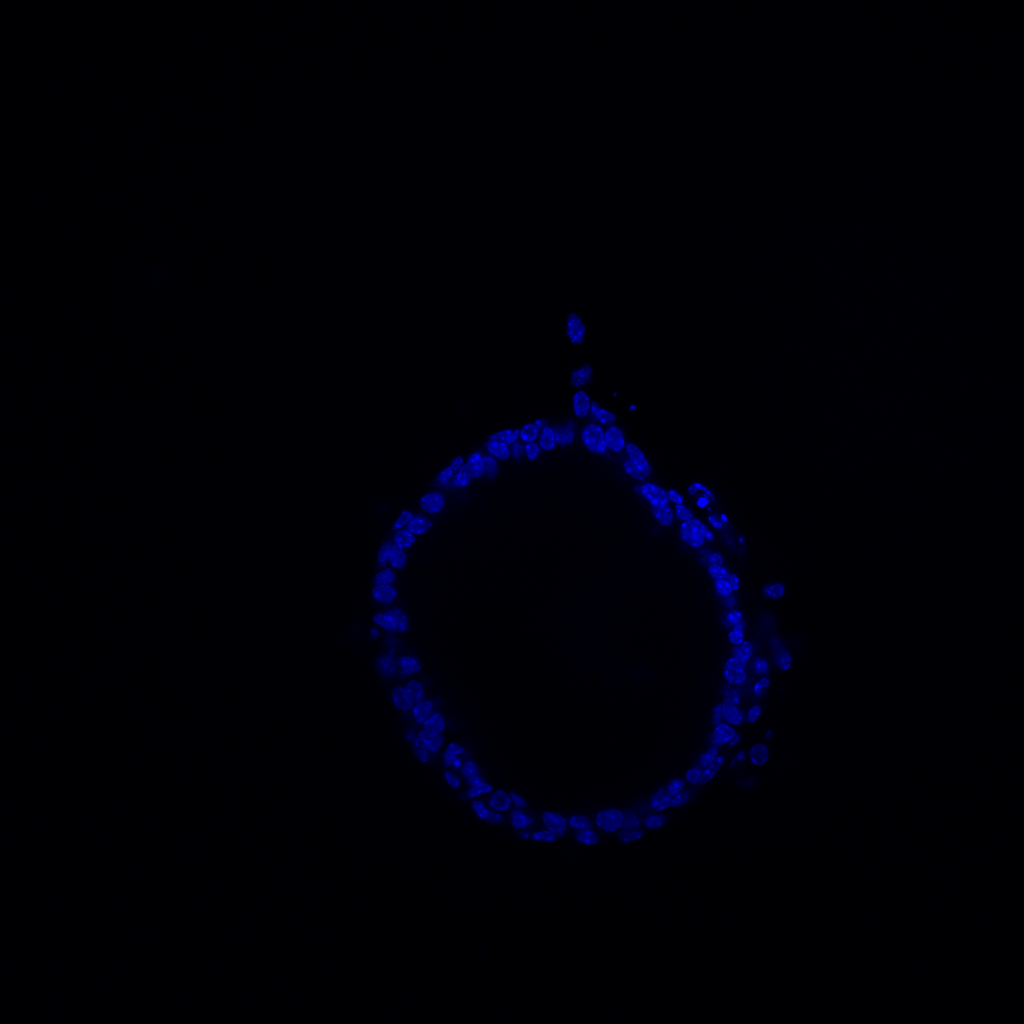

Supplement: Supplementary file 8 — Source Data for Figure 2 [file EMMM-12-e10491-s006.zip › Fig2/Fig_2C_invasive_organoid_(Hoechst_33342).TIF]

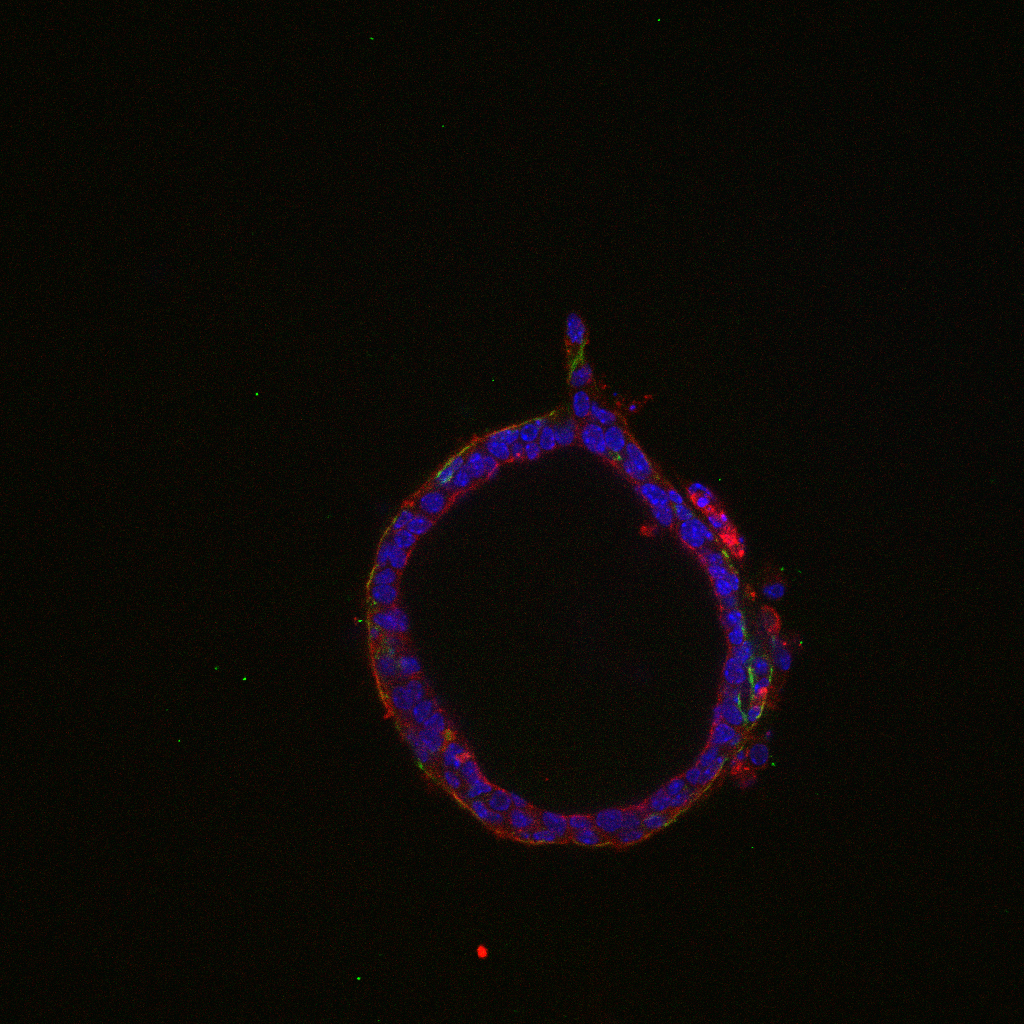

Supplement: Supplementary file 8 — Source Data for Figure 2 [file EMMM-12-e10491-s006.zip › Fig2/Fig_2C_invasive_organoid_(merge).TIF]

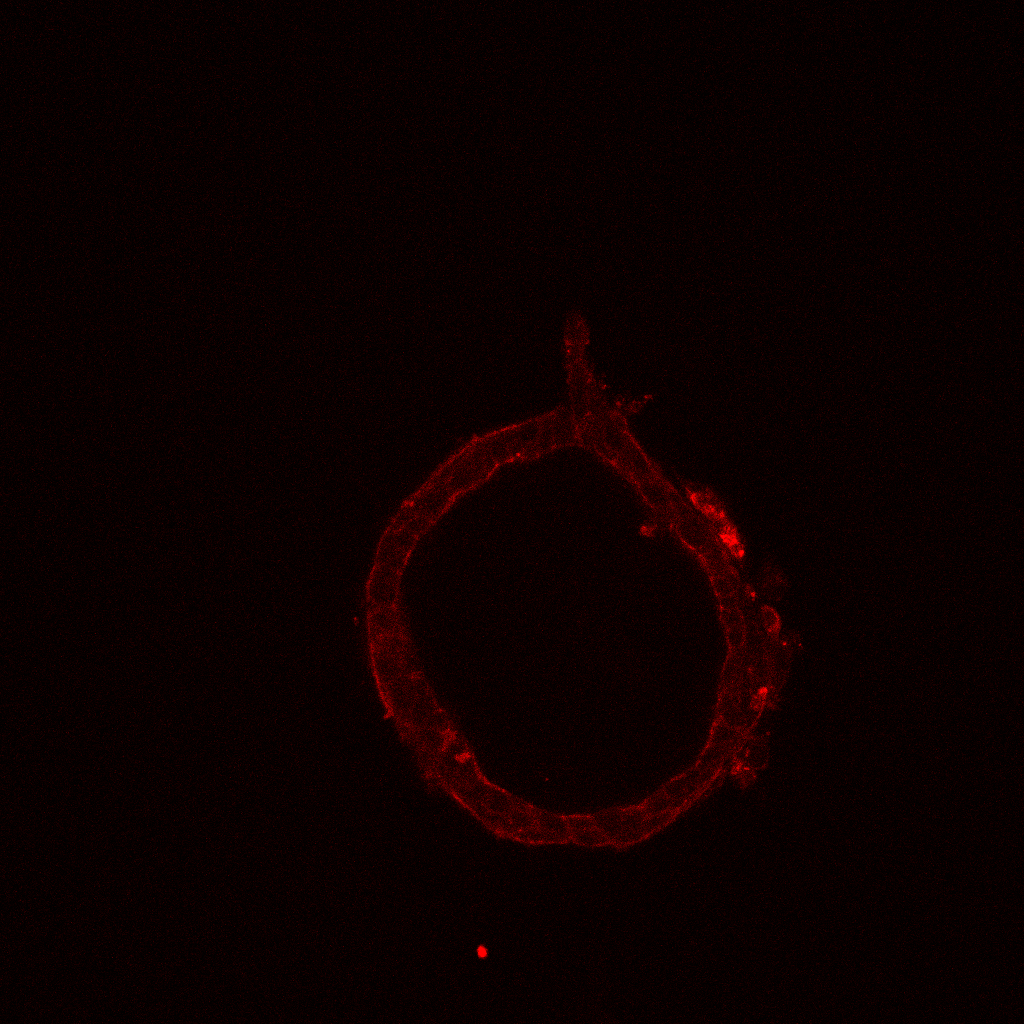

Supplement: Supplementary file 8 — Source Data for Figure 2 [file EMMM-12-e10491-s006.zip › Fig2/Fig_2C_invasive_organoid_(phalloidin).TIF]

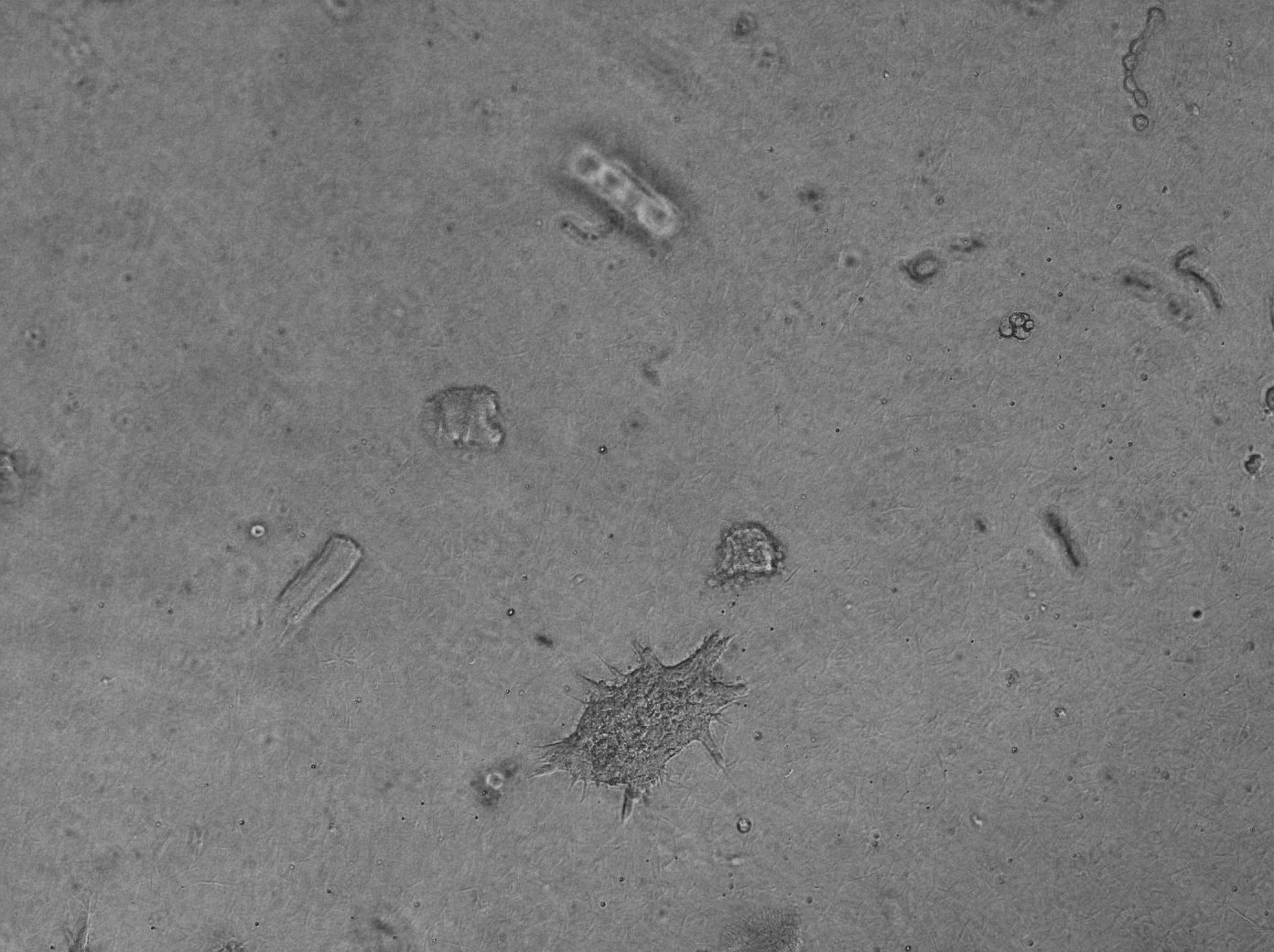

Supplement: Supplementary file 8 — Source Data for Figure 2 [file EMMM-12-e10491-s006.zip › Fig2/Fig_2D_invasive_organoid_HFD.TIF]

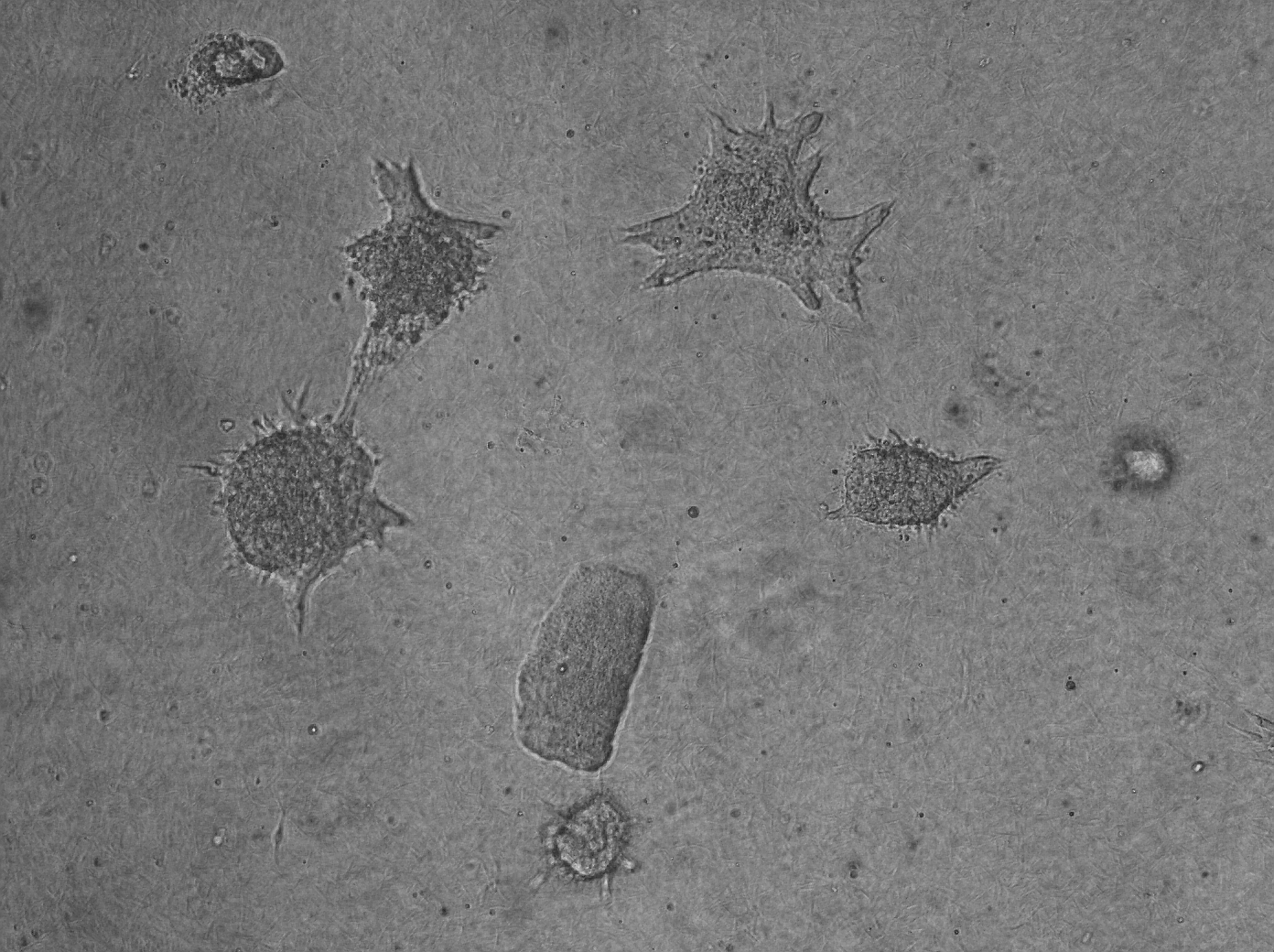

Supplement: Supplementary file 8 — Source Data for Figure 2 [file EMMM-12-e10491-s006.zip › Fig2/Fig_2D_invasive_organoid_ND.TIF]

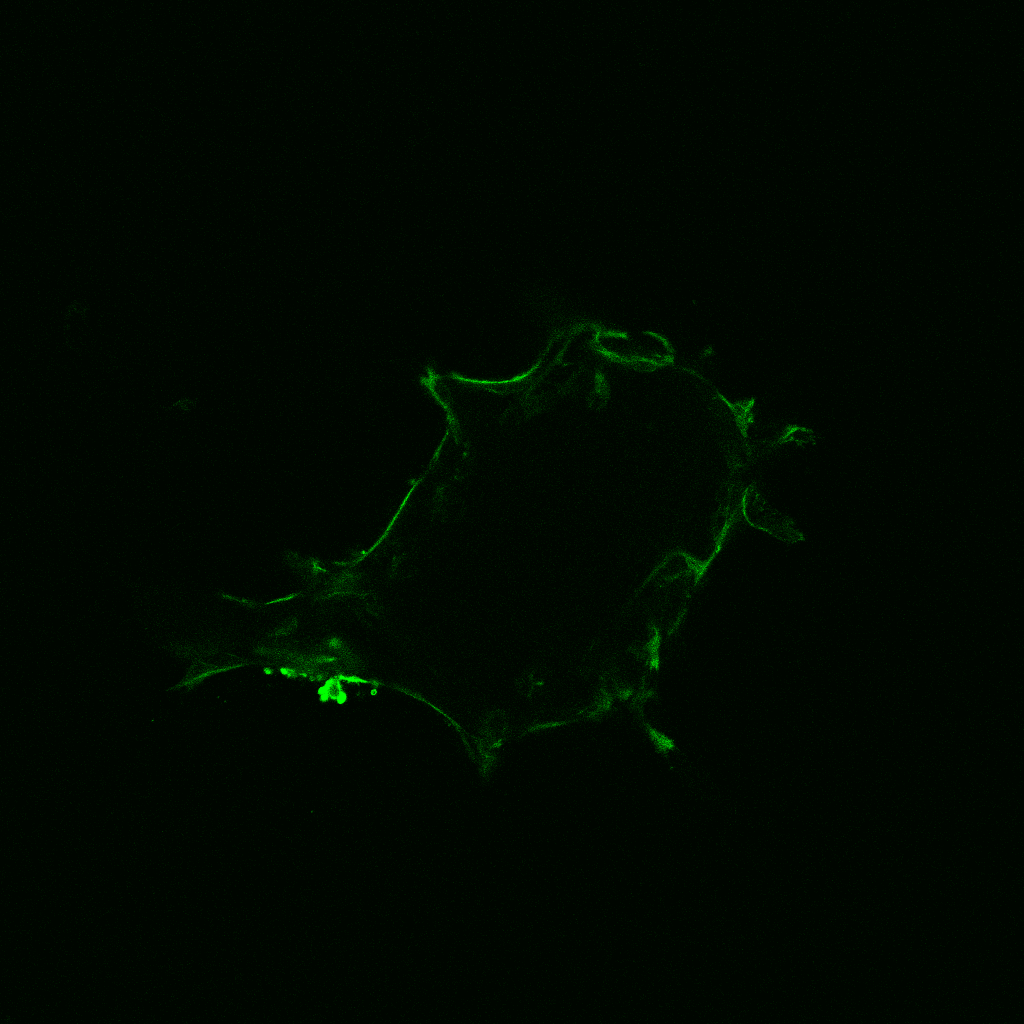

Supplement: Supplementary file 8 — Source Data for Figure 2 [file EMMM-12-e10491-s006.zip › Fig2/Fig_2F_alpha-SMA.TIF]

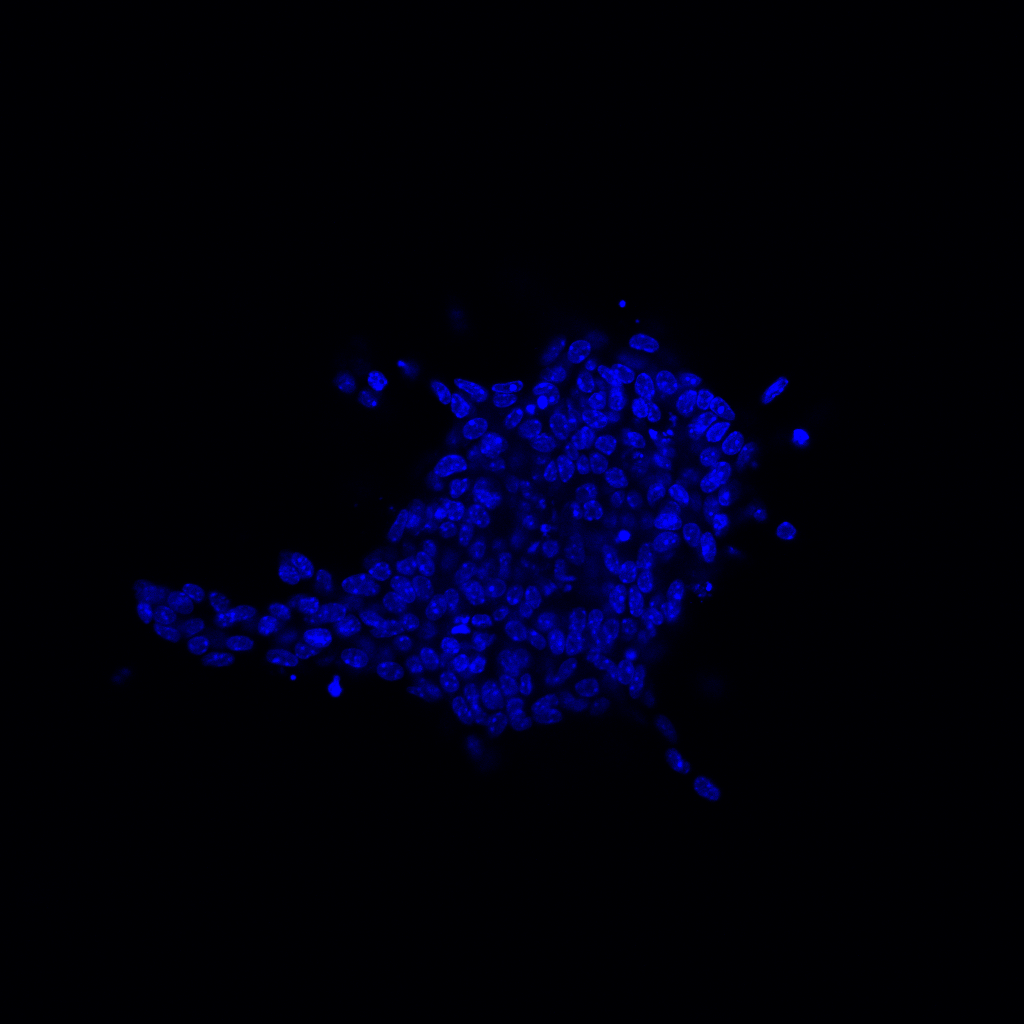

Supplement: Supplementary file 8 — Source Data for Figure 2 [file EMMM-12-e10491-s006.zip › Fig2/Fig_2F_Hoechst_33342.TIF]

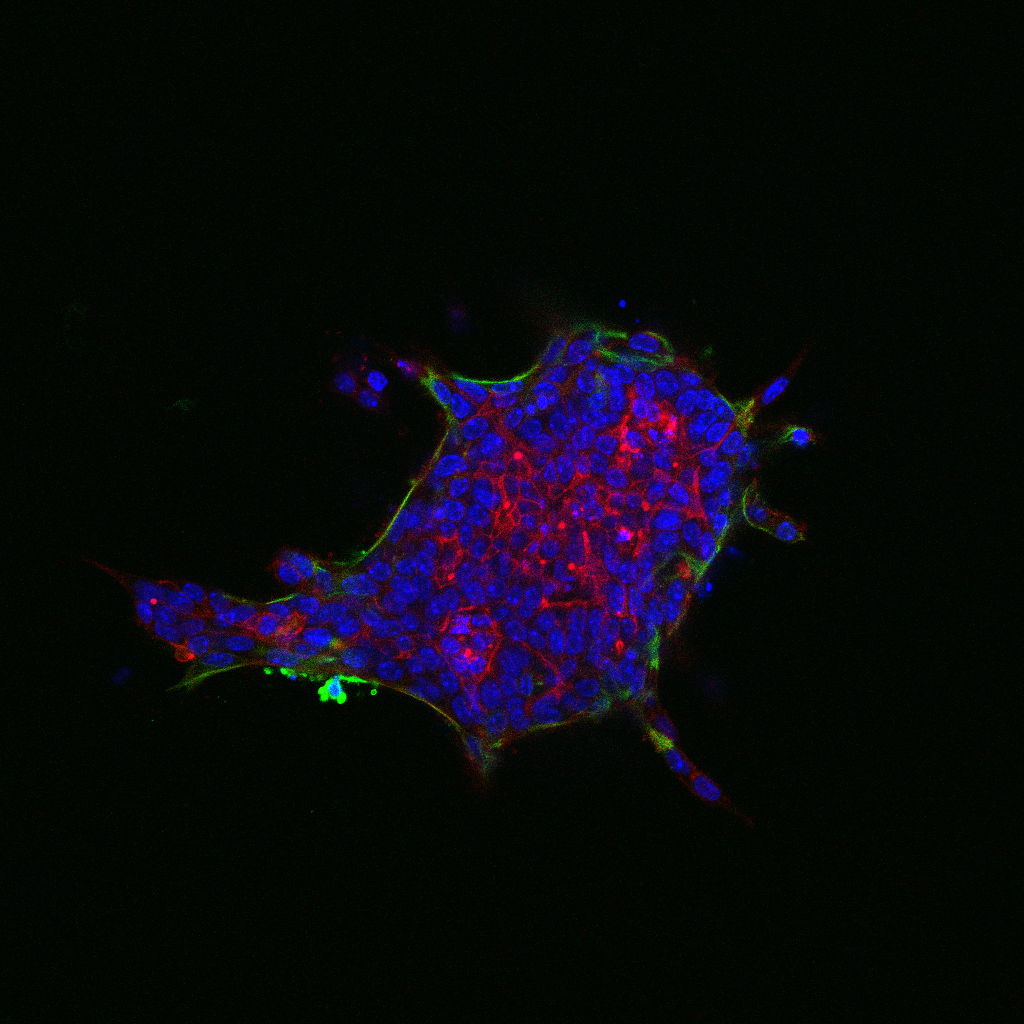

Supplement: Supplementary file 8 — Source Data for Figure 2 [file EMMM-12-e10491-s006.zip › Fig2/Fig_2F_merge.TIF]

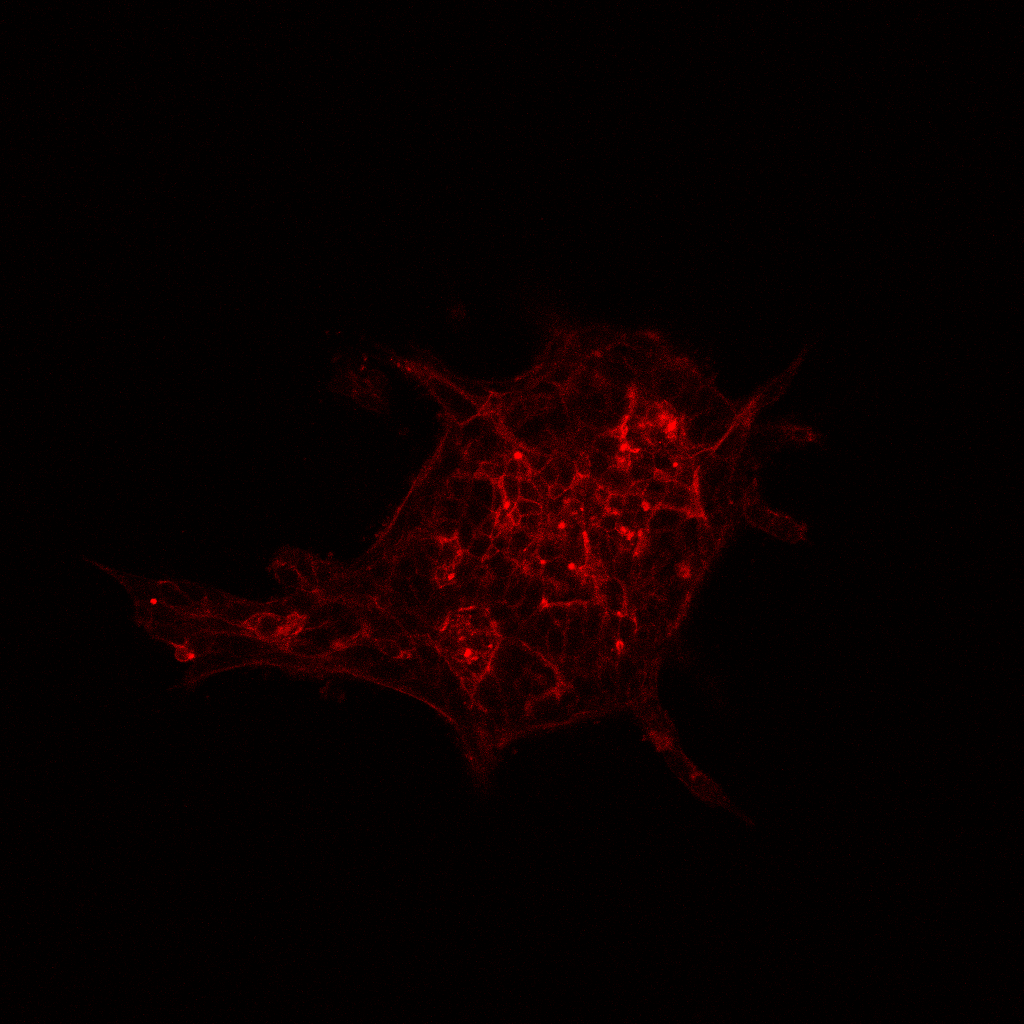

Supplement: Supplementary file 8 — Source Data for Figure 2 [file EMMM-12-e10491-s006.zip › Fig2/Fig_2F_phalloidin.TIF]

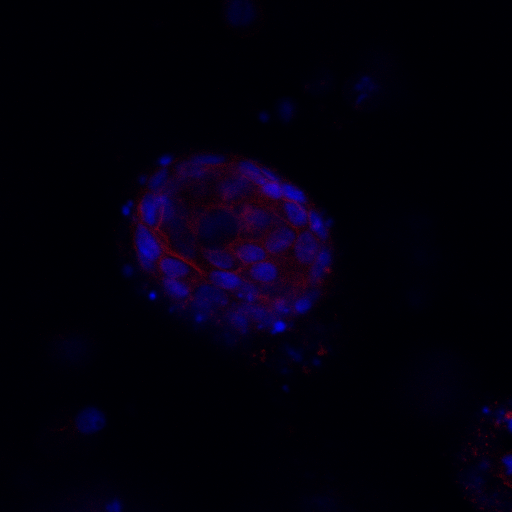

Supplement: Supplementary file 8 — Source Data for Figure 2 [file EMMM-12-e10491-s006.zip › Fig2/Fig_2G_control.TIF]

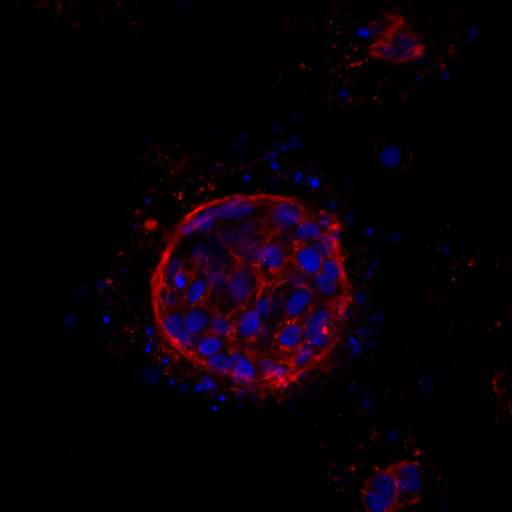

Supplement: Supplementary file 8 — Source Data for Figure 2 [file EMMM-12-e10491-s006.zip › Fig2/Fig_2G_M1A.TIF]

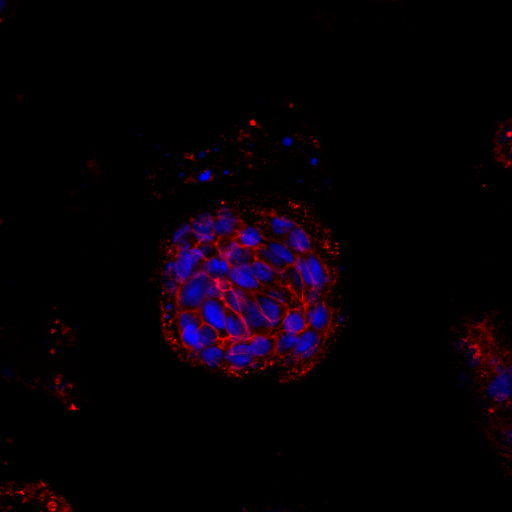

Supplement: Supplementary file 8 — Source Data for Figure 2 [file EMMM-12-e10491-s006.zip › Fig2/Fig_2G_M2A.TIF]

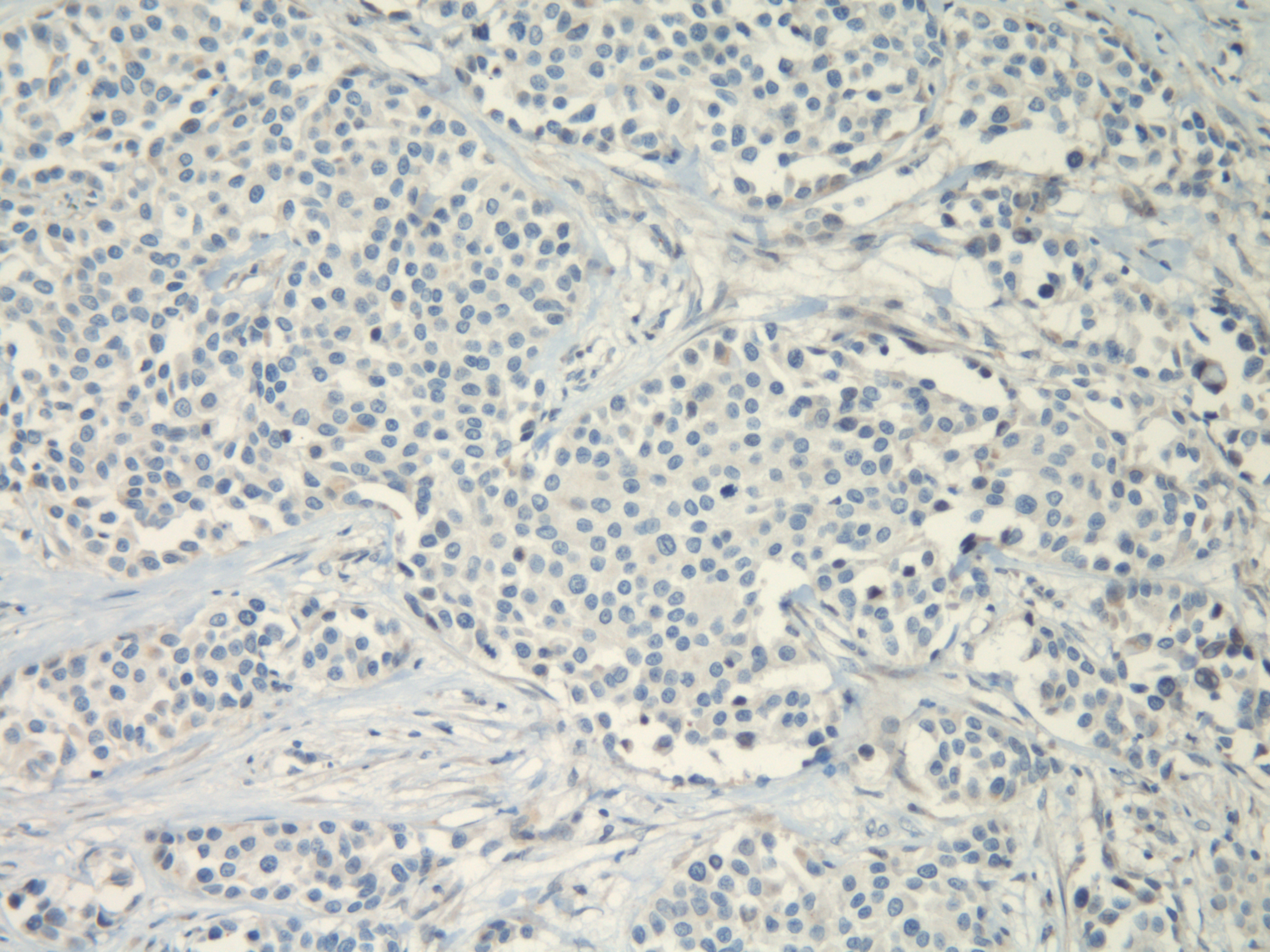

Supplement: Supplementary file 9 — Source Data for Figure 3 [file EMMM-12-e10491-s007.zip › Fig3/Fig_3A_Grade_1.tif]

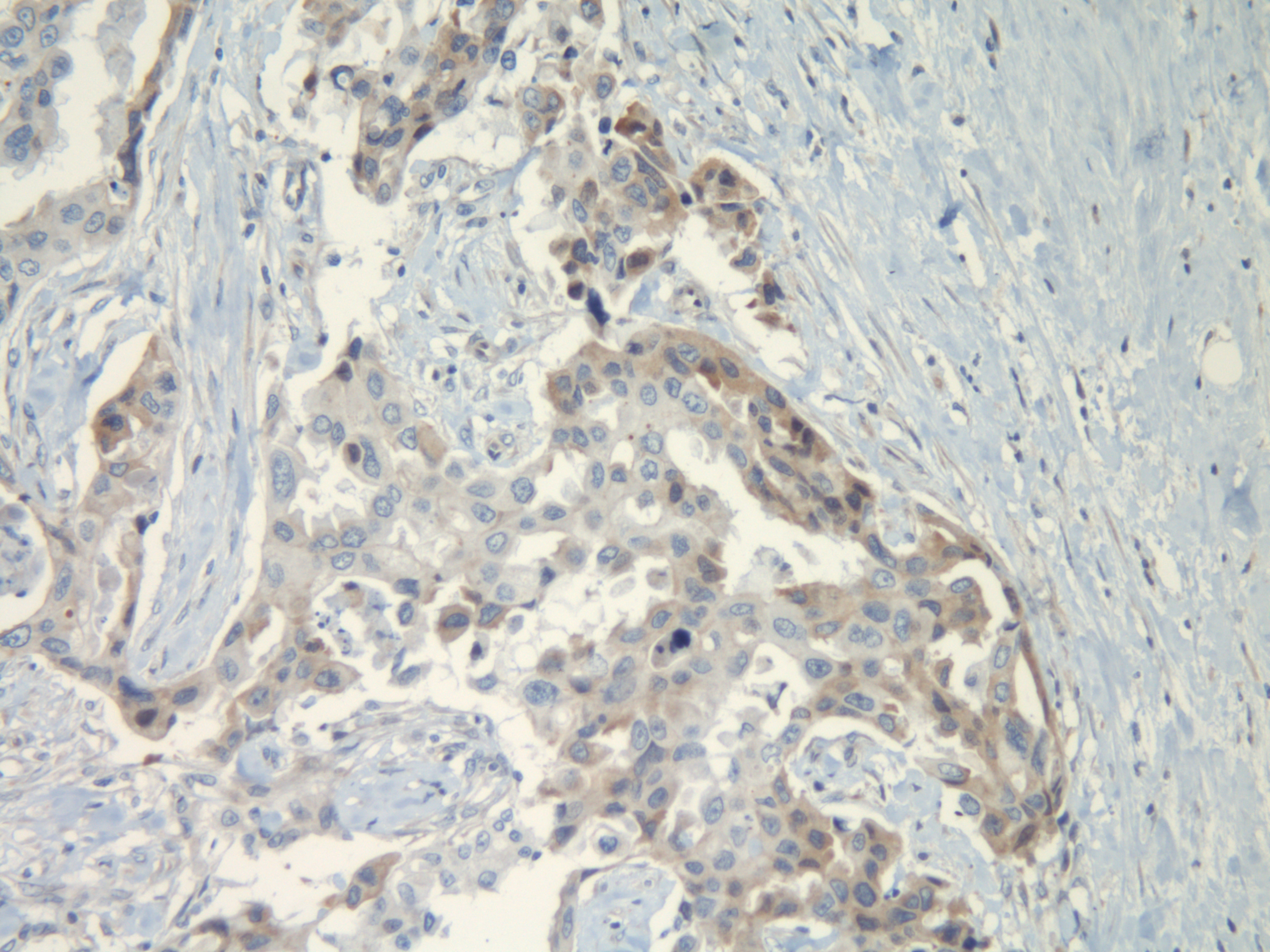

Supplement: Supplementary file 9 — Source Data for Figure 3 [file EMMM-12-e10491-s007.zip › Fig3/Fig_3A_Grade_2.tif]

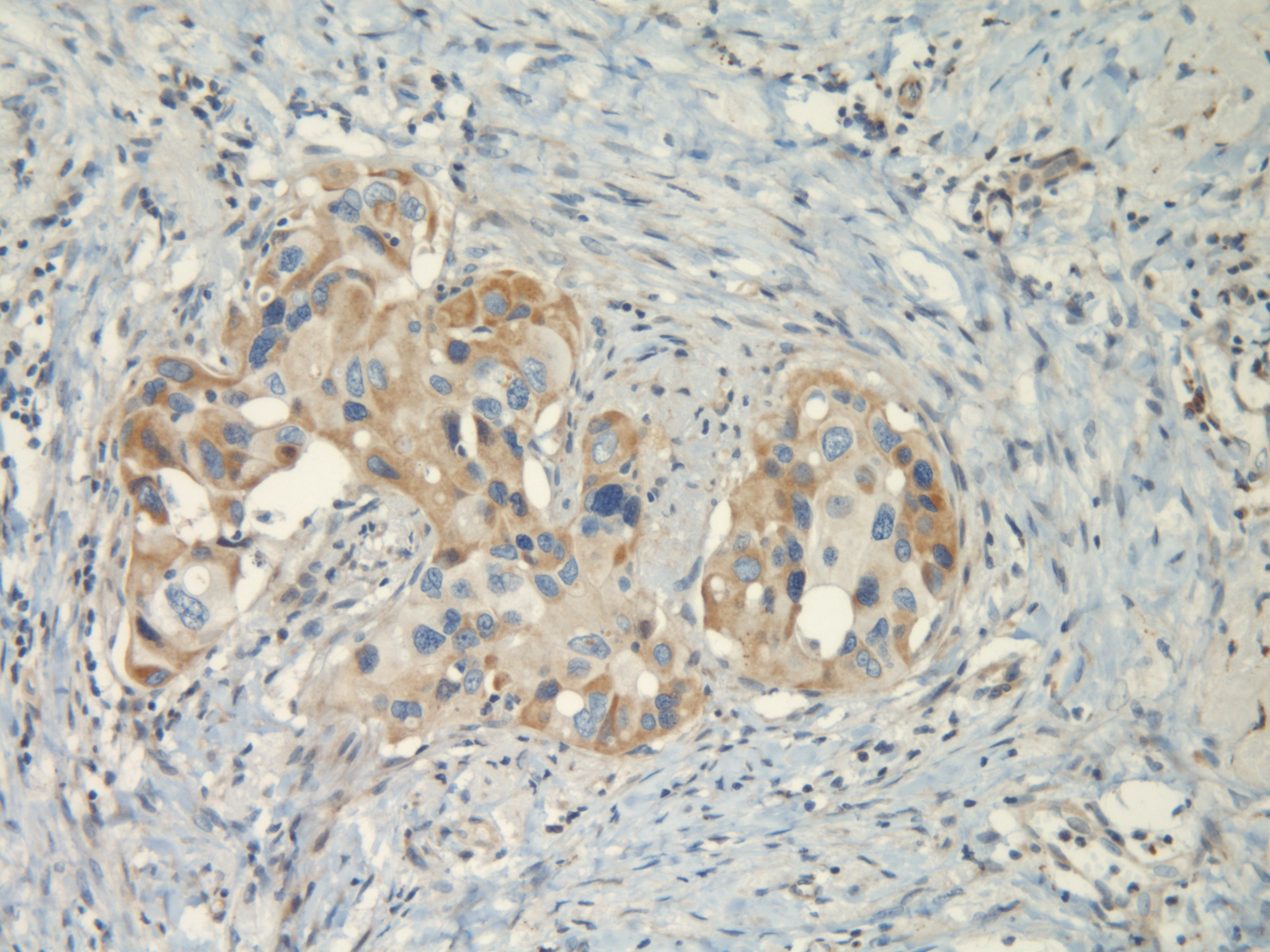

Supplement: Supplementary file 9 — Source Data for Figure 3 [file EMMM-12-e10491-s007.zip › Fig3/Fig_3A_Grade_3.tif]

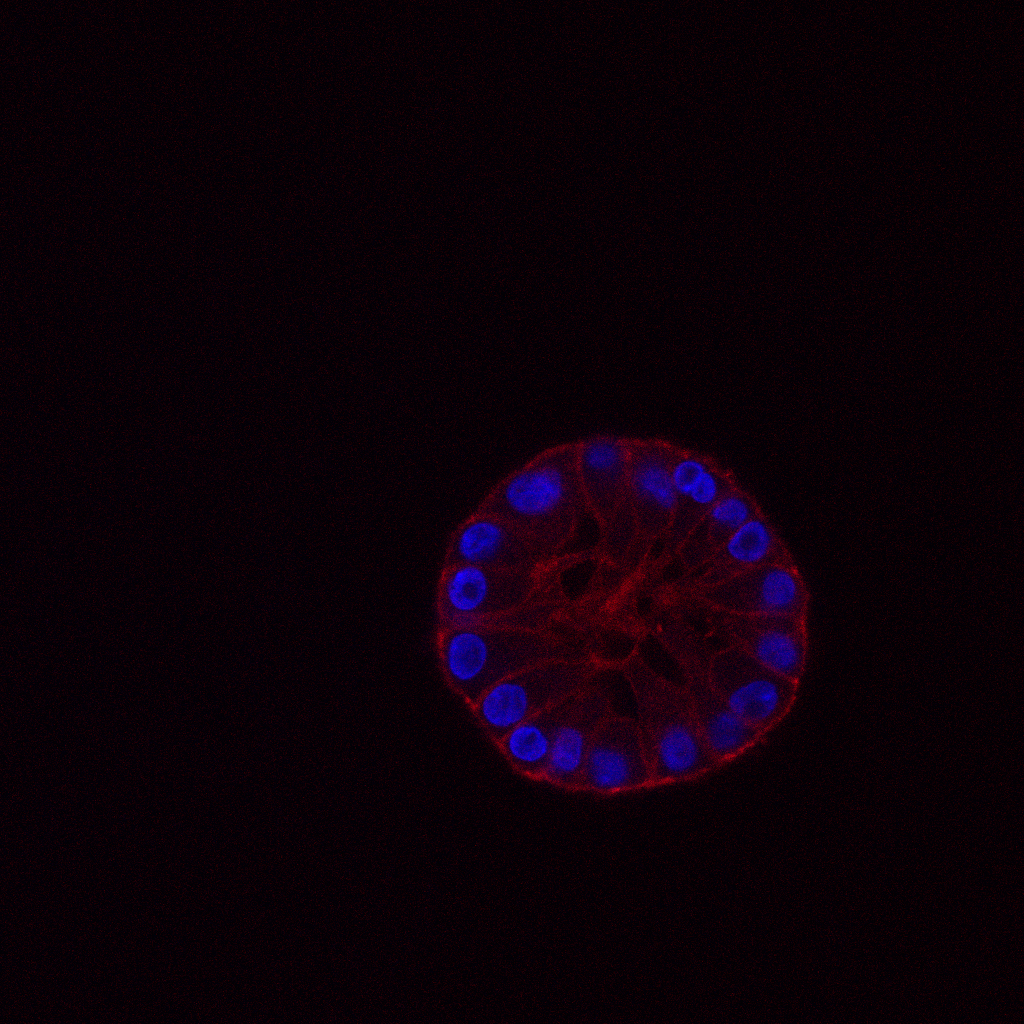

Supplement: Supplementary file 10 — Source Data for Figure 4 [file EMMM-12-e10491-s008.zip › Fig4/Fig_4B_control.TIF]

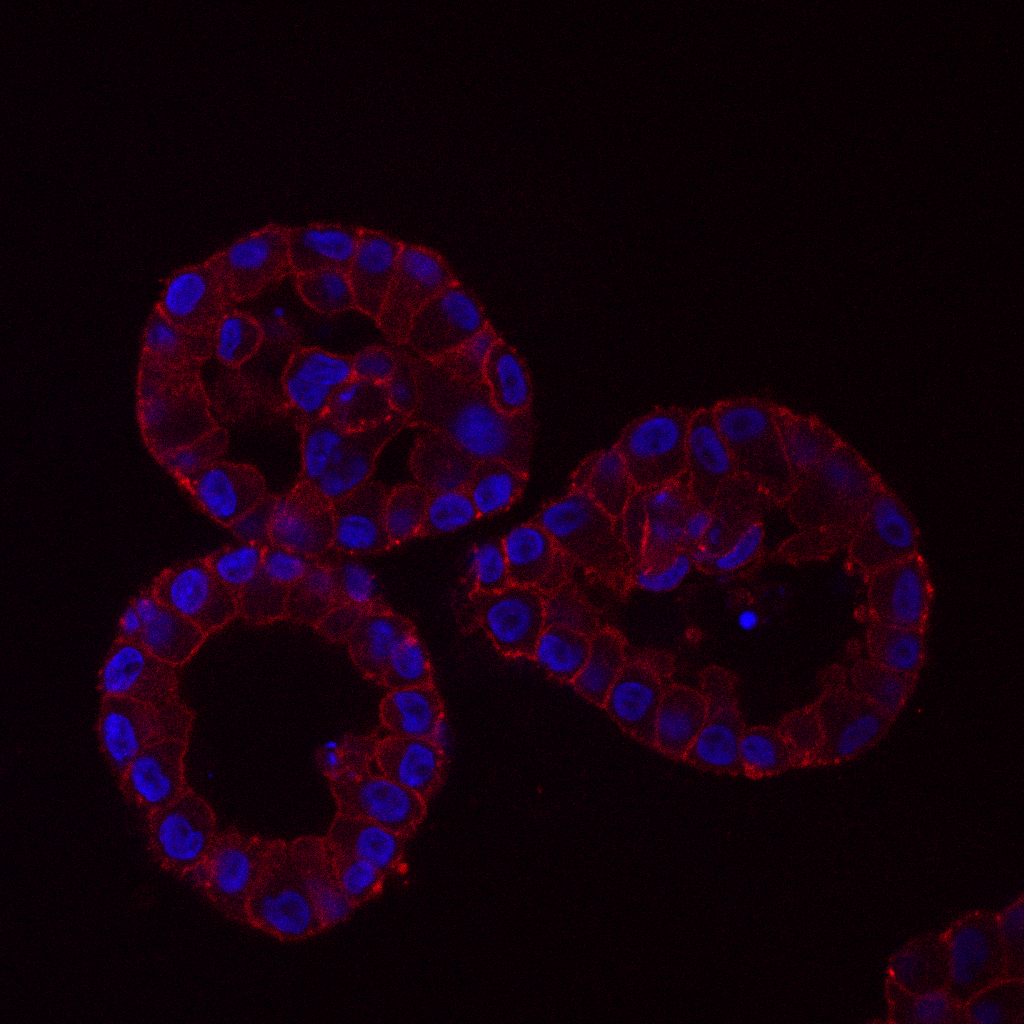

Supplement: Supplementary file 10 — Source Data for Figure 4 [file EMMM-12-e10491-s008.zip › Fig4/Fig_4B_control_+_amlexanox.TIF]

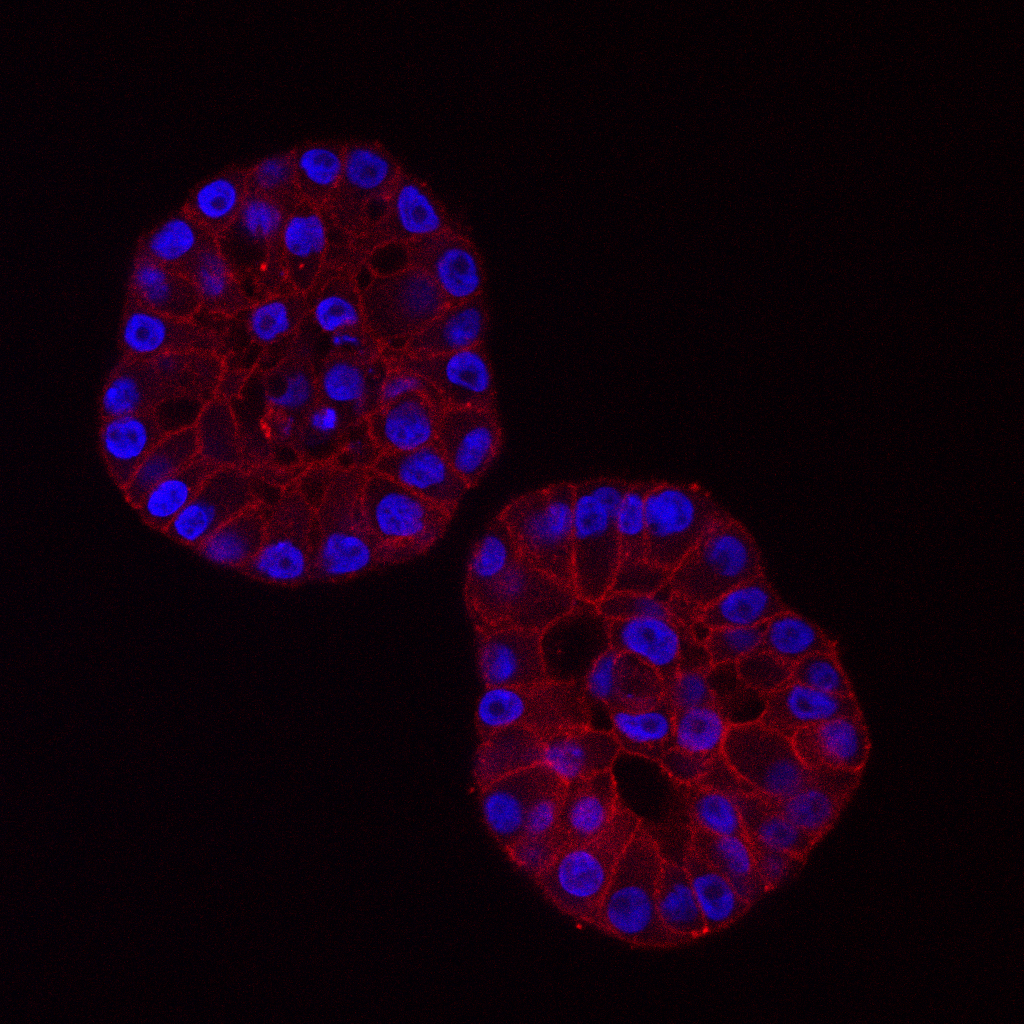

Supplement: Supplementary file 10 — Source Data for Figure 4 [file EMMM-12-e10491-s008.zip › Fig4/Fig_4B_M1A.TIF]

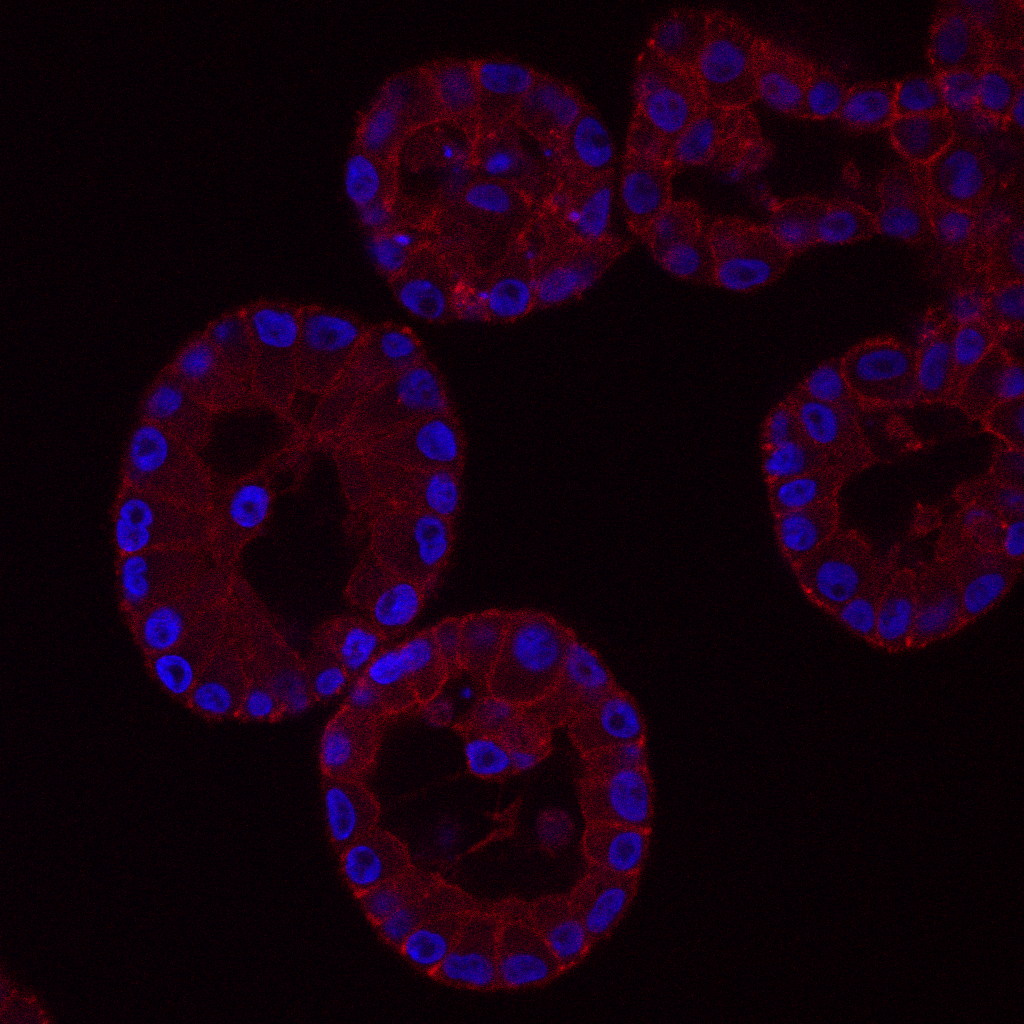

Supplement: Supplementary file 10 — Source Data for Figure 4 [file EMMM-12-e10491-s008.zip › Fig4/Fig_4B_M1A_+_amlexanox.TIF]

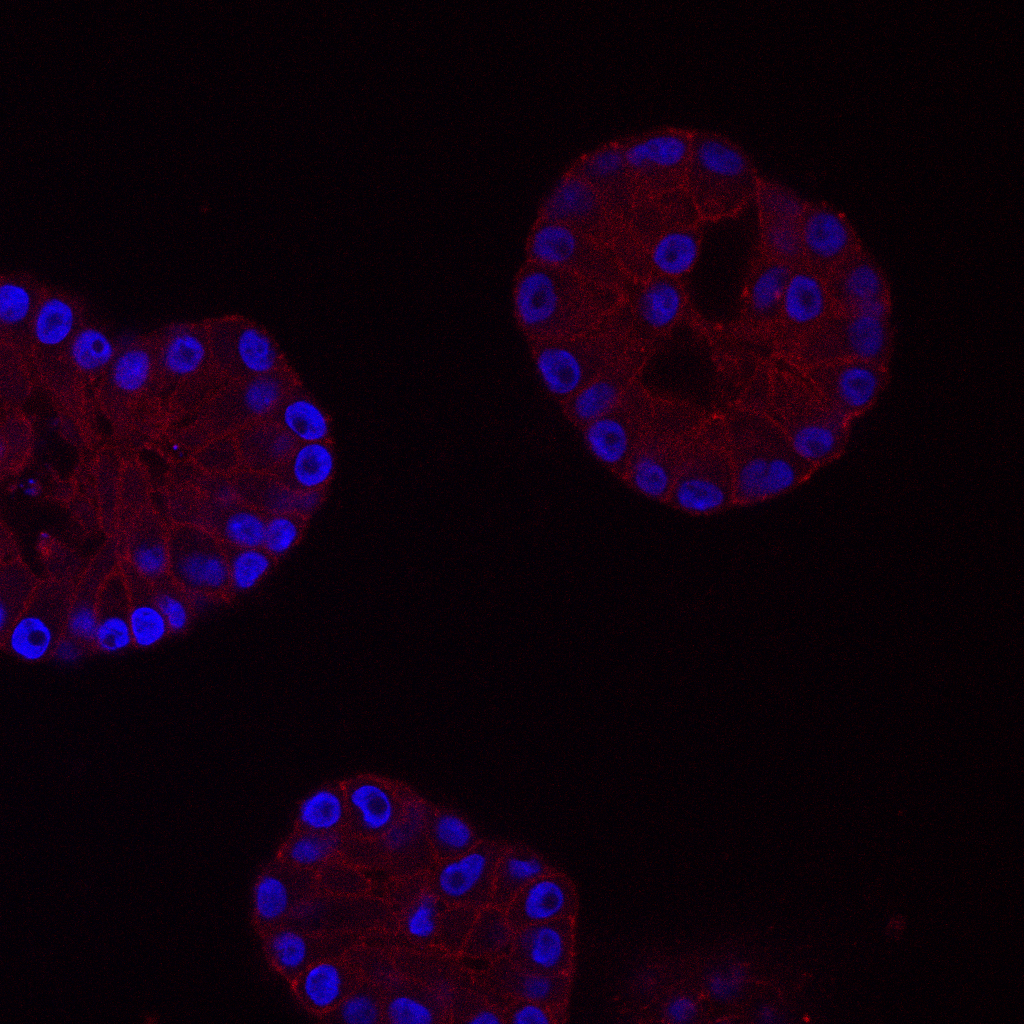

Supplement: Supplementary file 10 — Source Data for Figure 4 [file EMMM-12-e10491-s008.zip › Fig4/Fig_4B_M1D.TIF]

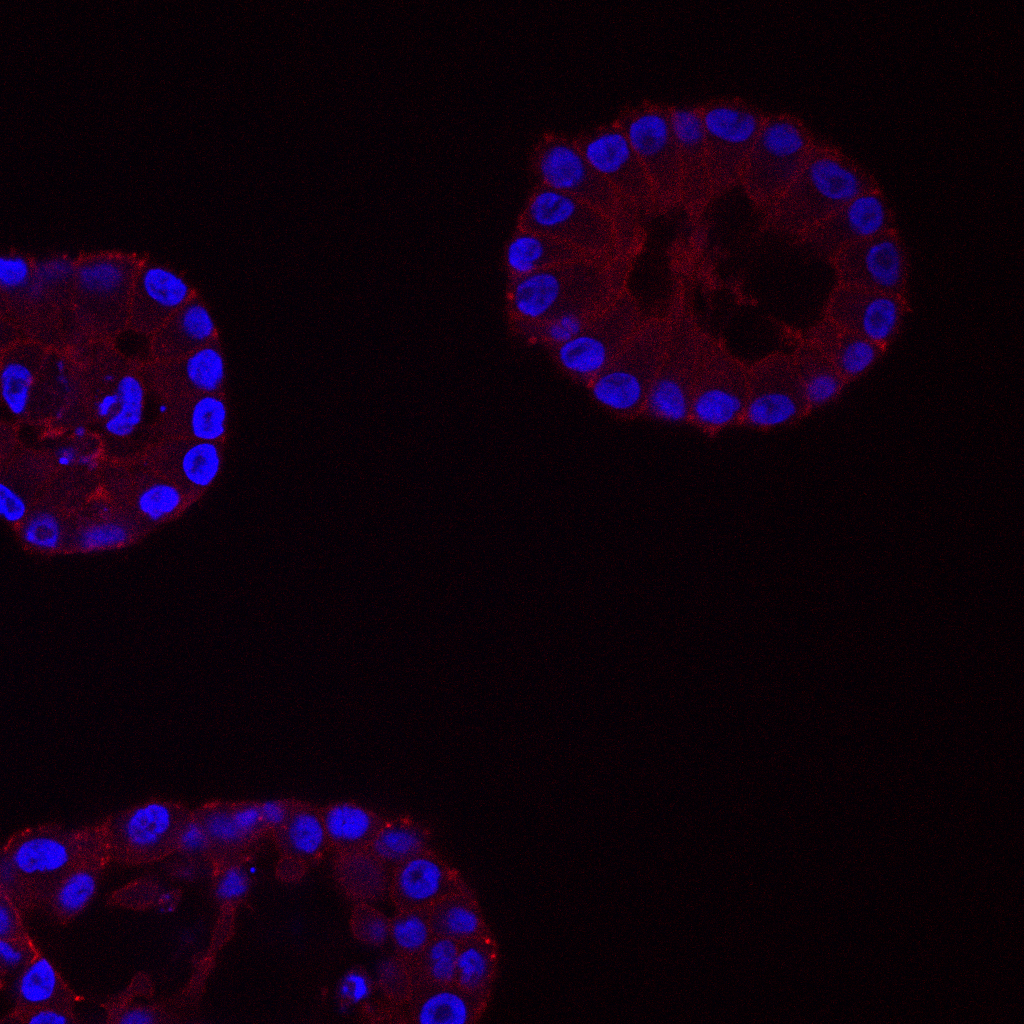

Supplement: Supplementary file 10 — Source Data for Figure 4 [file EMMM-12-e10491-s008.zip › Fig4/Fig_4B_M1D_+_amlexanox.TIF]

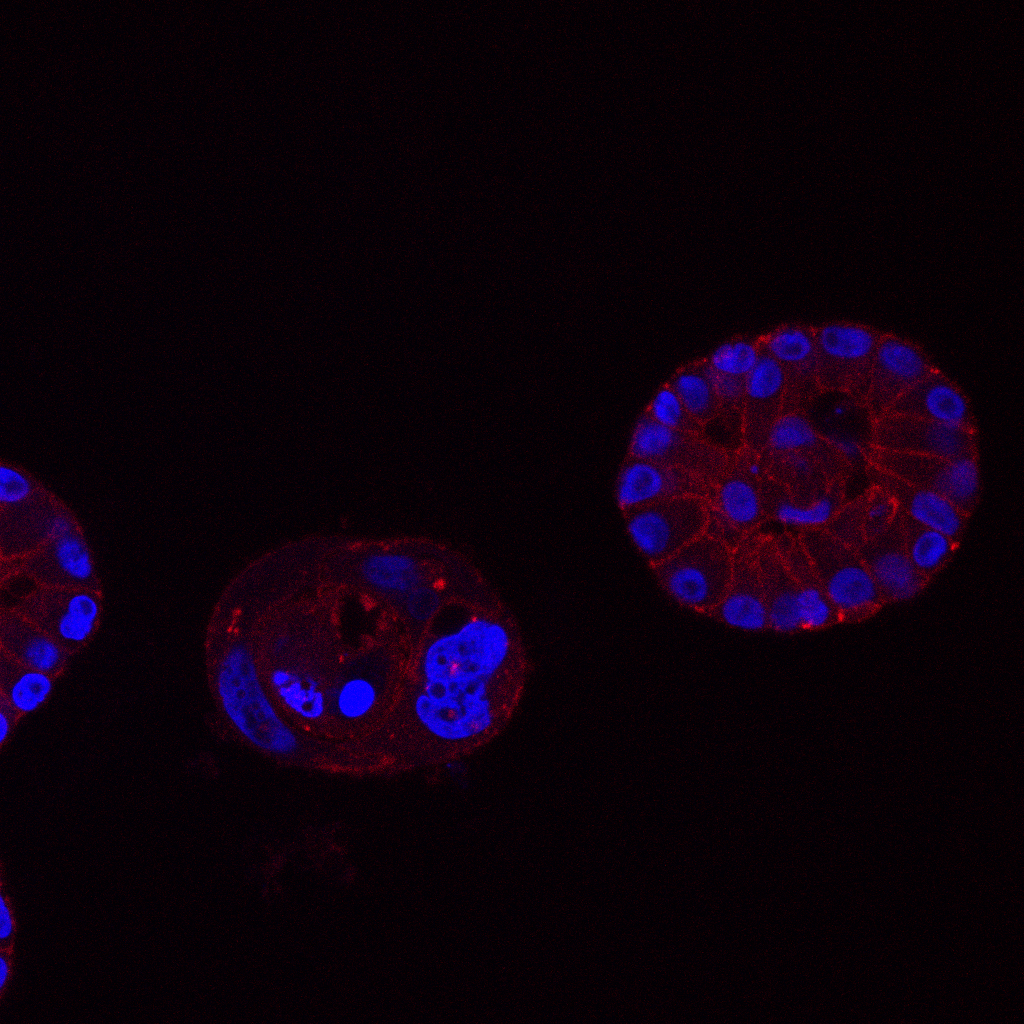

Supplement: Supplementary file 10 — Source Data for Figure 4 [file EMMM-12-e10491-s008.zip › Fig4/Fig_4B_M2A.TIF]

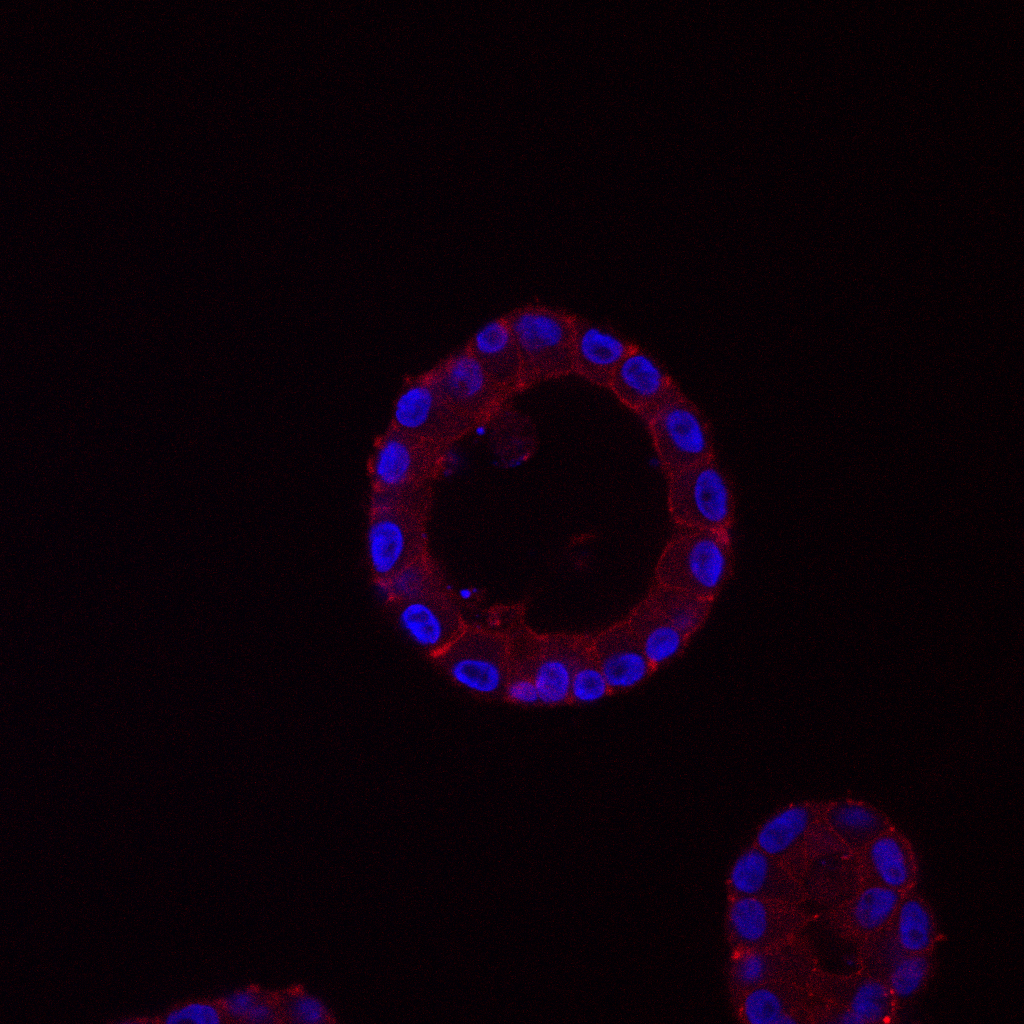

Supplement: Supplementary file 10 — Source Data for Figure 4 [file EMMM-12-e10491-s008.zip › Fig4/Fig_4B_M2A_+_amlexanox.TIF]

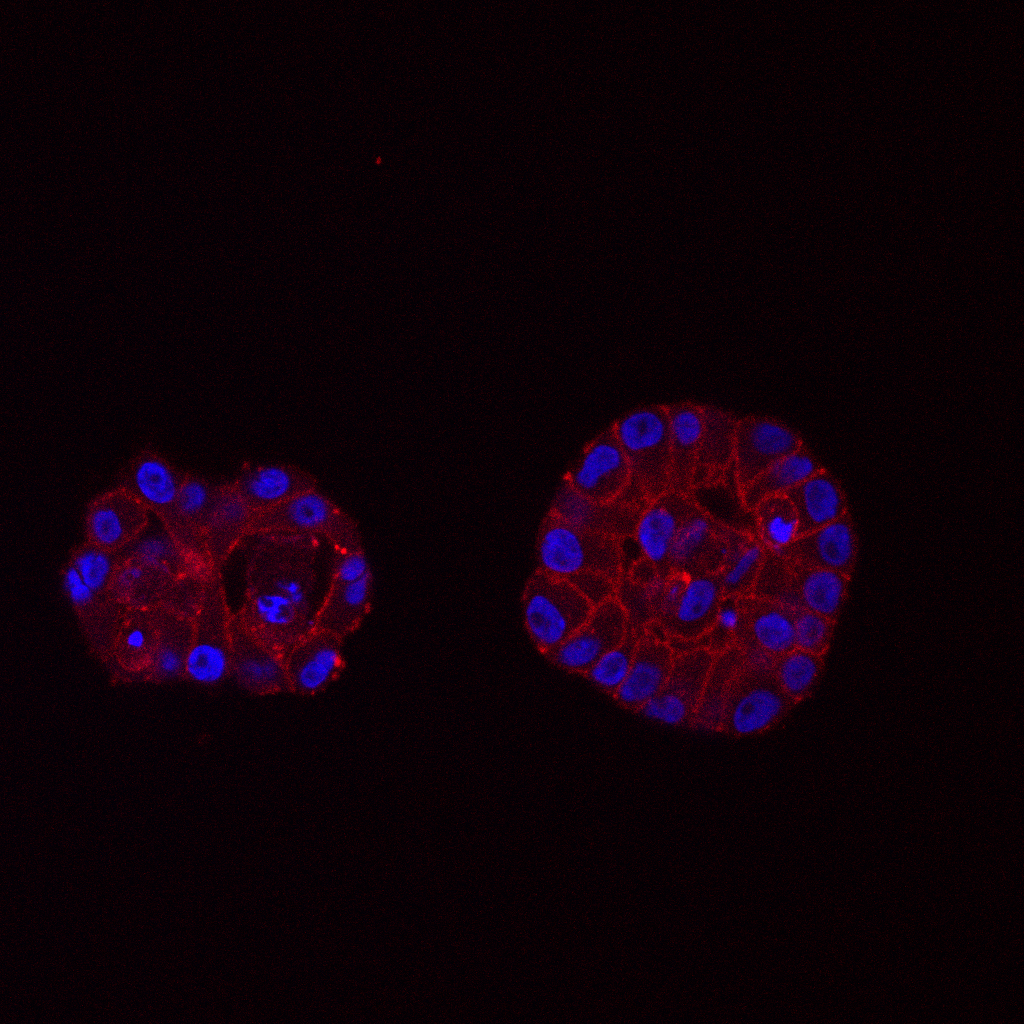

Supplement: Supplementary file 10 — Source Data for Figure 4 [file EMMM-12-e10491-s008.zip › Fig4/Fig_4B_M2D.TIF]

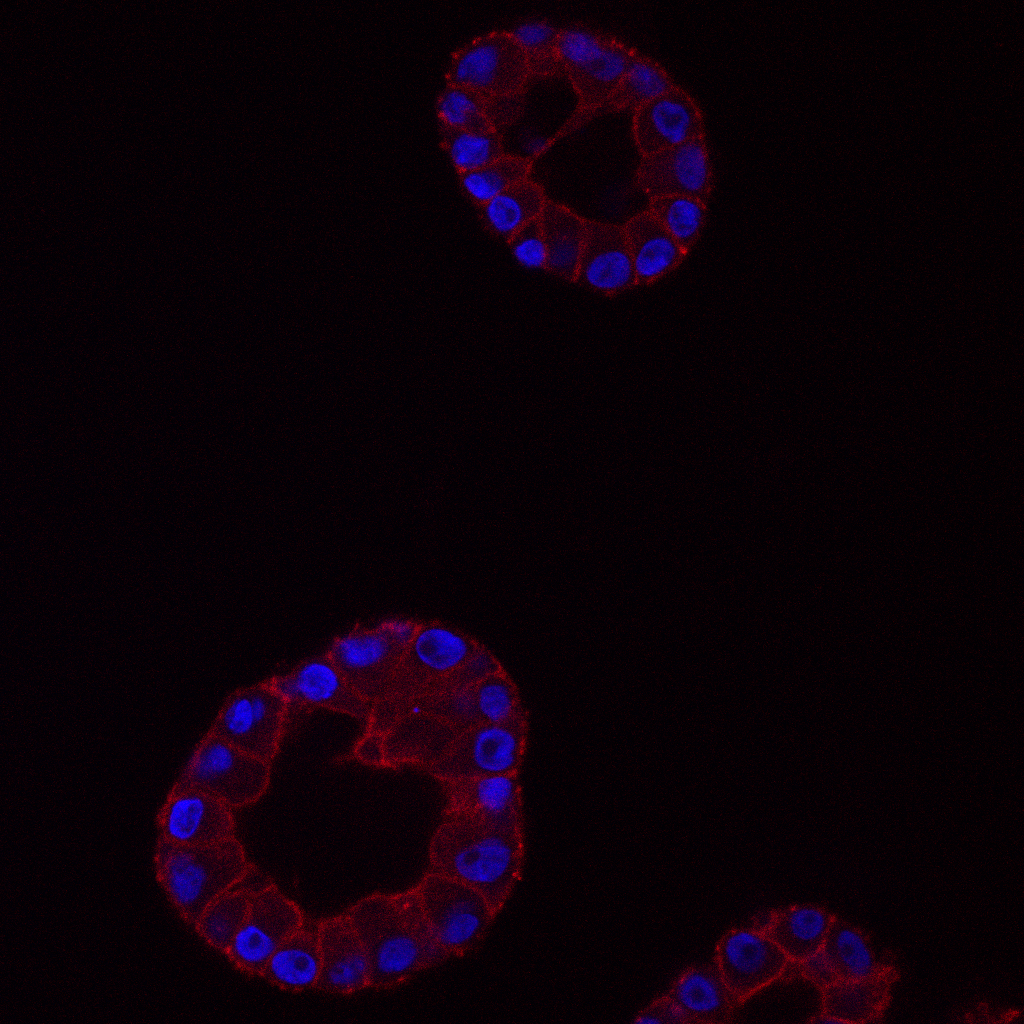

Supplement: Supplementary file 10 — Source Data for Figure 4 [file EMMM-12-e10491-s008.zip › Fig4/Fig_4B_M2D_+_amlexanox.TIF]

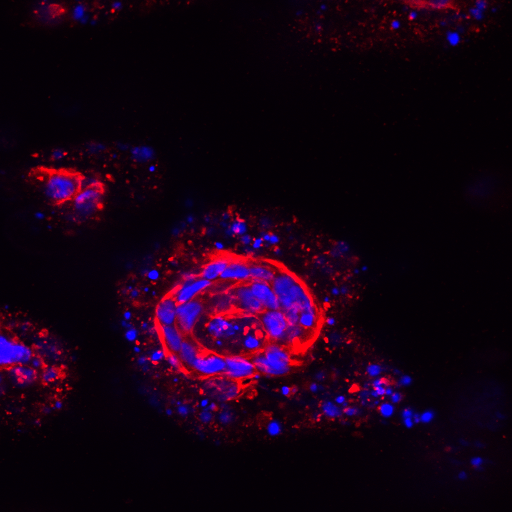

Supplement: Supplementary file 10 — Source Data for Figure 4 [file EMMM-12-e10491-s008.zip › Fig4/Fig_4I_control.TIF]

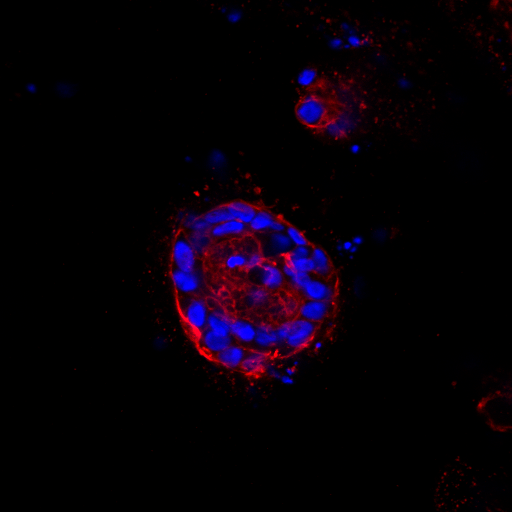

Supplement: Supplementary file 10 — Source Data for Figure 4 [file EMMM-12-e10491-s008.zip › Fig4/Fig_4I_control_+_amlexanox.TIF]

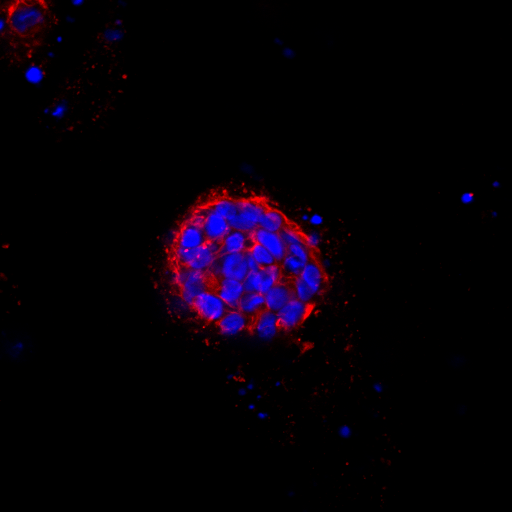

Supplement: Supplementary file 10 — Source Data for Figure 4 [file EMMM-12-e10491-s008.zip › Fig4/Fig_4I_M1A.TIF]

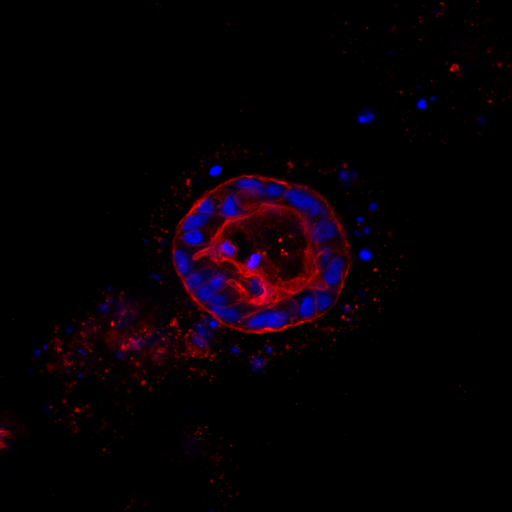

Supplement: Supplementary file 10 — Source Data for Figure 4 [file EMMM-12-e10491-s008.zip › Fig4/Fig_4I_M1A_+amlexanox.TIF]

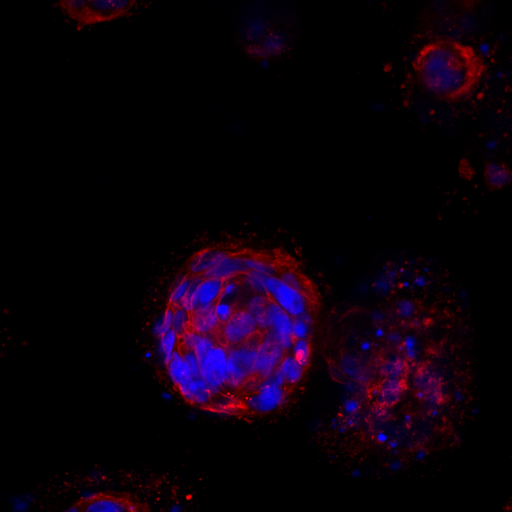

Supplement: Supplementary file 10 — Source Data for Figure 4 [file EMMM-12-e10491-s008.zip › Fig4/Fig_4I_M2A.TIF]

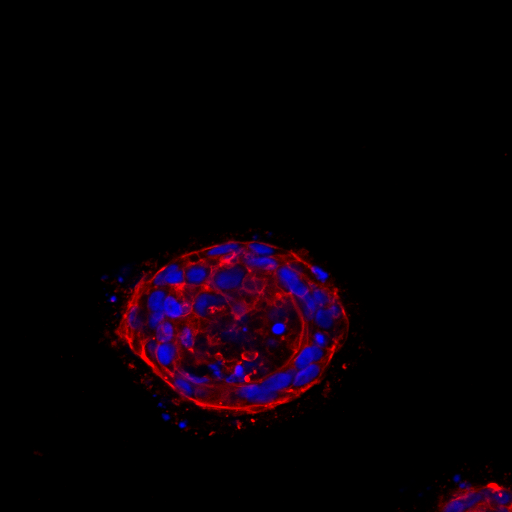

Supplement: Supplementary file 10 — Source Data for Figure 4 [file EMMM-12-e10491-s008.zip › Fig4/Fig_4I_M2A_+amlexanox.TIF]

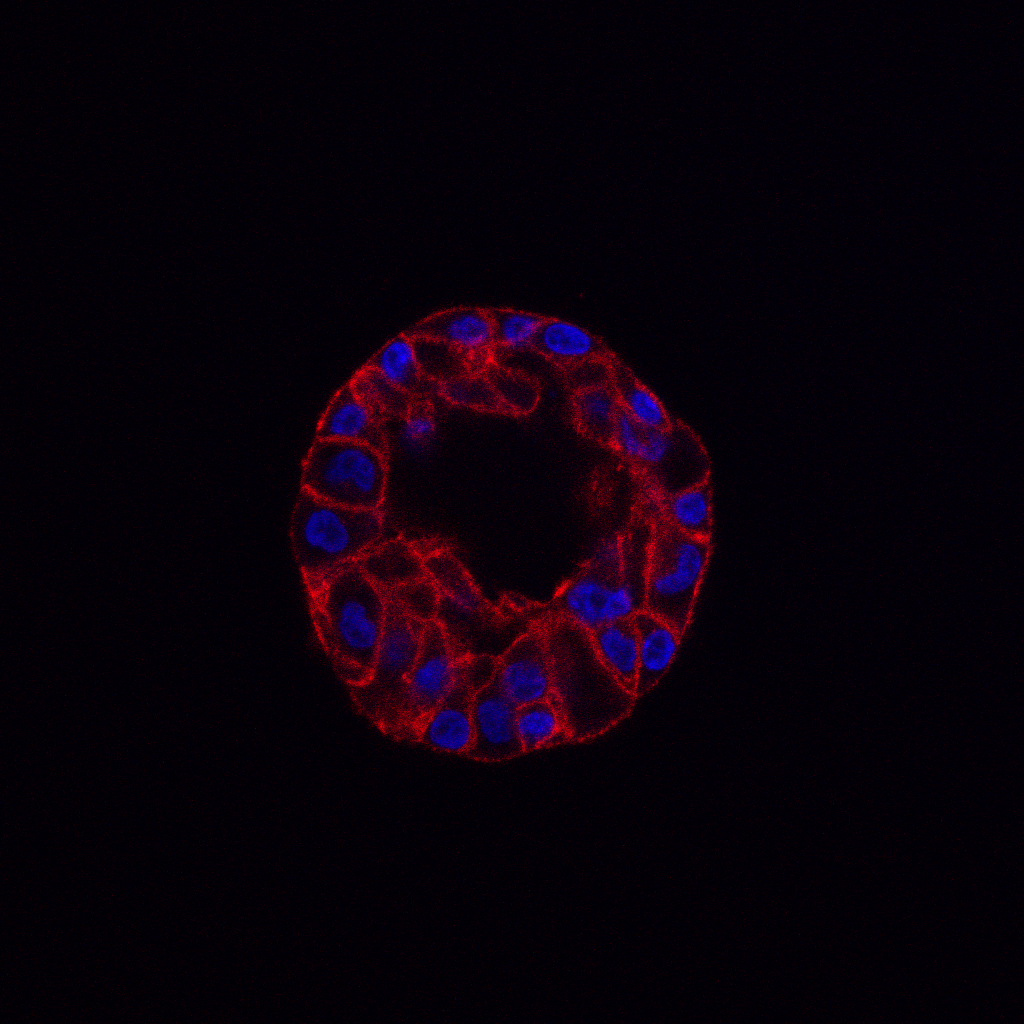

Supplement: Supplementary file 12 — Source Data for Figure 6 [file EMMM-12-e10491-s010.zip › Fig6/Fig_6B_control.TIF]

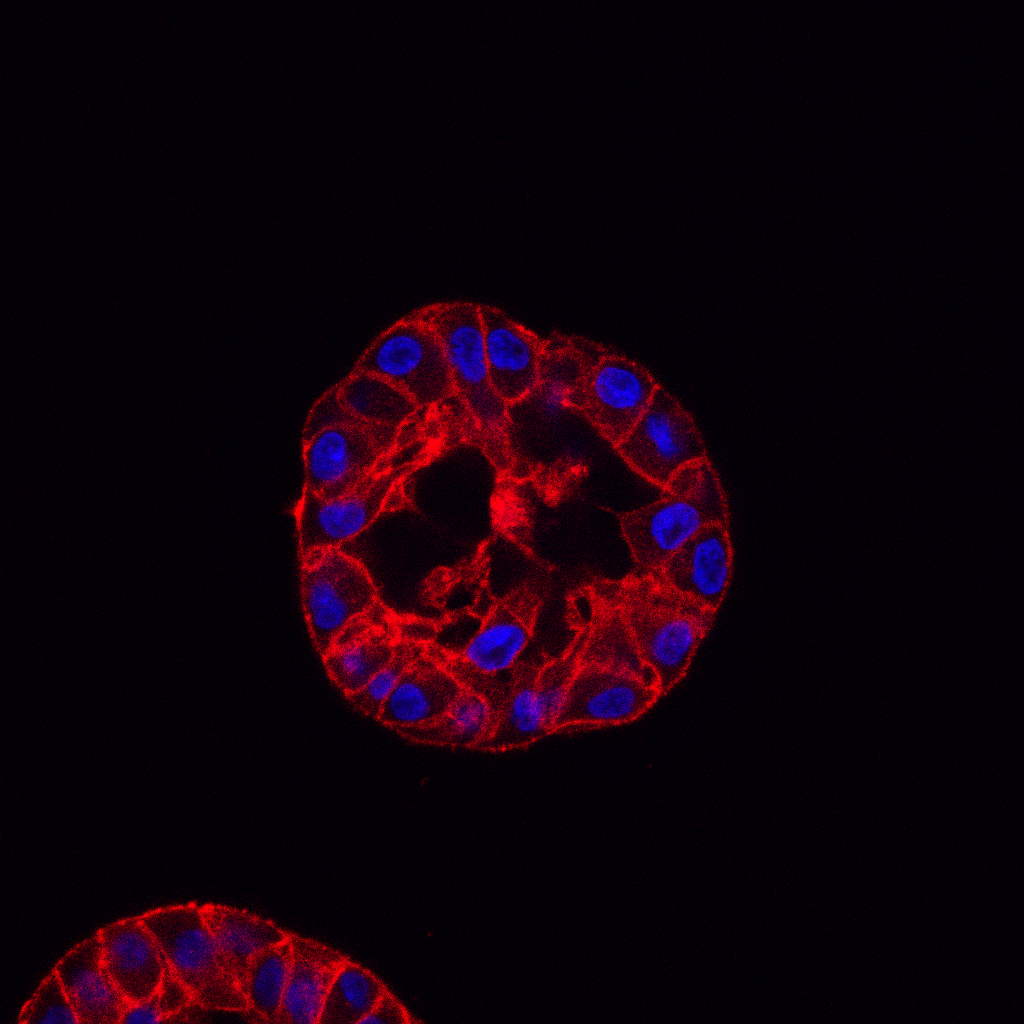

Supplement: Supplementary file 12 — Source Data for Figure 6 [file EMMM-12-e10491-s010.zip › Fig6/Fig_6B_control_+_NCT502.TIF]
