# Supplementary figures and images for: Macrophages induce malignant traits in mammary epithelium via IKKε/TBK1 kinases and the serine biosynthesis pathway (part 2 of 2)
Source: EMBO Mol Med. 2020 Jan 13;12(2):e10491. doi: 10.15252/emmm.201910491 (PMC7005540; doi:10.15252/emmm.201910491)

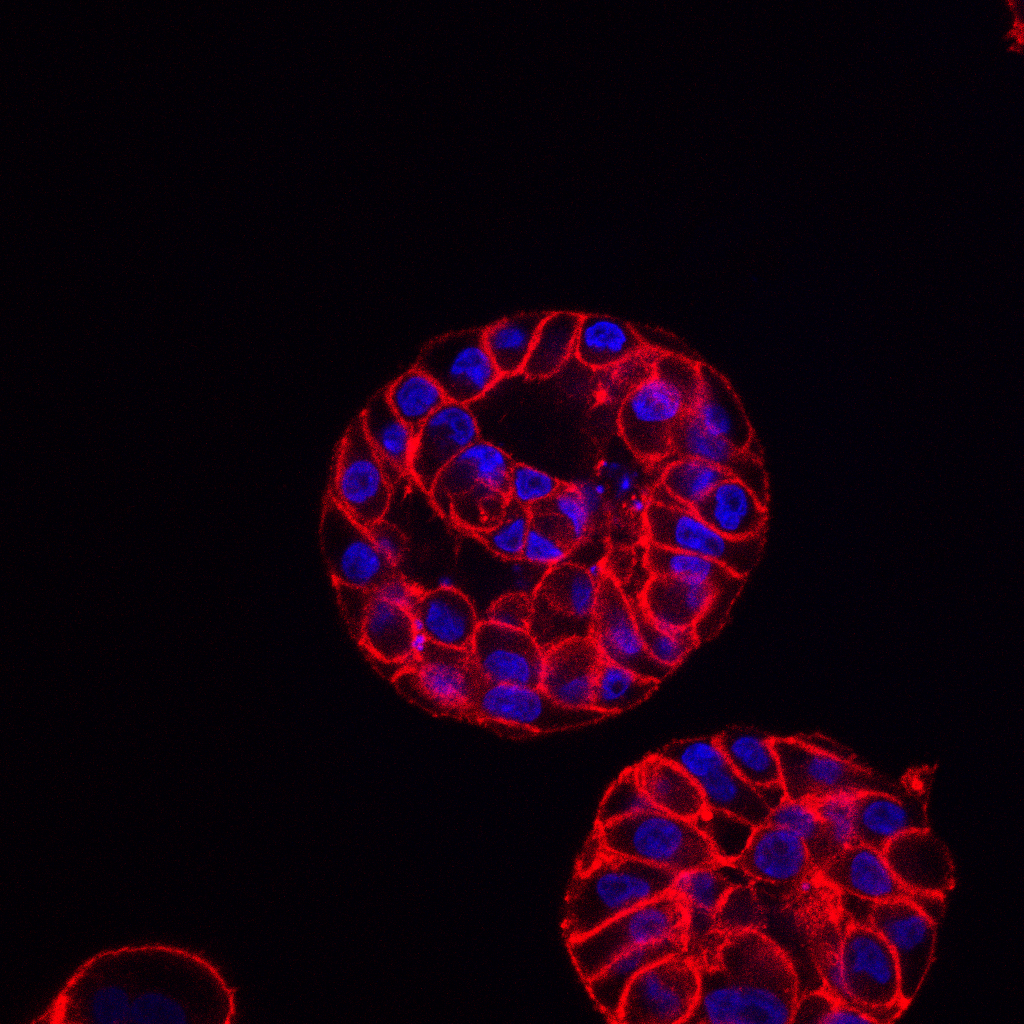

Supplement: Supplementary file 12 — Source Data for Figure 6 [file EMMM-12-e10491-s010.zip › Fig6/Fig_6B_M1A.TIF]

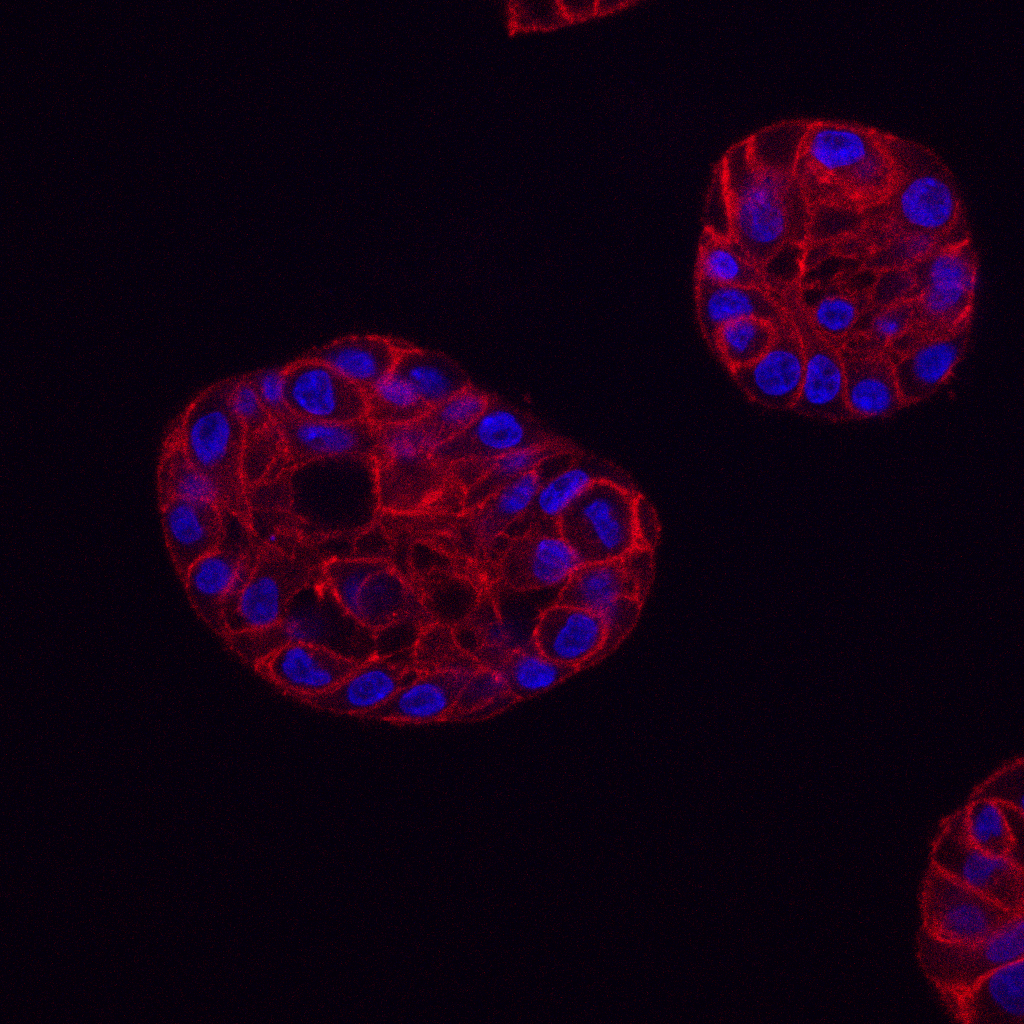

Supplement: Supplementary file 12 — Source Data for Figure 6 [file EMMM-12-e10491-s010.zip › Fig6/Fig_6B_M1A_+_NCT502.TIF]

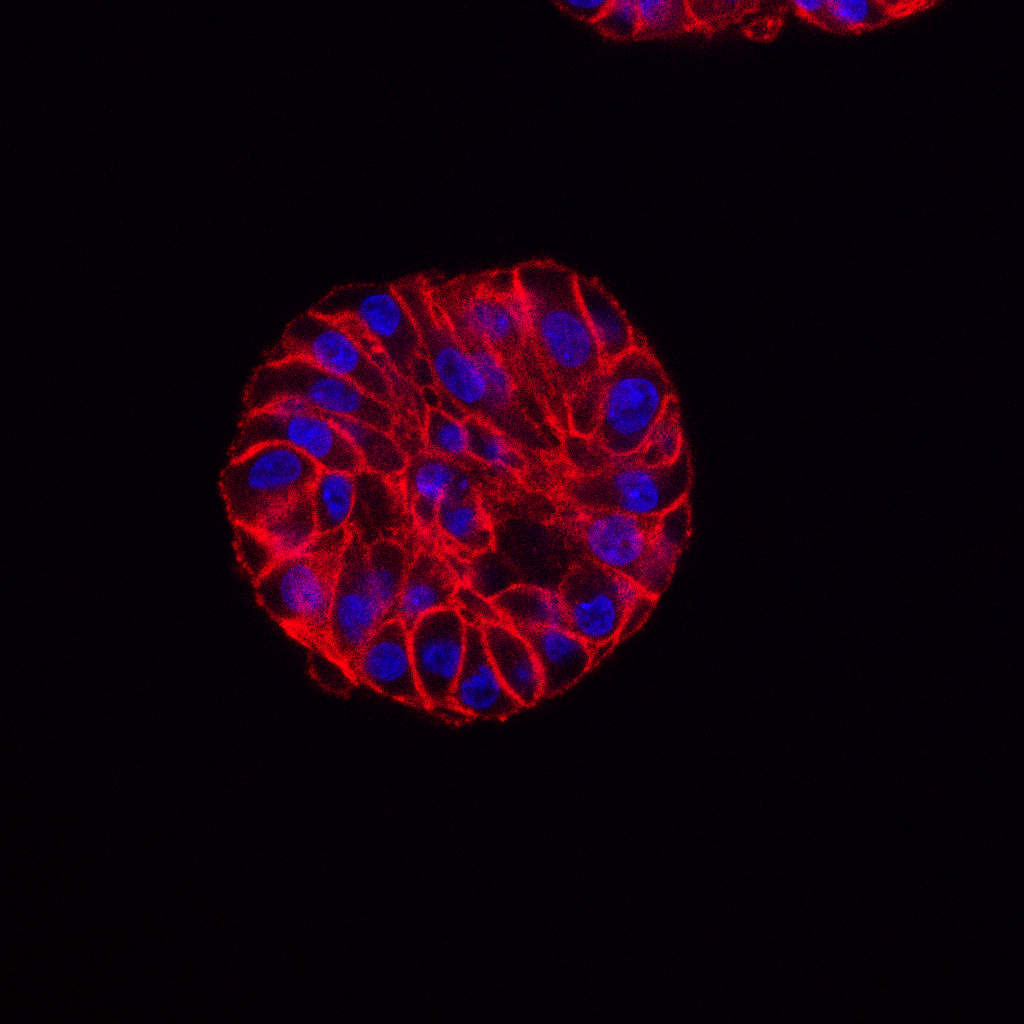

Supplement: Supplementary file 12 — Source Data for Figure 6 [file EMMM-12-e10491-s010.zip › Fig6/Fig_6B_M1D.TIF]

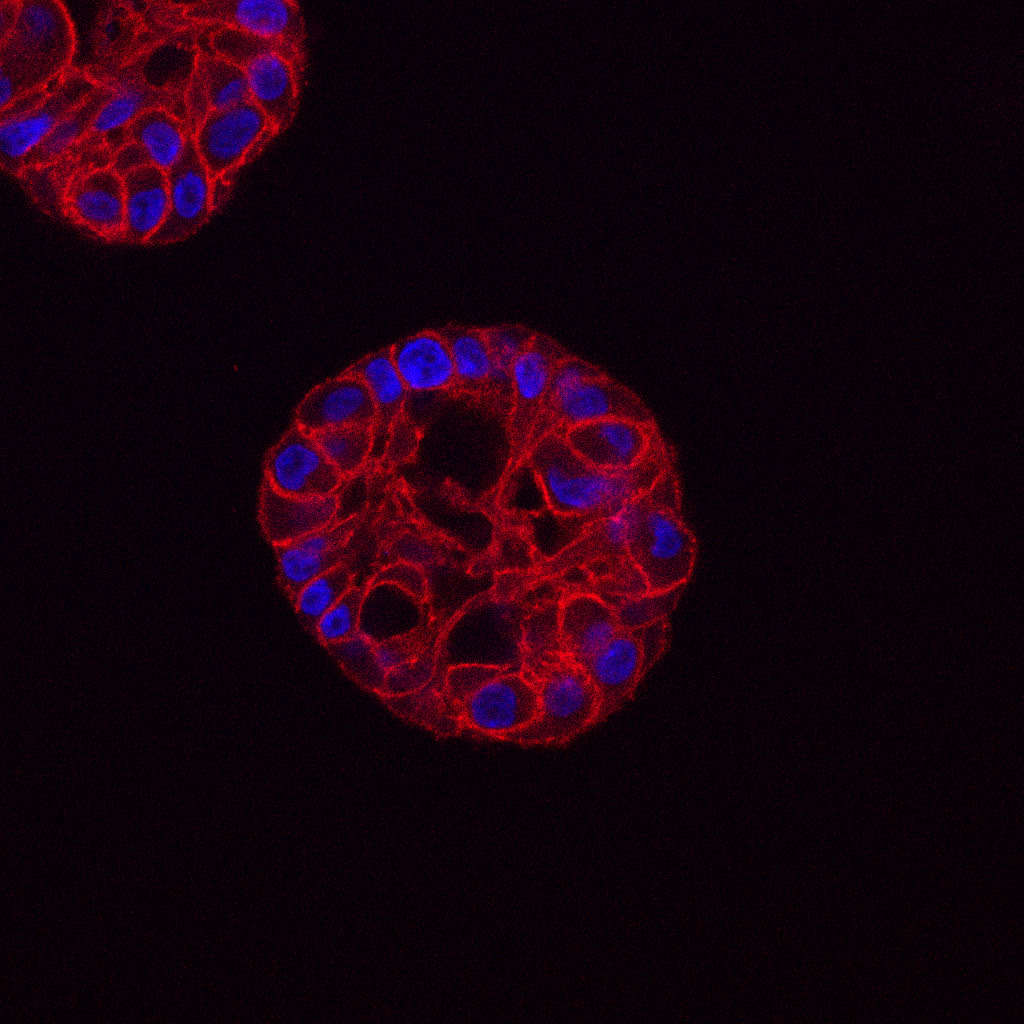

Supplement: Supplementary file 12 — Source Data for Figure 6 [file EMMM-12-e10491-s010.zip › Fig6/Fig_6B_M1D_+_NCT502.TIF]

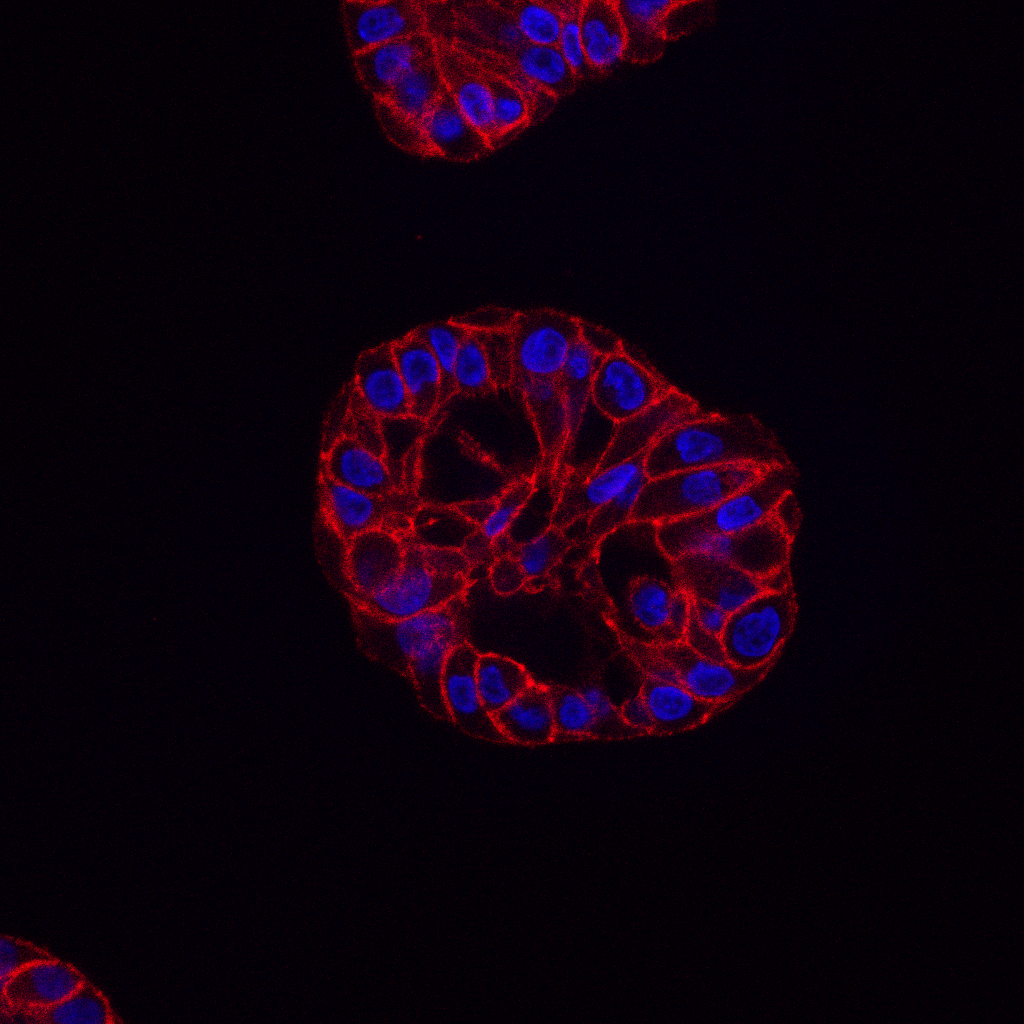

Supplement: Supplementary file 12 — Source Data for Figure 6 [file EMMM-12-e10491-s010.zip › Fig6/Fig_6B_M2A.TIF]

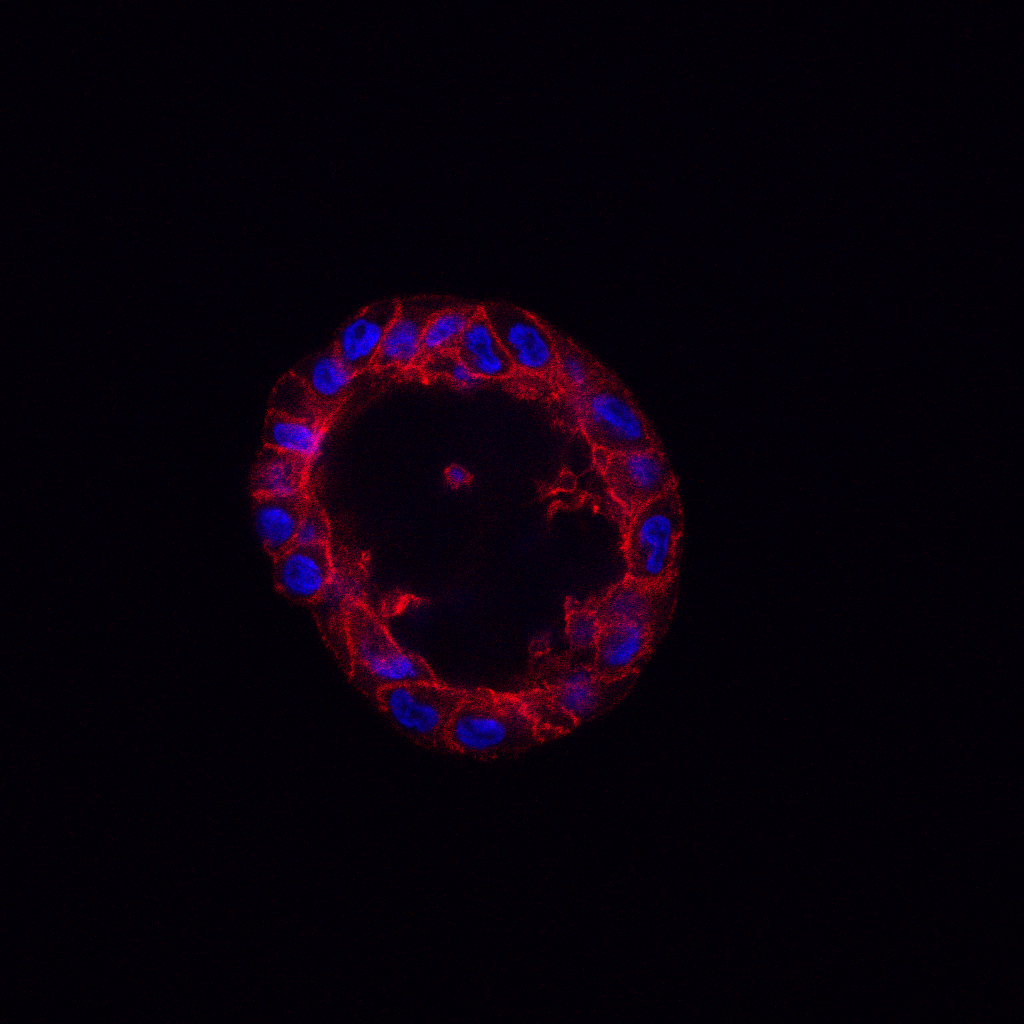

Supplement: Supplementary file 12 — Source Data for Figure 6 [file EMMM-12-e10491-s010.zip › Fig6/Fig_6B_M2A_+_NCT502.TIF]

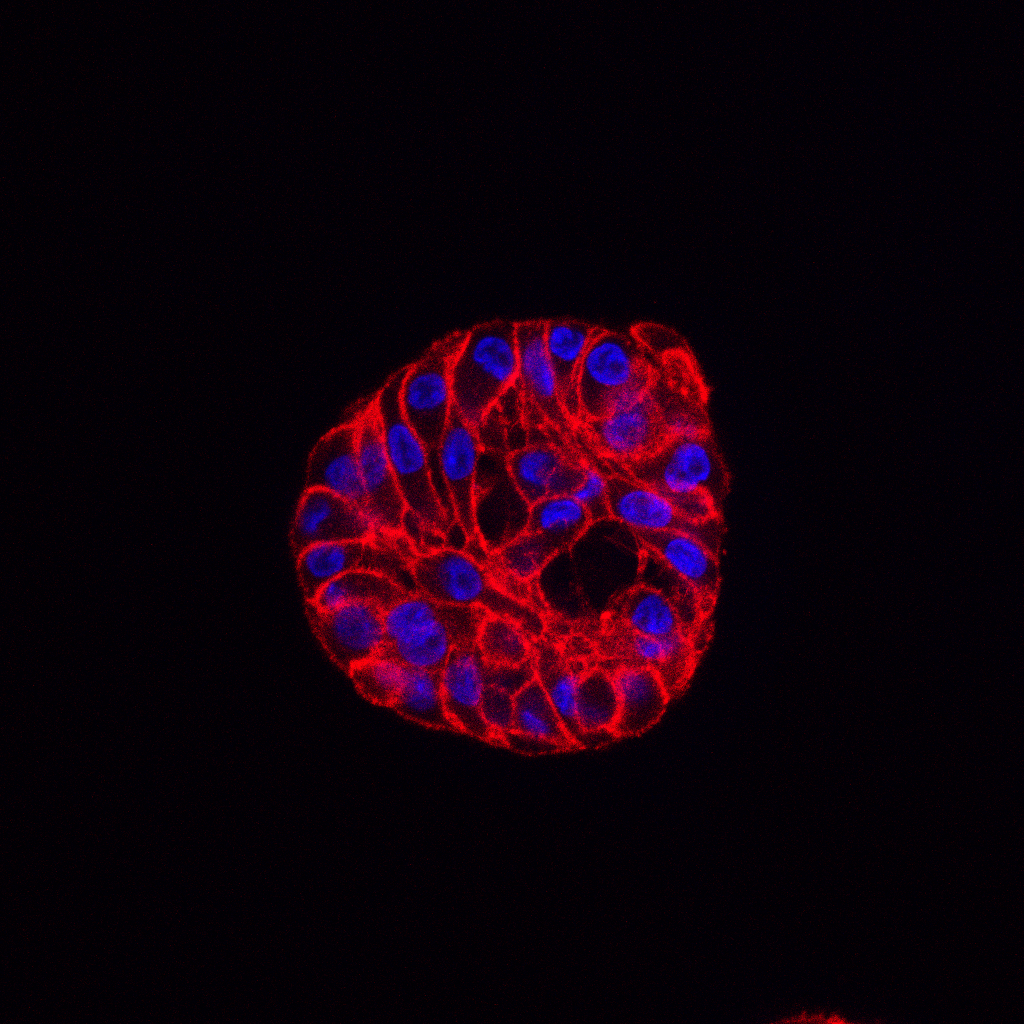

Supplement: Supplementary file 12 — Source Data for Figure 6 [file EMMM-12-e10491-s010.zip › Fig6/Fig_6B_M2D.TIF]

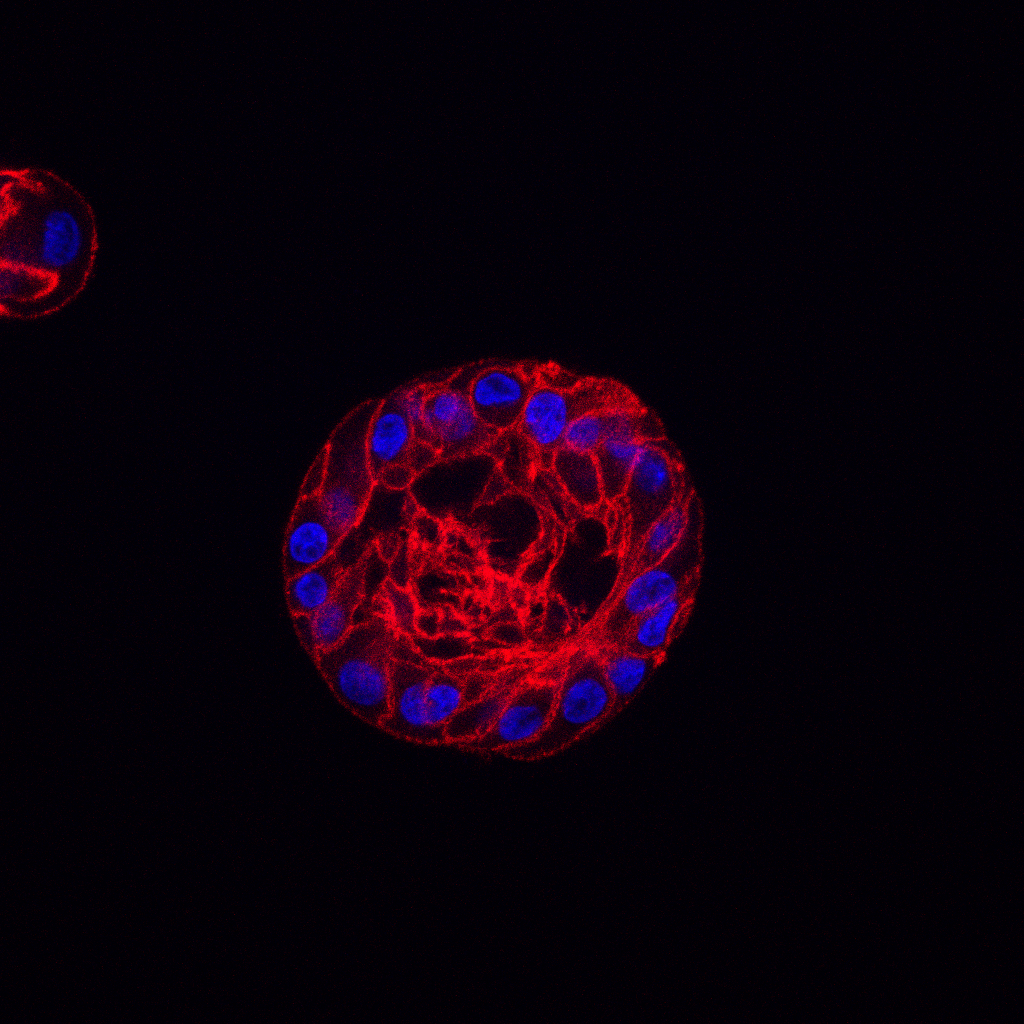

Supplement: Supplementary file 12 — Source Data for Figure 6 [file EMMM-12-e10491-s010.zip › Fig6/Fig_6B_M2D_+_NCT502.TIF]

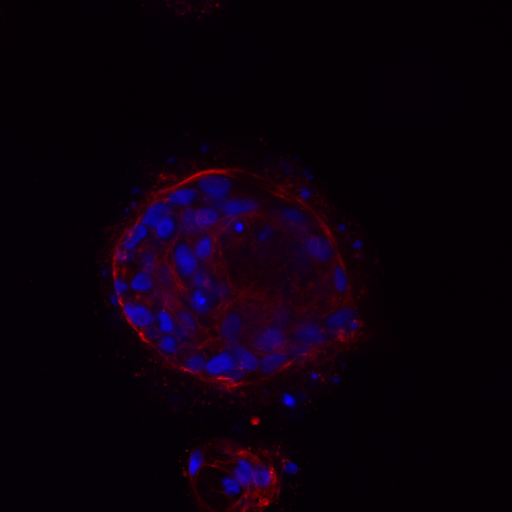

Supplement: Supplementary file 12 — Source Data for Figure 6 [file EMMM-12-e10491-s010.zip › Fig6/Fig_6I_control.TIF]

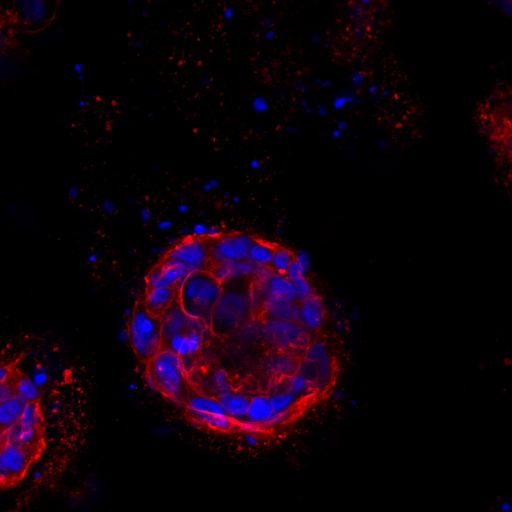

Supplement: Supplementary file 12 — Source Data for Figure 6 [file EMMM-12-e10491-s010.zip › Fig6/Fig_6I_control_+_NCT502.TIF]

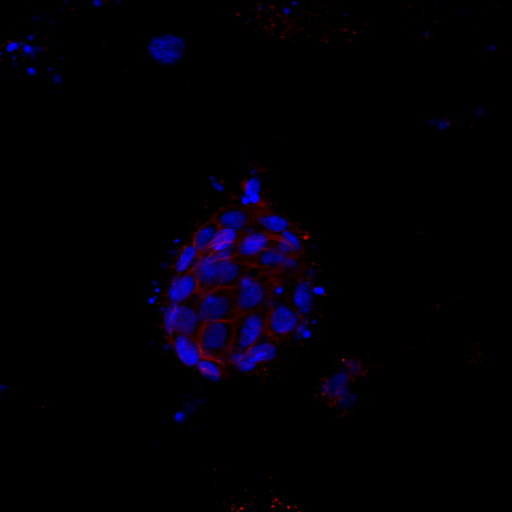

Supplement: Supplementary file 12 — Source Data for Figure 6 [file EMMM-12-e10491-s010.zip › Fig6/Fig_6I_M1A.TIF]

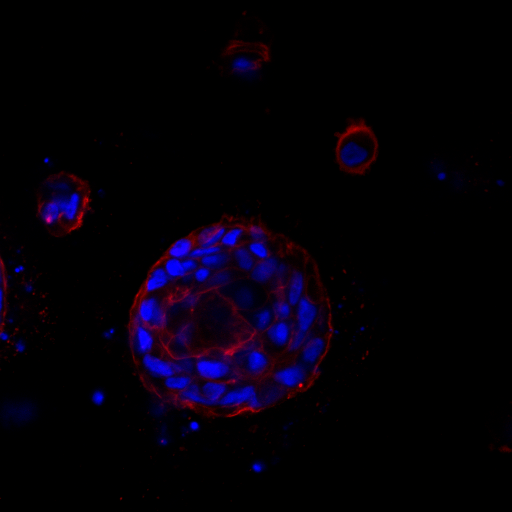

Supplement: Supplementary file 12 — Source Data for Figure 6 [file EMMM-12-e10491-s010.zip › Fig6/Fig_6I_M1A_+_NCT502.TIF]

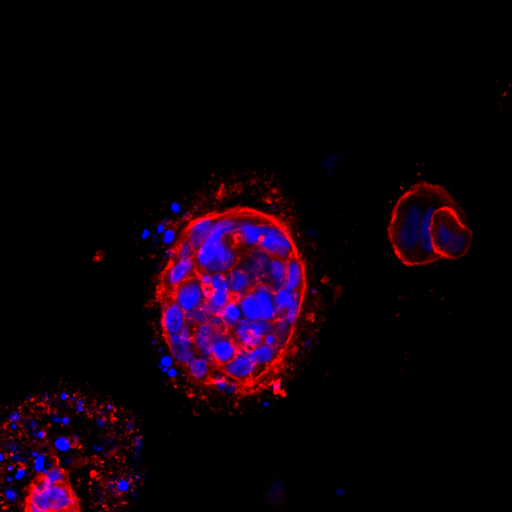

Supplement: Supplementary file 12 — Source Data for Figure 6 [file EMMM-12-e10491-s010.zip › Fig6/Fig_6I_M2A.TIF]

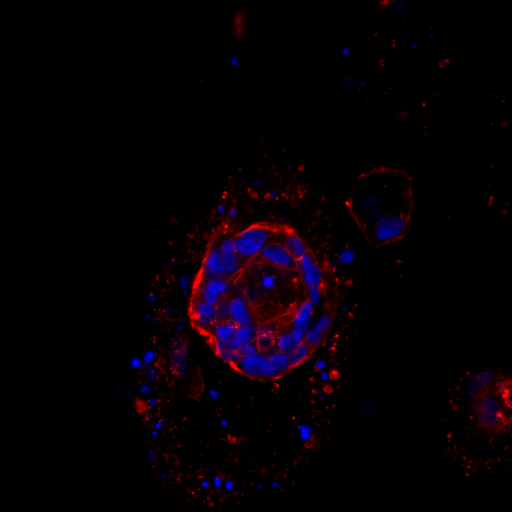

Supplement: Supplementary file 12 — Source Data for Figure 6 [file EMMM-12-e10491-s010.zip › Fig6/Fig_6I_M2A_+_NCT502.TIF]
